# Specific radiation damage to halogenated inhibitors and ligands in protein–ligand crystal structures

**DOI:** 10.1107/S1600576724010549

**Published:** 2024-11-26

**Authors:** Matthew J. Rodrigues, Marc Cabry, Gavin Collie, Michael Carter, Craig McAndrew, Robin L. Owen, Benjamin R. Bellenie, Yann-Vaï Le Bihan, Rob L. M. van Montfort

**Affiliations:** a Centre for Cancer Drug Discovery The Institute of Cancer Research 15 Cotswold Road Sutton LondonSM2 5NG United Kingdom; b Division of Structural Biology The Institute of Cancer Research LondonSW3 6JB United Kingdom; c Diamond Light Source Harwell Science and Innovation Campus DidcotOX11 0DE United Kingdom; Uppsala University, Sweden; The European Extreme Light Infrastucture, Czechia

**Keywords:** radiation damage, protein–ligand complexes, halogenated ligands, structure-based drug design

## Abstract

Protein–inhibitor crystal structures aid medicinal chemists in efficiently improving the potency and selectivity of small-molecule inhibitors. It is estimated that a quarter of lead molecules in drug discovery projects are halogenated. Protein–inhibitor crystal structures have shed light on the role of halogen atoms in ligand binding. They form halogen bonds with protein atoms and improve shape complementarity of inhibitors with protein binding sites. However, specific radiation damage (SRD) can cause cleavage of carbon–halogen (C–*X*) bonds during X-ray diffraction data collection. This study shows significant C–*X* bond cleavage in protein–ligand structures of the therapeutic cancer targets B-cell lymphoma 6 (BCL6) and heat shock protein 72 (HSP72) complexed with halogenated ligands, which is dependent on the type of halogen and chemical structure of the ligand. The study found that metrics used to evaluate the fit of the ligand to the electron density deteriorated with increasing X-ray dose, and that SRD eliminated the anomalous signal from brominated ligands. A point of diminishing returns is identified, where collecting highly redundant data reduces the anomalous signal that may be used to identify binding sites of low-affinity ligands or for experimental phasing. Straightforward steps are proposed to mitigate the effects of C–*X* bond cleavage on structures of proteins bound to halogenated ligands and to improve the success of anomalous scattering experiments.

## Abstract 2

This article reports an investigation into the effects of specific radiation damage to halogenated ligands in crystal structures of protein–inhibitor complexes.

## 1. Introduction

A fundamental problem in macromolecular X-ray crystallography is the damage caused by the ionizing radiation used to obtain diffraction data. Despite the current practice of collecting data from cryocooled crystals, radiation damage is routinely observed for data collected using the intense X-ray beams generated at modern third- and fourth-generation synchrotrons. Radiation damage is caused by the generation of solvated electrons and free radical species upon absorption of X-rays, which propagate through crystals and react with protein molecules (Nave, 1995 ▸; Close & Bernhard, 2019 ▸). The effects manifest themselves at both a global and a specific level. Global radiation damage (GRD) is observed as an expansion of the unit-cell volume, an increase in mosaicity, higher Wilson *B* factors and a general loss of diffraction intensity with concomitant loss of data resolution (Garman, 2010 ▸). Specific radiation damage (SRD) becomes apparent in electron-density maps during structure refinement and includes the elongation or breakage of disulfide bonds, decarboxylation of acidic residues, reduction of metal centres, loss of the methylthio group in methionine and of the methylseleno group in selenomethionine residues, and loss or disorder of the sulfhydryl group in cysteine residues (Holton, 2007 ▸; Garman, 2010 ▸; Weik *et al.*, 2000 ▸; Burmeister, 2000 ▸; Close & Bernhard, 2019 ▸). In turn, this can lead to unsuccessful experimental phasing, errors in protein structures deposited in the Protein Data Bank (PDB; https://www.rcsb.org/) and incorrect structure–function interpretations.

The effects of radiation damage on proteins have been well characterized (Garman & Weik, 2019 ▸). However, despite the extensive use of X-ray crystallography in structure-based drug design, the effects of SRD on inhibitors and other protein-bound ligands are poorly documented. Nevertheless, there is some literature evidence that radiation damage also affects bound ligands. For example, a study to characterize radiation damage to bovine trypsin structures also found X-ray-induced changes of the bound benzylamine ligand (Ravelli *et al.*, 2003 ▸). In addition, X-ray-induced cleavage of carbon–bromine bonds has been observed in anomalous scattering experiments on crystals of brominated oligonucleotides and crystals of proteinase K complexed with the brominated small-molecule ligand 5-amino-2,4,6-tri­bromoisophthalic acid (Beck *et al.*, 2010 ▸; Ennifar *et al.*, 2002 ▸).

The X-ray-induced cleavage of carbon–halogen (C–*X*) bonds is of particular importance because a significant number of marketed drugs and clinical candidates contain at least one covalently bound halogen atom (Hernandes *et al.*, 2010 ▸). In addition, 14 out of 50 drugs (28%) approved by the US Food and Drug Association (FDA) in 2021 contained halogen atoms (Benedetto Tiz *et al.*, 2022 ▸). Moreover, an analysis in March 2019 revealed that approximately a quarter (5950) of the non-redundant ligands in the PDB contained halogen atoms (Shinada *et al.*, 2019 ▸). Therefore, X-ray-induced cleavage of C–*X* bonds may have affected a significant proportion of protein–ligand structures in the PDB. Indeed, in several of our own protein–inhibitor structures we have observed strong neg­a­tive *mFo*–*DFc* difference density peaks at halogen positions in our ligands, which we suspected to be the result of SRD. We were concerned that not treating this type of SRD properly could result in a reduction of the quality of local electron density and consequently in a sub-optimal fit of bound ligands. Moreover, the reliable detection, placement and refinement of fragments from halogen-enriched fragment libraries (Blaney *et al.*, 2006 ▸; Tiefenbrunn *et al.*, 2014 ▸; Wilcken *et al.*, 2012 ▸; Wood *et al.*, 2019 ▸) could be severely affected by X-ray-induced cleavage of C–*X* bonds, as has been recently reported (Ma *et al.*, 2024 ▸).

**Figure 1:**
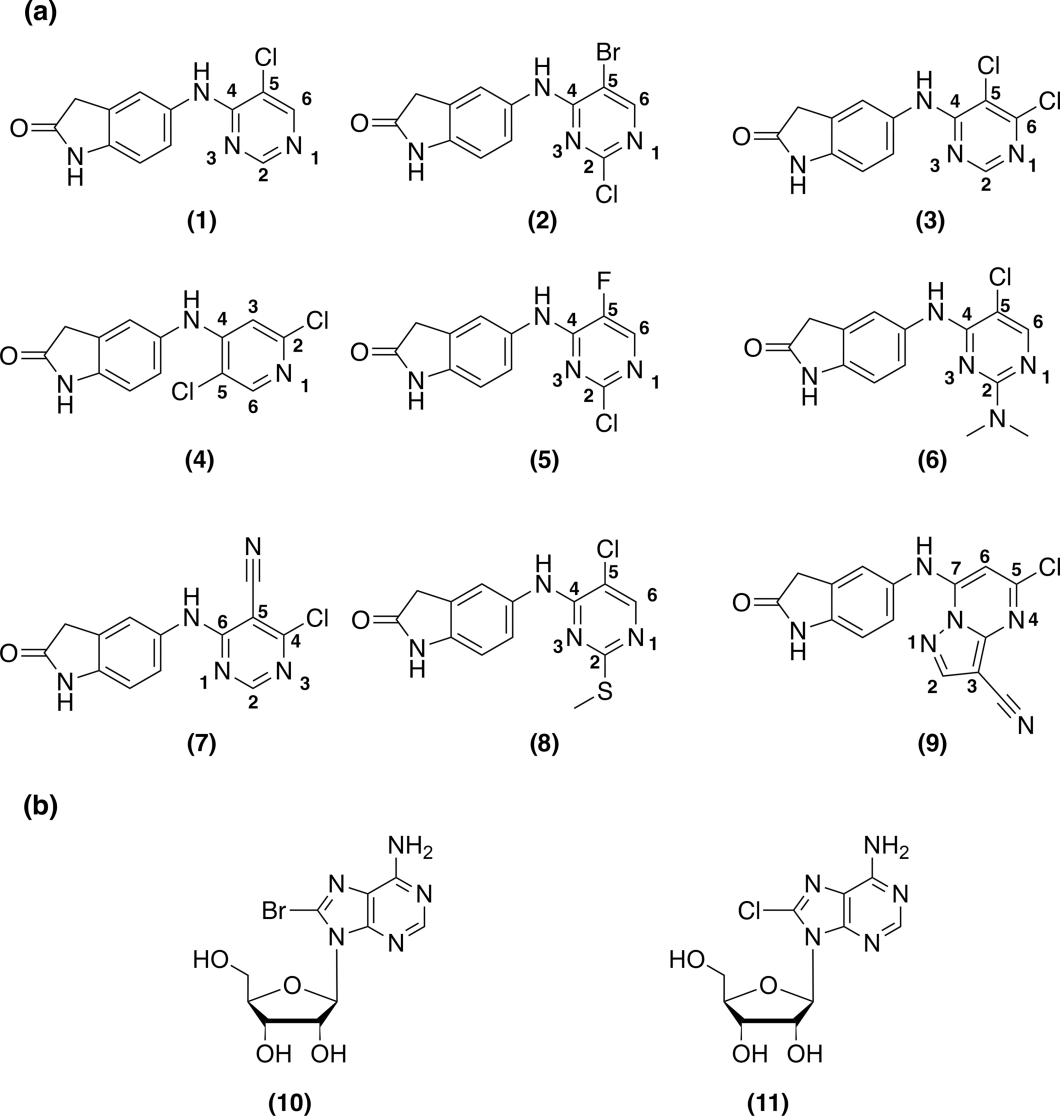
(*a*) Chemical structures of BCL6 ligands (**1**–**9**) used for radiation damage experiments. (*b*) Chemical structures of HSP72 ATP binding site ligands 8-bromoadenosine (**10**) and 8-chloroadenosine (**11**) used for radiation damage experiments.

Therefore, we embarked on a systematic study into the effects of synchrotron radiation on protein-bound halogenated small-molecule ligands. We conducted our studies on crystals of the BTB domain of the transcriptional repressor and therapeutic cancer target B-cell lymphoma 6 (BCL6) (Cerchietti *et al.*, 2010 ▸), complexed to nine inhibitors from our drug discovery programme (Bellenie *et al.*, 2020 ▸; Pierrat *et al.*, 2022 ▸), which share the same core scaffold but have different halogen substitution patterns [Fig. 1 ▸(*a*)]. In addition, we analysed crystals of the stress-induced molecular chaperone and therapeutic cancer target heat shock protein 72 (HSP72) (Powers *et al.*, 2008 ▸; Jones *et al.*, 2016 ▸) complexed to two different halogenated adenosine analogues [Fig. 1 ▸(*b*)]. Using a multiple serial structures from one crystal (MSOX) approach (Horrell *et al.*, 2018 ▸), we observed X-ray-induced C–*X* bond cleavage of all BCL6 inhibitors and HSP72 ligands, with a concomitant loss of the anomalous signal of the respective halogen atoms. We also found that the sensitivity of a given C–*X* bond is dependent on the chemical structure of the ligand. We showed that metrics to evaluate the quality of ligand modelling in protein structures, such as the ligand real-space *R* factor, real-space correlation coefficient and average ligand *B* factor, deteriorated with increasing absorbed X-ray dose. On the basis of our findings, we recommend careful low-dose data collection strategies in combination with occupancy refinement of halogen atoms to reduce the impact of X-ray-induced C–*X* bond cleavage on the placement of ligands in protein–ligand structures and to improve the quality of the refined models. Implementation of these strategies should have a positive impact on structure-based drug design programmes and contribute to the optimal use of halogen-enriched fragment libraries.

## 2. Methods

### 2.1. Synthesis of ligands

BCL6 ligands **1**–**9** were prepared using known methods such as those reported by Kamada *et al.* (2017 ▸). Detailed preparations and spectroscopic data for BCL6 ligands can be found in the supporting information Section S1, *Synthesis of BCL6 Ligands*. HSP72 ligands **10** and **11** used for soaking experiments were purchased from Carbosynth (Compton, UK).

### 2.2. Expression, purification and crystallization of the BCL6 BTB domain

Expression, purification and crystallization of the BTB domain of human BCL6 (residues 5–129), cloned into a pET48b vector with N-terminal histidine and thioredoxin tags followed by a HRV-3C protease cleavage site, was carried out as previously described (Bellenie *et al.*, 2020 ▸; Pierrat *et al.*, 2022 ▸). Soaking experiments were carried out by the addition of 0.5 µl of each compound [10 m*M* dissolved in dimethyl sulfoxide (DMSO)] directly to the crystallization drops, followed by 30 min incubation. Crystals were harvested and cryoprotected in reservoir solution supplemented with 30%(*v*/*v*) ethylene glycol and cryocooled in liquid nitrogen.

### 2.3. Expression, purification and crystallization of HSP72

#### 2.3.1. HSP72 expression

BL21-AI *Escherichia coli* cells were transformed with a previously reported plasmid coding for residues 1–380 of HSP72 with an N-terminal GST tag followed by a HRV-3C protease cleavage site (Jones *et al.*, 2016 ▸). Cells were grown to an OD_600 nm_ of 0.6 in LB medium supplemented with 100 mg l^−1^ ampicillin. Protein expression was induced by the addition of 0.2 m*M* isopropyl β-d-1-thiogalacto­pyranoside (IPTG) and carried out at 18 °C for 18 h. Cells were harvested by centrifugation (5500*g* for 30 min at 4 °C) and re-suspended in a buffer composed of 25 m*M* Tris pH 7.5, 50 m*M* NaCl, 1 m*M* MgCl_2_, 1 m*M* DTT, 5%(*v*/*v*) glycerol, 12.5 units ml^−1^ benzonase and Roche cOmplete protease inhibitor cocktail.

#### 2.3.2. HSP72 purification

Cells were lysed by sonication followed by centrifugation at 21500*g* for 30 min at 4 °C and filtration using a 1.2 µm syringe-driven filter. The supernatant was loaded onto a GSTrap FF column, washed with 25 m*M* Tris pH 7.5, 50 m*M* NaCl, 1 m*M* DTT and 5%(*v*/*v*) glycerol, followed by 25 m*M* Tris pH 7.5, 500 m*M* NaCl, 1 m*M* DTT and 5%(*v*/*v*) glycerol. GST-HSP72 was eluted using 25 m*M* Tris pH 7.5, 50 m*M* NaCl, 1 m*M* DTT, 5%(*v*/*v*) glycerol and 20 m*M* glutathione and incubated with HRV-3C protease for 4 h at 4 °C. The resulting solution was loaded onto a HiLoad 26/60 Superdex200 column in series with a GSTrap FF pre-equilibrated with 25 m*M* Tris pH 7.5, 50 m*M* NaCl, 1 m*M* DDT and 5%(*v*/*v*) glycerol. Fractions containing cleaved HSP72 were further purified by reverse ion exchange using a Resource Q column in buffer containing 25 m*M* Tris pH 7.5, 1 m*M* DDT, 2 m*M* EDTA and 5%(*v*/*v*) glycerol. The flow-through from ion exchange containing HSP72 was loaded onto a HiLoad 26/60 Superdex200 column in buffer containing 25 m*M* Tris pH 7.5, 50 m*M* NaCl, 1 m*M* DDT and 5%(*v*/*v*) glycerol.

#### 2.3.3. HSP72 crystallization

HSP72 was buffer exchanged by diafiltration into 100 m*M* HEPES pH 7.5 and concentrated to 6 mg ml^−1^ using a centrifugal concentrator with a 10 kDa molecular weight cut-off. HSP72 ligands at a concentration of 100 m*M* in DMSO were added to purified HSP72 to a final concentration of 5 m*M* and incubated on ice for 30 min. Crystals were grown at 18 °C in sitting drops composed of 0.5 µl HSP72/compound complex plus 0.5 µl of reservoir solution [100 m*M* HEPES pH 7.5, 18–28%(*v*/*v*) PEG 3350, 2 m*M* MgCl_2_, 2 m*M* NaH_2_PO_4_]. Crystals were cryoprotected in Paratone-N and cryocooled in liquid nitrogen.

### 2.4. X-ray diffraction data collection

All datasets were collected at beamline I24, Diamond Light Source (Harwell Science and Innovation Campus, Didcot, UK). All diffraction data were collected at 100 K using a Pilatus3 6M detector (DECTRIS Ltd, Switzerland) with a 40 ms exposure time and 0.1° oscillation per image.

#### 2.4.1. BCL6 MSOX data collection

For each BCL6 BTB–inhibitor complex (compounds **1**–**9**) 15 datasets were collected from one crystal while continuously rotating the sample. Per dataset, 900 images were collected at a wavelength of 0.9686 Å (12.8 keV) and a flux of 3.0 × 10^10^ photons s^−1^. The X-ray beam was defocussed at the sample with a full width at half-maximum (FWHM) of 50 × 50 µm.

#### 2.4.2. HSP72 anomalous scattering and MSOX data collection

Co-crystals of HSP72 in complex with ligands **10** and **11** were used for X-ray diffraction experiments. A XANES scan was performed on a sacrificial HSP72 co-crystal with ligand **10** to determine the Br *K*-edge energy (Fig. S1). Subsequently, all datasets from HSP72 crystals were collected at a wavelength of 0.9192 Å (13.49 keV), above the *K*-edge energy. Per dataset, 1800 images were collected while rotating continuously around the sample. For each HSP72–inhibitor complex, 15 datasets were collected from a single crystal at a flux of 3.1 × 10^10^ photons s^−1^.

### 2.5. X-ray diffraction data processing

All datasets were indexed and integrated using *DIALS* (Winter *et al.*, 2018 ▸); the BCL6 and HSP72 datasets were indexed in the *P*6_1_22 and *P*2_1_2_1_2_1_ space groups, respectively. To avoid resolution-induced effects between the different doses in a dose series for a given compound, a single high-resolution cut-off was selected for each series to ensure that reasonable quality statistics were maintained in the high-resolution data shell of the highest-dose dataset. Scaling and merging were performed using *AIMLESS* (Evans & Murshudov, 2013 ▸). Molecular replacement was performed with *PHASER* (McCoy *et al.*, 2007 ▸). PDB entry 3bim was used as an initial search model for BCL6 (Ghetu *et al.*, 2008 ▸) and 5aqy for HSP72 (Jones *et al.*, 2016 ▸). Ligand and solvent molecules were removed from the respective search models before molecular replacement. Ligand restraints were generated using *Grade* (Smart *et al.*, 2011 ▸). Iterative model building using *Coot* (Emsley *et al.*, 2010 ▸) and restrained refinement with *BUSTER* (Bricogne *et al.*, 2017 ▸) were performed against the first dataset of the dose series for each protein–ligand complex. The low-dose models were then refined against subsequent datasets without additional model building. For all ligands, the occupancies of non-halogen ligand atoms remained >0.95 and were therefore constrained to equal 1 during the final rounds of refinement, while the occupancy of ligand halogen atoms was not constrained. Data collection, processing and refinement statistics for the first sweep of the protein–ligand structures of the nine BCL6 and two HSP72 ligands can be found in Tables 1 ▸, 2 ▸ and 3 ▸. The statistics for all sweeps for all protein–ligand structures in this study can be found in the supporting information Section S2, *MSOX Crystallography Statistics Tables*.

#### 2.5.1. Quantifying the X-ray dose absorbed by protein crystals

*RADDOSE-3D* (Bury *et al.*, 2018 ▸) was used to calculate the diffraction weighted dose (DWD) for the entire X-ray exposure per crystal. For each crystal the DWD for its total X-ray exposure was divided by the number of datasets collected from the crystal to calculate the DWD per dataset.

#### 2.5.2. Generating isomorphous difference density (*F*_o__*n*_ − *F*_o1_) maps

The structure-factor amplitudes and corresponding standard deviations at each dose point were concatenated with the phases and figure of merit from refinement at the lowest dose into a single multi-column MTZ file using the CCP4 program *CAD* (Winn *et al.*, 2011 ▸). *F*_o__*n*_ − *F*_o__1_ maps, where *n* = dataset number, were then generated using *FFT* (Winn *et al.*, 2011 ▸).

#### 2.5.3. Calculating C–*X* bond radiation damage metrics

The effects of SRD on the protein–ligand structures of BCL6 (ligands **1**–**9**) and HSP72 (ligands **10**–**11**) were quantified using the *RIDL* software (Bury & Garman, 2018 ▸). *RIDL* was used to determine the amount of electron-density loss for each atom in the model by calculating their *D*_loss_ and C_α_-normalized *D*_neg_ values for each dataset after dataset 1. Detailed descriptions of *D*_loss_ and C_α_-normalized *D*_neg_ have been published by Bury & Garman (2018 ▸), but in brief, *D*_loss_ refers to the greatest density loss within the region assigned to a given atom. C_α_-normalized *D*_neg_ refers to the weighted average density loss in the region surrounding a given atom, with each voxel in the region weighted by the calculated density at that position based on the atomic model, normalized to *D*_neg_ for C_α_ atoms in the model, which are not expected to be sensitive to SRD. While the *D*_neg_ metric appears to be highly dependent on *F*_obs,*n*_ − *F*_obs,1_ map resolution, C_α_-normalized *D*_neg_ values can compensate for resolution effects up to a map resolution of about 3.5 Å (Bury & Garman, 2018 ▸). As all datasets used in this study have a resolution significantly better than this cut-off, we used the C_α_-normalized *D*_neg_ values for our analyses. For each dose series, the respective halogen *D*_loss_ and C_α_-normalized *D*_neg_ values were plotted against the X-ray dose and compared with the *D*_loss_ and C_α_-normalized *D*_neg_ values of typical radiation-sensitive protein atoms. For BCL6 we selected the Cys67 S_γ_ and Asp33 O_δ1_ atoms and for HSP72 we selected the Cys17 S_γ_ and Asp199 O_δ1_ atoms. *D*_loss_ values for all ligand halogen atoms were fitted to the one-phase exponential decay function
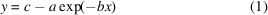
by a non-linear least-squares minimization in MATLAB (Mathworks, 2018 ▸). In this equation *y* is the fitted value, *c* is the *D*_loss_ value at which density loss plateaus, *a* and *b* fit how rapidly that density loss occurs, and *x* is the X-ray dose (the independent variable).

The coefficients of the fit were then used to estimate the amount of density loss at which *D*_loss_ plateaus (max. *D*_loss_) as well as the X-ray dose at which half of the maximum density loss (*D*_1/2_) occurs (Fig. S2).

#### 2.5.4. Calculating ligand validation metrics

For each dataset collected from the respective BCL6/ligand **2** and HSP72/ligand **10** crystals, 2*mFo*–*DFc* and *mFo*–*DFc* maps were calculated using *PHENIX.MAPS* (Pražnikar *et al.*, 2009 ▸). These maps and associated model coordinates were used as input for *EDSTATS* (Tickle, 2012 ▸) to calculate the real-space *R* factor (RSR), real-space correlation coefficient (RSCC) and average *B* factor for the respective ligands at each dose point.

#### 2.5.5. HSP72 anomalous signal analysis

Anomalous dose series collected from an HSP72/ligand **10** crystal were processed with two different protocols. In the first protocol, all individual datasets were integrated and merged separately. In the second, an increasing number of wedges were merged together; sweep 1 included data from sweep 1, and sweep *n* merged all data from datasets 1–*n*. Anomalous difference maps were then generated using *FFT* (Winn *et al.*, 2011 ▸).

## 3. Results and discussion

### 3.1. Detection of inhibitor C–*X* bond cleavage in *F*_o__*n*_ − *F*_o__1_ maps

**Figure 2:**
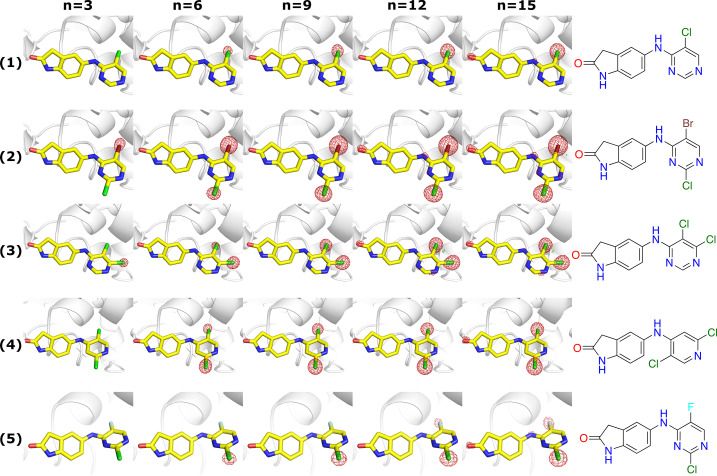
Isomorphous difference density maps for five halogenated BCL6 ligands at five dose points. The DWD is equal to *n* multiplied by the dose per dataset. Carbon atoms are shown in yellow, nitrogen atoms in blue, oxygen atoms in red, chlorine atoms in green, bromine atoms in maroon and fluorine atoms in light blue. The following five compounds are shown here: (**1**) contoured at 0.471 e Å^−3^ (5σ in *F*_o__15_ − *F*_o__1_ map), DWD = *n* × 1.51 MGy; (**2**) contoured at 0.356 e Å^−3^ (5σ in *F*_o__15_ − *F*_o__1_ map), DWD = *n* × 1.25 MGy; (**3**) contoured at 0.407 e Å^−3^ (5σ in *F*_o__15_ − *F*_o__1_ map), DWD = *n* × 1.45 MGy; (**4**) contoured at 0.273 e Å^−3^ (5σ in *F*_o__15_ − *F*_o__1_ map), DWD = *n* × 1.40 MGy; (**5**) contoured at 0.543 e Å^−3^ (5σ in *F*_o__15_ − *F*_o__1_ map), DWD = *n* × 1.29 MGy. The full 15 dataset dose series of all BCL6 inhibitors is shown in Fig. S3.

To increase our understanding of SRD in protein-bound halogenated ligands, BCL6 crystals were soaked with nine different inhibitors from the same chemical series, but with a variety of halogens (bromine, chlorine or fluorine) and a different halogen substitution pattern [ligands **1**–**9**, Fig. 1 ▸(*a*)]. From each individual inhibitor-soaked BCL6 crystal, 15 X-ray diffraction datasets were collected to monitor the changes in electron density as a function of the X-ray dose. Binding of each inhibitor was confirmed in the 2*mFo*–*DFc* and *mFo*–*DFc* electron-density maps calculated from the first dataset. For all inhibitors, analysis of the corresponding *F*_o__*n*_ − *F*_o__1_ maps, which allow the visualization of changes in electron density with dose for individual atoms (Helliwell, 1988 ▸; Bury & Garman, 2018 ▸), revealed a progressive reduction in the electron density surrounding the mean position of the ligand halogen atoms with increasing absorbed X-ray dose, regardless of the nature of the halogen substituent (Fig. 2 ▸, Fig. S3). As was previously reported for a brominated DNA/RNA hybrid (Ravelli *et al.*, 2003 ▸), the loss of electron density at the halogen positions suggested cleavage of C–*X* bonds as a result of X-ray irradiation and diffusion of the halogen atoms away from the ligand. However, in contrast to the study by Ravelli *et al.*, we did not observe an accumulation of the cleaved halogen atoms at a new position in the unit cell. We next sought to determine how quickly SRD to C–*X* bonds in protein-bound ligands occurs during the collection of X-ray diffraction data.

### 3.2. Quantifying the rate of C–*X* bond cleavage

Because *F*_o__*n*_ − *F*_o__1_ maps only provide a qualitative indicator of radiation sensitivity, we used the *RIDL* software (Bury & Garman, 2018 ▸) to quantify the rate of C–*X* bond cleavage during data collection by determining the halogen electron-density loss for each dataset after dataset 1, as calculated by their *D*_loss_ and C_α_-normalized *D*_neg_ values (descriptions of *D*_loss_ and C_α_-normalized *D*_neg_ can be found in Section 2.5.3).

**Figure 3:**
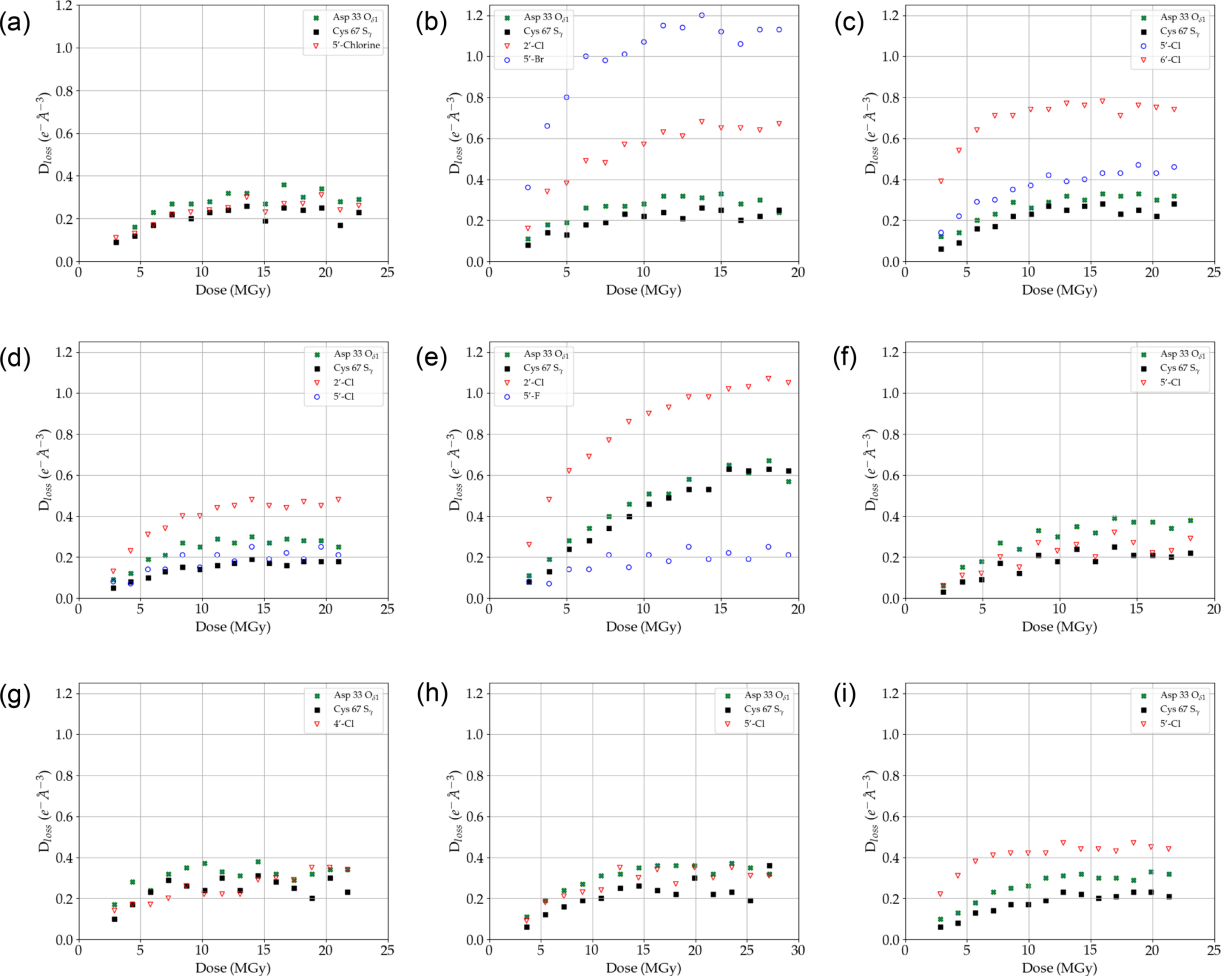
Radiation-induced density loss of BCL6 inhibitors **1**–**9** with increasing X-ray dose as compared with density loss of the BCL6 Cys67 S_γ_ and Asp33 O_δ1_ atoms. (*a*) Ligand **1**, (*b*) ligand **2**, (*c*) ligand **3**, (*d*) ligand **4**, (*e*) ligand **5**, (*f*) ligand **6**, (*g*) ligand **7**, (*h*) ligand **8**, (*i*) ligand **9**. For the corresponding C_α_-normalized *D*_neg_ curves see Fig. S4.

For all halogen atoms in the nine BCL6 inhibitors we plotted the radiation-induced density loss (*D*_loss_) and C_α_-normalized *D*_neg_ values against the X-ray dose (Fig. 3 ▸ and Fig. S4, respectively) and determined the max. *D*_loss_ and average C_α_-normalized *D*_neg_ values (Table 4 ▸). In addition, we compared the SRD sensitivity of the halogen atoms in the BCL6 ligands with the SRD observed for two representative radiation-sensitive protein atoms in the BCL6 BTB domain, the S_γ_ atom of Cys67 and the O_δ1_ atom of Asp33. Both electron-density loss of cysteine sulfur atoms and the decarboxylation of carboxylic acid containing amino acids are well documented X-ray-mediated radiation damage effects in protein structures (Burmeister, 2000 ▸; Close & Bernhard, 2019 ▸).

The trends in *D*_loss_ and C_α_-normalized *D*_neg_ curves are very similar (Fig. 3 ▸, Fig. S4), although the C_α_-normalized *D*_neg_ curves for compounds **4** and **9** are somewhat noisier than the corresponding *D*_loss_ curves. Within this set of BCL6 inhibitors, there appears to be a significant variation in sensitivity of ligand halogen atoms to SRD. For example, the fluorine atom in compound **5** is significantly less sensitive to SRD than the BCL6 Cys67 S_γ_ and Asp33 O_δ1_ atoms within the same protein–ligand structure, but for the bromine and chlorine substituents the sensitivity is generally very similar or higher than the BCL6 reference atoms (Fig. 3 ▸, Fig. S4). The bromine and chlorine *D*_1/2_ values, derived from the coefficients of the fit of the *D*_loss_ values of each ligand halogen atom to a one-phase exponential decay function, vary from 1.68 to 3.91 MGy (Table 4 ▸) and are similar to the range in the decay constant (2.0–4.5 MGy) reported for brominated DNA crystals (McGeehan *et al.*, 2007 ▸).

Our data show that the difference in sensitivity to SRD is in part due to the nature of the halogen substituent. For example, the 5′ Br in compound **2** is significantly more sensitive to SRD than the S_γ_ of Cys67 and O_δ1_ of Asp33, while the 5′ F of the matched-pair compound **5** is less sensitive than the two radiation-sensitive protein atoms (Fig. 3 ▸, Fig. S4).

In addition, our experiments suggest that the sensitivity to SRD is affected by the substitution pattern of the pyridine or pyrimidine ring in the BCL6 inhibitor scaffold. For example, the chlorine substituents in the position *ortho* to the –NH– linker have a similar sensitivity to SRD compared with the Cys67 S_γ_ and Asp33 O_δ1_ atoms, as exemplified by the *D*_loss_ and C_α_-normalized *D*_neg_ curves (Fig. 3 ▸, Fig. S4) and max. *D*_loss_ values (Table 4 ▸) for 5′ Cl atoms in compounds **1**, **6** and **8**, and the 5′ Cl position in compound **4**. This may be due to electronic effects as this position on the aromatic ring is in the *ortho* position relative to the NH which acts as an electron-donating group via its lone pair of electrons. In addition, these halogens are not adjacent to an electronegative pyridine- or pyrimidine-ring nitrogen. We therefore hypothesize that substituents that mediate an electron-withdrawing effect on the carbon to which the halogen is attached lead to an increase in sensitivity to SRD. Support for this hypothesis can be found from the 5′ Cl atom in compound **3**, which has a slightly higher sensitivity to SRD that might be due to the presence of the second, electron-withdrawing, chlorine substituent at the adjacent 6′ position in the pyrimidine ring, the only difference between compounds **1** and **3**. We cannot rule out, however, that the location of the halogen atom in the protein binding pocket could also have an effect. For example, the enclosed nature of the binding pocket around the 5′ Cl in **1**, **4**, **6** and **8** may shield this position from attack from reactive oxygen species generated through irradiation of water.

Chlorine atoms adjacent to electron-withdrawing ring nitrogens (2′ Cl atoms in compounds **2**, **4**, **5** and **9**, and the 6′ Cl in **3**) all show an increased SRD sensitivity compared with the BCL6 Cys67 S_γ_ and Asp33 O_δ1_ atoms, and also compared with other chlorine atoms present in the same molecules (Fig. 3 ▸, Table 4 ▸). An intriguing outlier is compound **7** which does not show a similar increase. This is unlikely to be due to the position in the pocket, which is similar to that of the highly sensitive 6′ Cl atom in compound **3**, supporting the theory that the difference here is probably due to the electronic properties of the ring systems. Although both the 5′ Cl atom in compound **3** and the 5′ CN group in compound **7** are electron-withdrawing groups, we hypothesize that, as CN is a conjugative withdrawing group (unlike the inductive withdrawing Cl), the CN facilitates lone-pair electron donation from the Cl into the ring, effectively strengthening the C–Cl bond. These examples serve to indicate the complex interplay of different factors which may have an impact on SRD, and we hope that this work will lead to the publication of more diverse examples, generating data that can be used to build predictive models.

We also analysed the effect of SRD by individual occupancy refinement of the halogen atoms against the first and 15th dataset collected for each BCL6 inhibitor (Table 4 ▸). For the inhibitor halogen substituents that showed extensive SRD compared with the two radiation-sensitive protein atoms (Fig. 3 ▸), such as the 2′ Cl atoms in compounds **2** and **5**, the 5′ Br and 6′ Cl atoms in compounds **2** and **3**, and the 5′ Cl in compound **9**, the max. *D*_loss_ values compared well with a large decrease in halogen occupancy, especially at the highest dose (dataset 15). In addition, for halogens with similar or lower sensitivity than the BCL6 Cys67 S_γ_ and Asp33 O_δ1_ to SRD, the more moderate max. *D*_loss_ values correlated with a much lower decrease in halogen occupancy.

**Figure 4:**
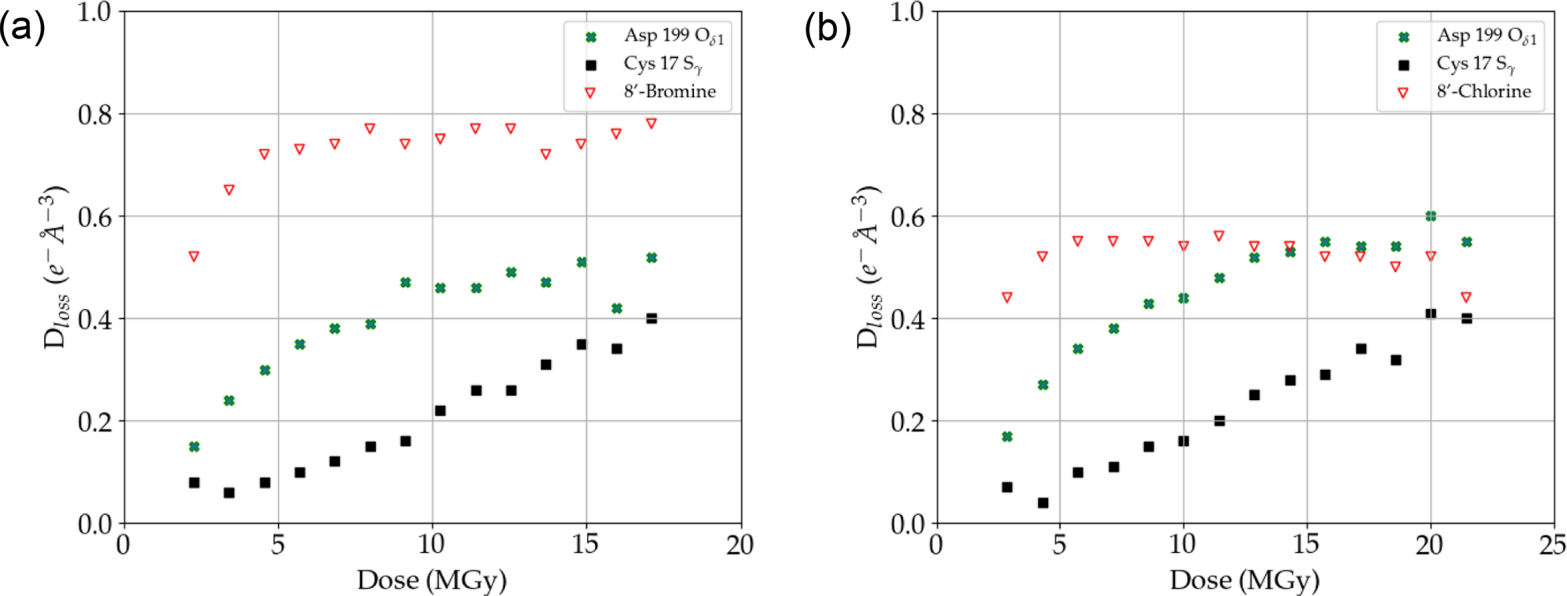
Radiation-induced density loss of HSP72 ligand **10** (*a*) and ligand **11** (*b*) with increasing X-ray dose as compared with density loss of the HSP72 Cys17 S_γ_ and Asp199 O_δ1_ atoms. For corresponding C_α_*D*_neg_ curves see Fig. S5.

**Figure 5:**
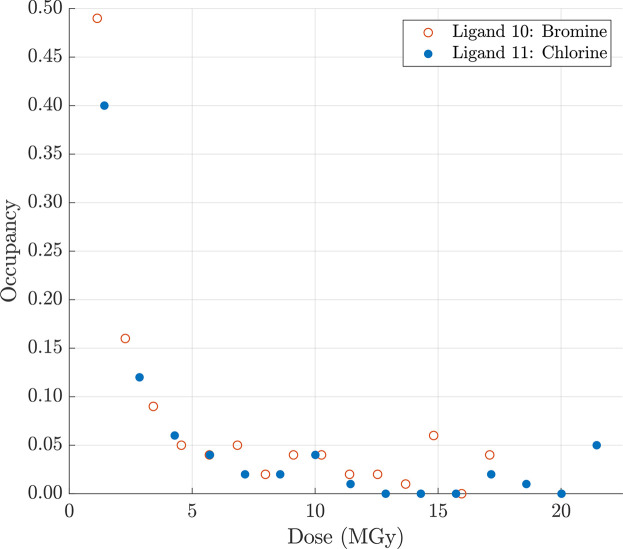
Change in occupancies of bromine and chlorine atoms of HSP72 ligands **10** (8-bromoadenosine) and **11** (8-chloroadenosine) at increasing X-ray doses. For each HSP72–ligand complex 15 datasets were collected sequentially. Each point in the graph represents a single dataset. The figure shows that the occupancies of the corresponding chlorine and bromine atoms decreased at an almost identical rate with increasing X-ray dose.

To further investigate the difference in SRD between bromine and chlorine substituents we carried out a similar MSOX data collection for crystals of the nucleotide binding domain of HSP72 soaked with an exact matched pair of bromo- (**10**) and chloro-derivatized (**11**) adenosine ligands. Similarly to several of the BCL6 inhibitors, we found that the bromine atom in 8-bromoadenosine (**10**) is more sensitive to SRD than representative radiation-sensitive HSP72 atoms, in this case the S_γ_ atom of Cys17 and the O_δ1_ atom of Asp199 (Fig. 4 ▸, Fig. S5). For 8-chloroadenosine, the result is somewhat less clear as the 8-chloro *D*_loss_ and C_α_-normalized *D*_neg_ curves both overlap with the respective curves of the Asp199 O_δ1_ atom around a dose of 15 MGy. However, it can be concluded that the ligand chlorine atom is more sensitive to SRD than the HSP72 Cys17 S_γ_ and Asp199 O_δ1_ atoms at lower doses. In addition, we found that the occupancies of the corresponding chlorine and bromine atoms decreased at an almost identical rate with increasing X-ray dose (Fig. 5 ▸). Moreover, despite use of a low-dose data collection strategy, the occupancies of the halogen atoms in ligands **10** and **11** were already reduced to less than 0.5 in dataset 1 and to less than 0.1 well before the X-ray doses at which GRD causes significantly deleterious effects on global indicators of data quality (Owen *et al.*, 2006 ▸; Warkentin *et al.*, 2014 ▸). In our view, it seems unlikely that this fast and similar rate of occupancy loss of the halogens in these matched-pair compounds is caused by the wavelength (X-ray energy) at which the data were collected. While it is true that the data collection wavelength (λ = 0.9192 Å/*E* = 13.49 keV) is above the *K*-absorption edge of both halogen atoms, where one might expect the effects of radiation damage to be most extensive (Ma *et al.*, 2024 ▸), it is much closer to the bromine *K* edge λ = 0.9202 Å/*E* = 13.47 keV) than that of chlorine (λ = 4.3939 Å/*E* = 2.82 keV) and therefore one would have expected a difference in their respective rates of occupancy loss. An alternative explanation might lie in the fact that the position of the halide on these adenosine cores is highly reactive with an electron-deficient ring system, and so we speculate that this causes the rate of reactivity to be particularly high, masking the effect of changing the halogen atom. Nevertheless, SRD of chlorinated ligands is likely to be more prevalent as ∼32% of halogenated compounds in the PDB are chlorinated while only ∼6% are brominated (Shinada *et al.*, 2019 ▸).

### 3.3. Measuring the effect of SRD on ligand validation metrics

Protein–ligand crystal structures are crucial for the rational design of new drugs, and can be particularly valuable in analysing the contributions of unusual protein–ligand interactions such as weak hydrogen bonds formed by carbon-bound halogen atoms (Kuhn *et al.*, 2019 ▸). Therefore, protein–ligand structures must be both accurate and precise. A number of valuable validation metrics have been devised (Read *et al.*, 2011 ▸; Tickle, 2012 ▸) which have resulted in a significant improvement in the quality of protein–ligand X-ray structures. However, SRD of the ligand or inhibitor is normally not taken into account in a protein–ligand structure determination and the corresponding ligand validation metrics. For example, the occupancies of all atoms in a ligand are assumed to remain constant during data collection and are typically constrained to be equal in subsequent structure refinement. This assumption is incorrect if SRD causes C–*X* bond cleavage, which could have a negative impact on the fit of the ligand to the experimental data.

**Figure 6:**
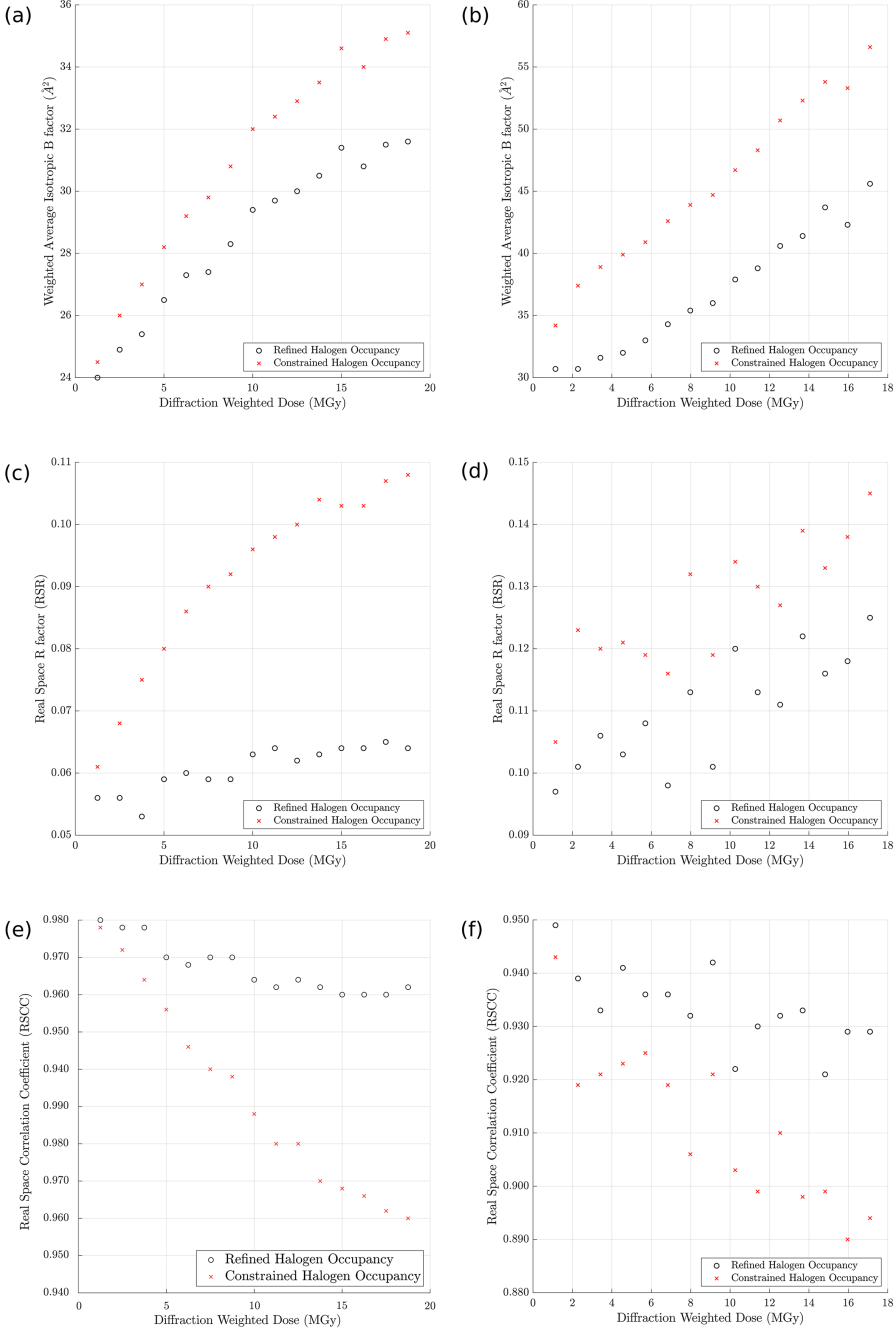
The effect of constrained and individual occupancy refinement of ligand halogen atoms on ligand validation metrics. Panels (*a*), (*c*) and (*e*), respectively, show the weighted isotropic *B* factor, the RSR and the RSCC plotted as a function of DWD for the BCL6 ligand **2** with constrained (red crosses) and individual (grey open circles) occupancy refinement. Panels (*b*), (*d*) and (*f*) show the same analysis for the HSP72 ligand **10**.

For two of our ligand-bound structures, BCL6 complexed with ligand **2** and HSP72 in complex with ligand **10**, we compared the effect of SRD on ligand validation metrics with and without individual refinement of the occupancies of the ligand halogen atoms. For both ligands the *B* factors of all ligand atoms increased with X-ray dose [Figs. 6 ▸(*a*), 6 ▸(*b*)], regardless of whether or not the halogen occupancies were individually refined. However, for both ligands the increase in the average *B* factor was lower with individual halogen occupancy refinement than without, with HSP72 ligand **10** showing the largest effect.

We also analysed the effect of occupancy refinement of individual ligand halogen atoms on the RSR and RSCC, two metrics used to quantify the fit of a ligand to its electron density. For both ligands the RSR increased with increasing X-ray dose when the occupancies of the halogen atoms were constrained to 1.0 [Figs. 6 ▸(*c*), 6 ▸(*d*)]. For the HSP72 ligand **10** the RSR also increased with individual refinement of the halogen occupancy, but slightly less than with constrained refinement [Fig. 6 ▸(*d*)]. However, the RSR of BCL6 ligand **2** remained stable when the occupancies of the halogen atoms were refined individually [Fig. 6 ▸(*c*)]. In a similar trend, refinement of BCL6 ligand **2** and HSP72 ligand **10** with their halogen occupancies constrained to 1.0 showed a reduction in their respective RSCC values from 0.99 to 0.95 and 0.95 to 0.89 with increasing X-ray dose. However, when the halogen occupancies were refined individually their RSCC values showed smaller reductions to 0.98 and 0.92, respectively [Figs. 6 ▸(*e*), 6 ▸(*f*)].

Thus, the increase in average *B* factor and RSR with increasing X-ray dose, combined with the reduction in RSCC values observed for both the BCL6 and HSP72 ligands, demonstrates that X-ray-induced C–*X* bond cleavage can cause a dose-dependent deterioration in the fit of ligand coordinates in protein–ligand crystal structures, which can be easily mitigated by individual refinement of the ligand halogen atoms. One could argue that the effect of SRD on these three indicators is relatively small, but it is important to note that for smaller halogenated ligands and fragments, in which the halogen atoms account for a larger fraction of the ligand, this effect may be more pronounced. For example, a fragment library typically contains fragments with a molecular weight between 120 and 250 Da (Rees *et al.*, 2004 ▸) and thus a bromine atom in a halogenated fragment can represent approximately 67–32% of the fragment’s total scattering mass. In addition, fragment halogen atoms are often involved in protein–ligand interactions via halogen bonds and hydrophobic interactions. Therefore, for crystallographic fragment screening experiments where X-ray-mediated C–*X* cleavage cannot always be avoided, individual occupancy refinement of the halogen atom will mitigate the effect of SRD on the average *B* factor, RSCC and RSR indicators, thus helping researchers to correctly model the experimental X-ray data and interpret these interactions.

### 3.4. Change in anomalous signal from brominated ligands with X-ray dose

To unambiguously determine the position of a bound brominated ligand, or to obtain starting phases in difficult protein–ligand structure determinations, the anomalous signal of the bromine substituent can be used by collecting the X-ray data at the bromine *K* edge (Tiefenbrunn *et al.*, 2014 ▸; Wood *et al.*, 2019 ▸; Beck *et al.*, 2010 ▸). Anomalous scattering typically contributes to only a few per cent of the total scattering from a protein crystal and is detected by measuring the very small differences between the intensities of Friedel pair reflections (Dauter, 2017 ▸). To accurately measure the anomalous signal, the collection of high-multiplicity X-ray data is required. This averages random errors across several observations which improves the accuracy of all measured intensities, thus also increasing the accuracy of determining the differences in Friedel pair intensities (Karplus & Diederichs, 2015 ▸). However, a study on sulfur-SAD(single-wavelength anomalous dispersion) phasing of thaumatin using high-multiplicity X-ray data identified a point of diminishing returns, *i.e.* a dose at which the gains in data quality due to increased multiplicity were outweighed by the losses in signal to noise due to radiation damage (Storm *et al.*, 2017 ▸).

**Figure 7:**
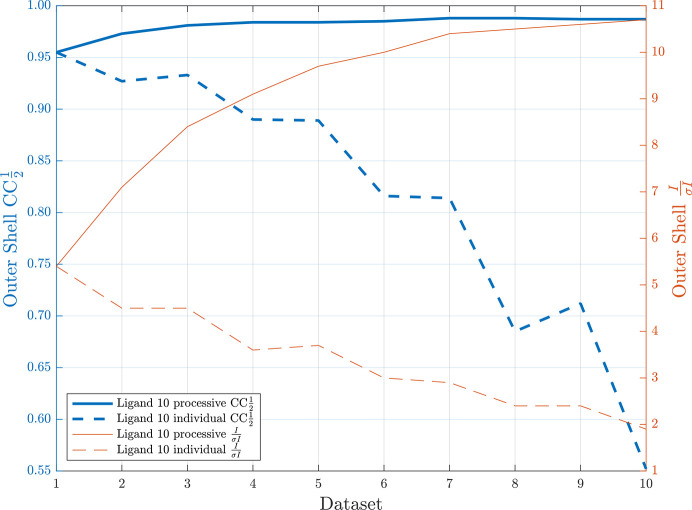
Highest-shell (1.97–1.92 Å) CC_1/2_ and *I*/σ(*I*) values as a function of number of sequential datasets (increasing X-ray dose) collected from a single HSP72 crystal in complex with ligand **10**. ‘Individual’ processing (dashed lines) only included data from a single dataset (*n*) while ‘processive’ processing (solid lines) combined all data up to and including the dataset (1 − *n*).

We investigated the effect of radiation damage with increasing X-ray dose on the bromine anomalous signal by collecting ten datasets from a single HSP72 crystal in complex with the brominated ligand **10**. We observed a reduction in the signal-to-noise [*I*/σ(*I*)] ratio and CC_1/2_ values in the high-resolution shell with increasing number (*n*) of separately processed datasets, indicating an increasing effect of GRD with increasing X-ray dose (Fig. 7 ▸). By contrast, upon sequential merging of all ten datasets, the high-resolution values of *I*/σ(*I*) increased as more data were included. In addition, the highest-shell CC_1/2_ values increased from 0.95 to 0.98 upon merging the first four datasets and remained stable as more and higher-dose datasets were included (Fig. 7 ▸). This highlights the potential to improve data quality by collecting highly redundant datasets despite the effects of GRD, which is consistent with several published examples of successful native-SAD phasing experiments from highly redundant data (Olieric *et al.*, 2016 ▸; Dauter & Adamiak, 2001 ▸; El Omari *et al.*, 2014 ▸).

**Figure 8:**
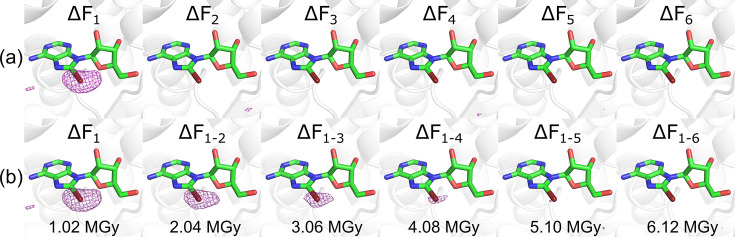
Anomalous difference density maps for the brominated HSP72 ligand **10**. (*a*) Maps calculated after individual processing for wedge *n* only, after exposure to increasing X-ray doses, 1.02 MGy per wedge. (*b*) Maps calculated after processive processing including data from an increasing number of wedges. The anomalous difference maps (purple mesh) are contoured at 0.101 e Å^−3^ (5σ in Δ*F*_1_ map).

However, anomalous difference electron-density maps show that, although a significant anomalous signal from the bromine atom of ligand **10** is present in the first dataset [Fig. 8 ▸(*a*)], the anomalous signal is lost rapidly with increasing X-ray dose [Fig. 8 ▸(*a*)], even when the datasets are sequentially merged [Fig. 8 ▸(*b*)]. In this dose series, the point of diminishing returns where the anomalous signal has completely deteriorated lies at a dose of approximately 4.08 MGy [Fig. 8 ▸(*b*)]. Although this is dependent on the size of the crystal, it is within the range (1.6–4.6 MGy) observed in thaumatin sulfur-SAD experiments (Storm *et al.*, 2017 ▸). This demonstrates that, for experiments aimed at using the anomalous signal in brominated ligands for phasing and/or unambiguous confirmation of binding, a data collection strategy that carefully balances data redundancy with minimizing the effects of SRD on the anomalous bromine signal should be adopted.

This is particularly important in the context of crystallographic screening of brominated fragment libraries specifically designed to enable ligand identification using anomalous scattering (Tiefenbrunn *et al.*, 2014 ▸; Bauman *et al.*, 2016 ▸; Wood *et al.*, 2019 ▸; Ma *et al.*, 2024 ▸). In addition, the halogen anomalous signal of brominated fragments can be used to overcome challenges in the unambiguous fragment fitting, because many fragments are often quasi-symmetric due to their low mol­ecular weight and limited functionality (Wood *et al.*, 2019 ▸; Ma *et al.*, 2024 ▸). However, in general, fragments bind weakly and often with low occupancy, which may reduce an already small anomalous signal. Additional X-ray-mediated C–*X* cleavage will result in a further deterioration of the anomalous signal, especially if the data are collected at a wavelength just above the bromine *K*-absorption edge where SRD effects might be most pronounced, as recently reported for iodinated fragments (Ma *et al.*, 2024 ▸). Therefore, to facilitate the identification and unambiguous placement of brominated fragment hits through identification of the bromine position(s), it is advisable to collect an initial anomalous dataset at low dose, followed by a second dataset at higher dose to capture higher-resolution data for structure refinement.

## 4. Concluding remarks

Crystallographic data collection strategies are a balance between collecting accurate data with high completeness and redundancy to as high a resolution as possible and minimizing the effects of both GRD and SRD (Dauter, 2010 ▸). Recent developments in data collection algorithms have made data collection protocols aimed at reducing the total diffraction weighted dose that the crystal receives, such as helical scans (Flot *et al.*, 2010 ▸) and multi-crystal data collection (Berglund *et al.*, 2002 ▸; Hough & Owen, 2021 ▸), increasingly compatible with high-throughput crystallographic ligand screening at third-generation synchrotrons; such approaches could be used to mitigate the effects of X-ray-mediated C–*X* cleavage in protein-bound halogenated ligands (Svensson *et al.*, 2018 ▸). However, currently the data collection software typically does so by aiming to minimize GRD, rather than the more rapid SRD (Bourenkov & Popov, 2010 ▸).

In this study, we have demonstrated the occurrence of significant SRD by X-ray-induced radiolysis of inhibitor/ligand C–*X* bonds during data collection from protein crystals of BCL6 or HSP72 complexed with halogen-containing inhibitors or ligands. We have shown that there is a large variation in inhibitor C–*X* bond sensitivity to specific X-ray-induced radiolysis, which depends on the chemical structure of the ligand and the type of halogen substituent. We also confirmed that X-ray-induced cleavage of inhibitor C–*X* bonds takes place at doses far below the dose at which GRD occurs. By analysing the effect of different refinement strategies on the average *B* factor of ligand halogen atoms and the ligand RSR and RSCC, we showed that, when C–*X* bond cleavage is observed, the agreement between the electron-density maps and the model can be improved by refining the occupancies of halogen atoms separately from the rest of the ligand.

Nevertheless, the analysis of the effects of C–Br cleavage on the anomalous signal of HSP72 ligand **10** revealed a deterioration of the anomalous signal with increasing X-ray dose that cannot be easily avoided. It has been suggested that SRD to halogenated ligands in protein crystals can be exploited for experimental phasing (Beck *et al.*, 2010 ▸) using the radiation-induced phasing (RIP) method previously applied to crystals of brominated nucleotides (Ravelli *et al.*, 2003 ▸). However, for the vast majority of structure-based and fragment-based drug discovery campaigns, the crystal structure of the drug target is already available at the start of the campaign. Therefore, this method will only be valuable for the very few cases in which the protein structure is not available and cannot be easily determined with traditional phasing methods.

A way to locate the position of halogen atoms in bound halogenated ligands without making use of the anomalous signal is via the visualization of C–*X* cleavage in *F*_o__*n*_ − *F*_o__1_ maps (Gigant *et al.*, 2005 ▸). Alternatively, visualization of SRD can be achieved by calculation maps using the *F*(early) and *F*(late) map coefficients from the early and late parts of a dataset using Global Phasing’s *autoPROC* (Vonrhein *et al.*, 2011 ▸). While this is an intelligent exploitation of SRD, which can also help to unambiguously define the binding orientation of the ligand (Gigant *et al.*, 2005 ▸), the same can be achieved in a very accurate manner using the anomalous signal of the ligand halogen atom(s) (Ma *et al.*, 2024 ▸). However, as we have shown in this study, the anomalous signal of ligand halogen atoms can be severely affected by X-ray-mediated C–*X* bond cleavage, which is why we recommend the use of low-dose data collection strategies and individual refinement of ligand halogen atoms to minimize the effects of SRD on the final model.

## Supplementary Material

Supporting information file with figures, chemical synthesis of the compounds, tables with full X-ray statistics. DOI: 10.1107/S1600576724010549/jo5108sup1.pdf

PDB reference: BCL6 ligand 1, 7gud

PDB reference: BCL6 ligand 2, 7gus

PDB reference: BCL6 ligand 3, 7gv7

PDB reference: BCL6 ligand 4, 7gvm

PDB reference: BCL6 ligand 5, 7gw1

PDB reference: BCL6 ligand 6, 7gwg

PDB reference: BCL6 ligand 7, 7gwv

PDB reference: BCL6 ligand 8, 7gxa

PDB reference: BCL6 ligand 9, 7gxp

PDB reference: HSP72 ligand 10, 7gy4

PDB reference: HSP72 ligand 111, 7gyj

## Figures and Tables

**Table 1:** MSOX crystallography statistics tables BCL6 ligands **1**–**5**, sweep 1 For statistics for all sweeps see Table S2.

Ligand, PDB code, dose (MGy)†	**1**, 7gud, 1.51	**2**, 7gus, 2.27	**3**, 7gv7, 1.45	**4**, 7gvm, 1.40	**5**, 7gw1, 1.29
Space group	*P*6_1_22	*P*6_1_22	*P*6_1_22	*P*6_1_22	*P*6_1_22
Unit cell (*a* = *b*, *c*) (Å)	67.51, 165.98	67.74, 166.68	67.58, 166.30	67.74, 166.87	67.49, 166.30
Unit cell (α = β, γ) (°)	90.00, 120.00	90.00, 120.00	90.00, 120.00	90.00, 120.00	90.00, 120.00
Beamline	DLS I24	DLS I24	DLS I24	DLS I24	DLS I24
Wavelength (Å)	0.9686	0.9686	0.9686	0.9686	0.9688
Resolution (Å)‡	33.84–1.80	33.97–1.75	33.89–1.85	34.00–1.90	33.88–1.75
	(1.84–1.80)	(1.78–1.75)	(1.89–1.85)	(1.94–1.90)	(1.78–1.75)
Unique reflections‡	21648 (1247)	23750 (1284)	19809 (1219)	18736 (1180)	23337 (1268)
Multiplicity‡	9.1 (9.3)	9.1 (9.3)	9.2 (9.3)	9.0 (9.4)	9.2 (9.3)
*R*_p.i.m._ (%)‡	2.7 (16.7)	2.6 (20.0)	4.5 (14.4)	2.0 (14.8)	2.7 (8.8)
*R*_meas_ (%)‡	8.1 (51.7)	7.8 (61.9)	13.8 (45.0)	6.0 (45.9)	8.5 (26.9)
CC_1/2_‡§	0.998 (0.946)	0.998 (0.943)	0.990 (0.944)	1.000 (0.941)	0.997 (0.97)
*I*/σ(*I*)‡	14.5 (4.2)	14.4 (3.4)	10.4 (4.7)	18.5 (4.4)	15.8 (6.7)
Completeness (%)‡	100.0 (100.0)	100.0 (100.0)	99.5 (100.0)	100.0 (100.0)	99.7 (100.0)
Wilson *B* (Å^2^)	18.9	20.2	20.2	26.2	17.9

*Refinement*					
*R*_work_/*R*_free_	18.03/19.85	18.46/21.54	17.80/20.04	19.62/22.33	17.00/19.12
No. atoms					
Protein	1176	1173	1139	1078	1190
Ligand/ion	26	27	27	27	27
Water	181	185	161	138	152
Ramachandran (No., %)					
Allowed	121 (97.58%)	119 (95.97%)	120 (96.77%)	120 (98.36%)	119 (95.97%)
Generally allowed	3 (2.42%)	5 (4.03%)	4 (3.23%)	2 (1.64%)	5 (4.03%)
Disallowed	0 (0.00%)	0 (0.00%)	0 (0.00%)	0 (0.00%)	0 (0.00%)
*B* factors					
Protein (Å^2^)	27.52	28.80	27.01	36.12	25.02
Ligand/ion (Å^2^)	25.39	26.30	27.72	32.80	24.93
Water (Å^2^)	45.12	45.69	43.40	49.89	42.74

*RMS deviations*					
Bond lengths (Å)	0.01	0.01	0.01	0.01	0.01
Bond angles (°)	0.90	0.87	0.88	0.93	0.89

† DWD (Zeldin *et al.*, 2013 ▸).

‡ Values in parentheses are for the highest-resolution shell.

§ Half-dataset correlation coefficient (Karplus & Diederichs, 2012 ▸).

**Table 2:** MSOX crystallography statistics tables BCL6 ligands **6**–**9**, sweep 1 For statistics for all sweeps see Table S2.

Ligand, PDB code, dose (MGy)†	**6**, 7gwg, 1.23	**7**, 7gwv, 1.45	**8**, 7gxa, 1.81	**9**, 7gxp, 1.42
Space group	*P*6_1_22	*P*6_1_22	*P*6_1_22	*P*6_1_22
Unit cell (*a* = *b*, *c*) (Å)	67.74, 166.87	67.35, 165.67	67.69, 166.53	67.58, 165.83
Unit cell (α = β, γ) (°)	90.00, 120.00	90.00, 120.00	90.00, 120.00	90.00, 120.00
Beamline	DLS I24	DLS I24	DLS I24	DLS I24
Wavelength (Å)	0.9686	0.9686	0.9686	0.9686
Resolution (Å)‡	34.00–1.90	33.77–11.70	33.94–11.95	33.83–11.85
	(1.94–11.90)	(1.73–11.70)	(2.00–11.95)	(1.89–11.85)
Unique reflections‡	18736 (1180)	25384 (1316)	17317 (1187)	18223 (1144)
Multiplicity‡	9.0 (9.4)	9.1 (9.3)	9.0 (9.3)	9.9 (9.8)
*R*_p.i.m._ (%)‡	2.0 (14.8)	2.0 (23.5)	2.2 (10.5)	2.3 (14.9)
*R*_meas_ (%)‡	6.0 (45.9)	6.0 (73.5)	6.6 (32.4)	7.6 (47.9)
CC_1/2_‡§	1.000 (0.941)	0.999 (0.935)	0.999 (0.970)	0.999 (0.923)
*I*/σ(*I*)‡	18.5 (4.4)	21.8 (3.4)	18.8 (6.3)	15.9 (4.4)
Completeness (%)‡	100.0 (100.0)	100.0 (100.0)	100.0 (100.0)	92.8 (95.2)
Wilson *B* (Å^2^)	26.2	14.4	20.6	19.3

*Refinement*				
*R*_work_/*R*_free_	19.62/22.33	18.82/20.97	17.86/20.53	17.92/21.34
No. atoms				
Protein	1078	1126	1092	1118
Ligand/ion	27	29	29	31
Water	138	208	168	170
Ramachandran (No. %)				
Allowed	120 (98.36%)	119 (95.97%)	120 (97.56%)	119 (96.75%)
Generally allowed	2 (1.64%)	5 (4.03%)	3 (2.44%)	4 (3.25%)
Disallowed	0 (0.00%)	0 (0.00%)	0 (0.00%)	0 (0.00%)
*B* factors				
Protein (Å^2^)	36.12	21.67	28.16	28.24
Ligand/ion (Å^2^)	32.80	23.94	28.42	29.21
Water (Å^2^)	49.89	39.31	45.70	44.23

*RMS deviations*				
Bond lengths (Å)	0.01	0.01	0.01	0.01
Bond angles (°)	0.93	0.89	0.93	0.94

† DWD (Zeldin *et al.*, 2013 ▸).

‡ Values in parentheses are for the highest-resolution shell.

§ Half-dataset correlation coefficient (Karplus & Diederichs, 2012 ▸).

**Table 3:** MSOX crystallography statistics tables HSP72 ligands **10**–**11**, sweep 1 For statistics for all sweeps see Table S2.

Ligand, PDB code, dose (MGy)†	**10**, 7gy4, 1.14	**11**, 7gyj, 1.43
Space group	P2_1_2_1_2_1_	P2_1_2_1_2_1_
Unit cell (*a*, *b*, *c*) (Å)	47.74, 89.31, 96.70	52.07, 82.11, 93.31
Unit cell (α = β = γ) (°)	90.00	90.00
Beamline	DLS I24	DLS I24
Wavelength (Å)	0.9192	0.9192
Resolution (Å)‡	38.60–1.92	39.78–2.15
	(1.97–1.92)	(2.22–2.15)
Unique reflections‡	32381 (2128)	22462 (1913)
Multiplicity‡	6.3 (6.4)	6.3 (6.3)
*R*_p.i.m._ (%)‡	4.0 (12.5)	2.4 (7.8)
*R*_meas_ (%)‡	10.0 (31.9)	6.0 (19.8)
CC_1/2_‡§	0.993 (0.955)	0.999 (0.983)
*I*/σ(*I*)‡	11.4 (5.4)	18.4 (8.0)
Completeness (%)‡	100.0 (100.0)	100.0 (100.0)
Wilson *B* (Å^2^)	18.9	22.6

*Refinement*		
*R*_work_/*R*_free_	16.93/21.47	16.13/20.39
No. atoms		
Protein	3031	2983
Ligand/ion	35	92
Water	492	319
Ramachandran (No., %)		
Allowed	382 (98.45%)	379 (98.96%)
Generally allowed	6 (1.55%)	4 (1.04%)
Disallowed	0 (0.00%)	0 (0.00
*B* factors		
Protein (Å^2^)	27.30	30.80
Ligand/ion (Å^2^)	36.56	44.48
Water (Å^2^)	38.08	40.72

*RMS deviations*		
Bond lengths (Å)	0.01	0.01
Bond angles (°)	0.99	0.99

† DWD (Zeldin *et al.*, 2013 ▸).

‡ Values in parentheses are for the highest-resolution shell.

§ Half-dataset correlation coefficient (Karplus & Diederichs, 2012 ▸).

**Table 4:** The maximum *D*_loss_ and *D*_1/2_ calculated from the coefficients of the exponential fit and *R*^2^ values for fit of one-phase exponential to *RIDL* data

Ligand	Halogen atom	*R* ^2^	Max. *D*_loss_ (e Å^−3^)	*D*_1/2_ (MGy)	Occupancy *N* = 1	Occupancy *N* = 15	δ Occupancy
**1**	5′ Cl	0.86	0.28	3.17	0.99	0.99	0.00
**2**	2′ Cl	0.98	0.67	2.95	0.81	0.40	−0.41
	5′ Br	0.97	1.14	1.88	0.96	0.62	−0.34
**3**	5′ Cl	0.97	0.46	3.91	0.99	0.81	−0.18
	6′ Cl	0.97	0.75	1.68	0.56	0.10	−0.46
**4**	2′ Cl	0.98	0.47	2.70	0.82	0.44	−0.38
	5′ Cl	0.78	0.23	3.76	0.94	0.84	−0.10
**5**	2′ Cl	1.00	1.09	3.61	0.72	0.21	−0.51
	5′ F	–	–	N.D.†	–	–	–
**6**	5′ Cl	0.77	0.27	3.01	0.97	0.89	−0.08
**7**	4′ Cl	–	–	N.D.†	0.91	0.72	−0.19
**8**	5′ Cl	0.86	0.33	3.34	0.90	0.75	−0.15
**9**	5′ Cl	0.96	0.45	1.77	0.45	0.16	−0.29

† *D*_loss_ did not plateau within the dose range of the experiment. It is therefore not possible to calculate *D*_1/2_ for these samples. Occupancy of ligand **5** 5′ F did not change with dose and was constrained to 1.

## Data Availability

Atomic coordinates and structure factors for the crystal structures of BCL6 and HSP72 described in this article can be accessed using PDB codes listed in the tables of crystallographic statistics in Table S2.
