# Supplementary material for: Specific radiation damage to halogenated inhibitors and ligands in protein–ligand crystal structures
Source: J Appl Crystallogr. 2024 Nov 26;57(Pt 6):1951–65. doi: 10.1107/S1600576724010549 (PMC11611281; doi:10.1107/S1600576724010549)
Supplement: Supplementary file 1 [file j-57-01951-sup1.pdf]

JOURNAL OF APPLIED CRYSTALLOGRAPHY

Volume 57 (2024)

Supporting information for article:

Specific radiation damage to halogenated inhibitors and ligands in protein–ligand crystal structures

Matthew J. Rodrigues, Marc Cabry, Gavin Collie, Michael Carter, Craig McAndrew, Robin L. Owen, Benjamin R. Bellenie, Yann-Vaï Le Bihan and Rob L. M. van Montfort

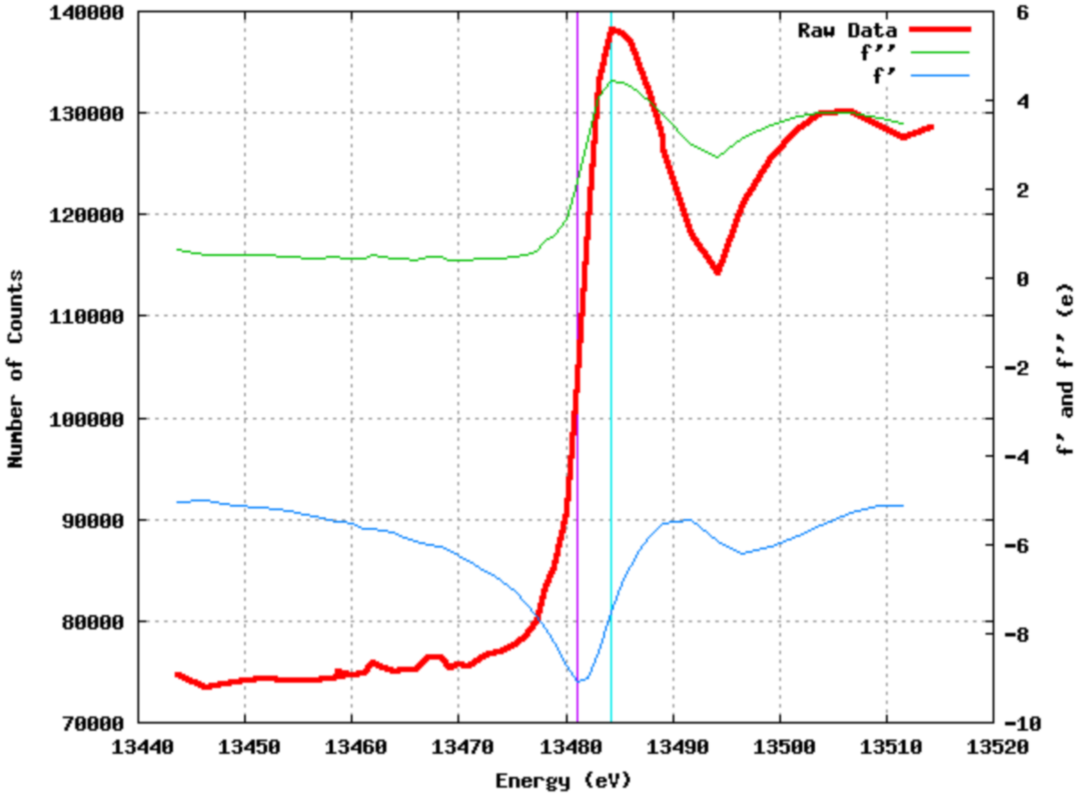

Figure S1 XANES spectrum of a HSP72 crystal in complex with ligand **10**. Anomalous scattering factors *f'* and *f''* were calculated using CHOOCH (Evans & Pettifer, 2001).

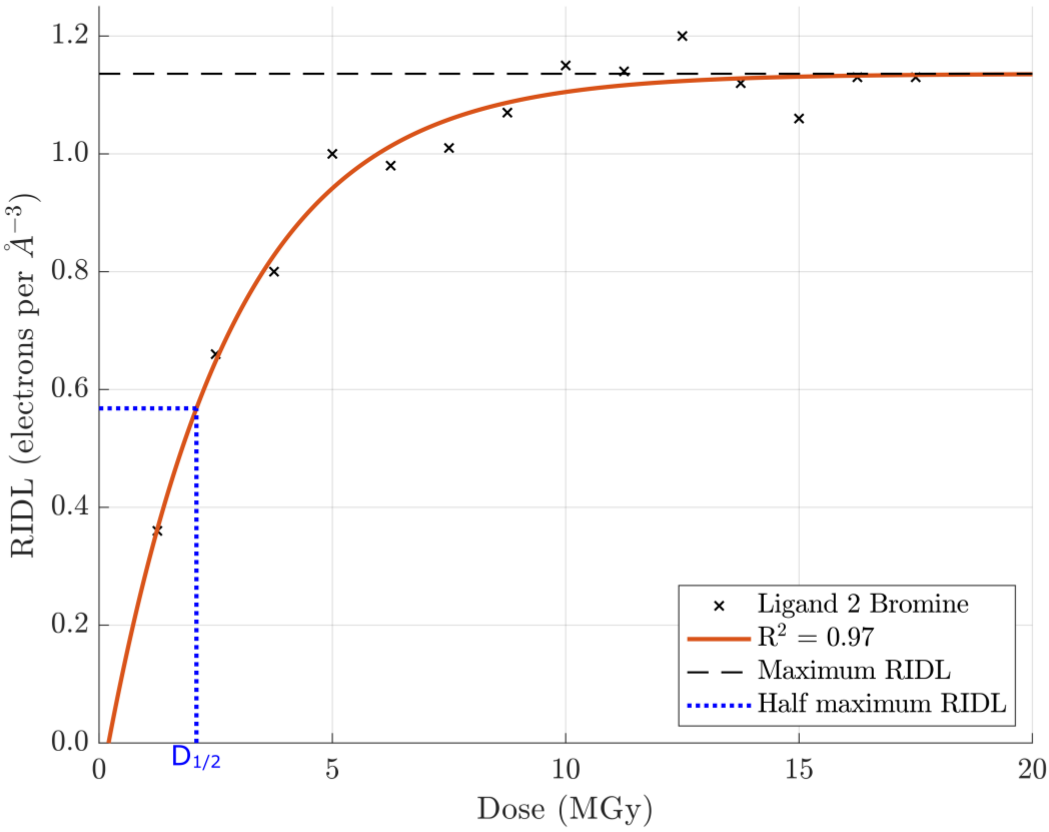

Figure S2 Plot of Radiation Induced Density Loss for the bromine atom of ligand **2** with a one-phase exponential decay curve fitted to the data. Fitting of the exponential to the curve allows the amount of RIDL at which density loss plateaus be estimated. The X-ray dose between the dose at which Fo_1 is collected and the dose at which half of the maximum RIDL occurs is defined as $D_{1/2}$.

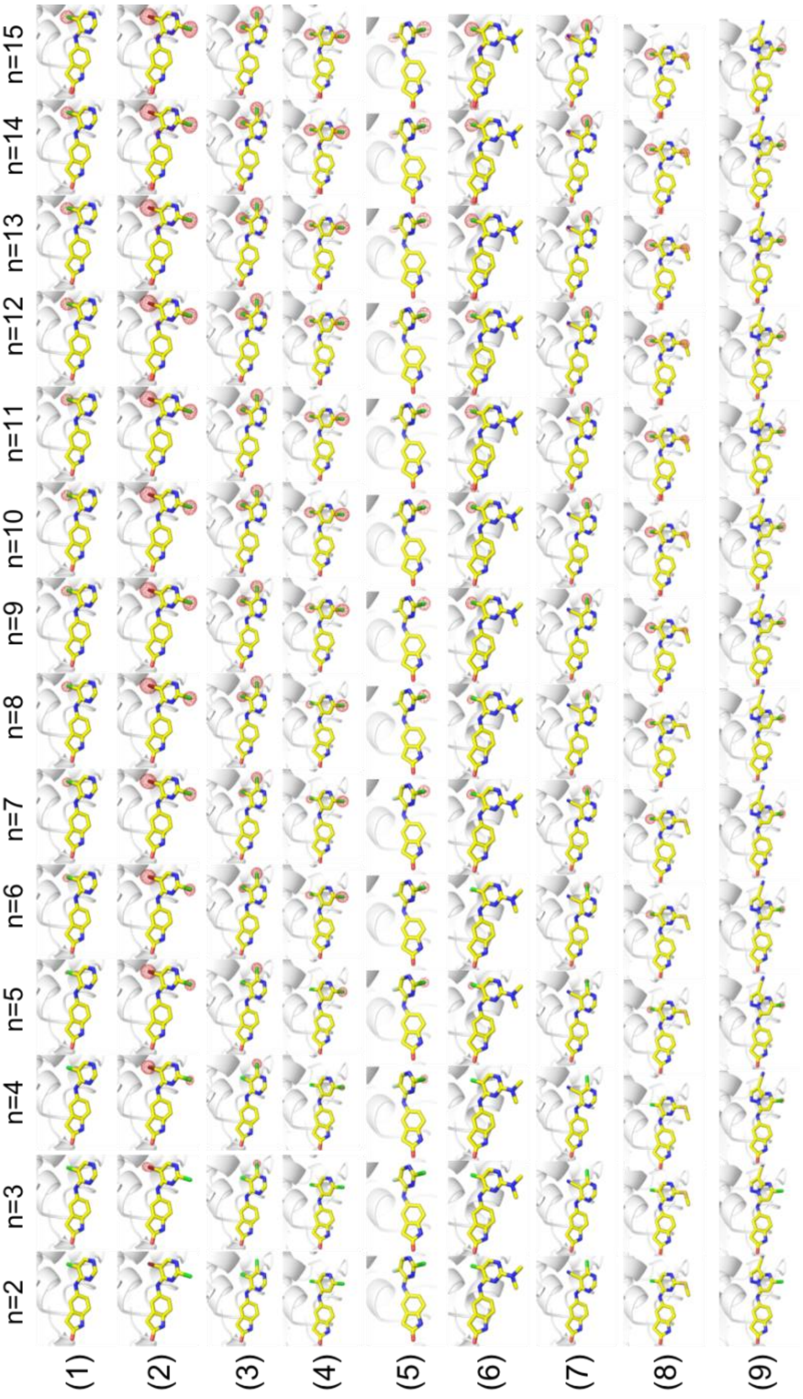
n=2
n=3
n=4
n=5
n=6
n=7
n=8
n=9
n=10
n=11
n=12
n=13
n=14
n=15
(1)
(2)
(3)
(4)
(5)
(6)
(7)
(8)
(9)

Figure S3 Isomorphous difference density maps for nine halogenated BCL6 ligands at fifteen dose points. The diffraction weighted dose (DWD) is equal to *n* multiplied by the dose per dataset. Carbon atoms are shown yellow, nitrogen atoms in blue, oxygen atoms in red, chlorine atoms in green, bromine atoms in maroon, and fluorine atoms in light blue. The following ligand are shown:
(**1**) contoured at 0.471 e^{-} $Å^{-3}$ (5 σ in $Fo_{15} – Fo_{1}$ map), DWD = *n* x 1.51 MGy;
(**2**) contoured at 0.356 e^{-} $Å^{-3}$ (5 σ in $Fo_{15} – Fo_{1}$ map), DWD = *n* x 1.25 MGy;
(**3**) contoured at 0.407 e^{-} $Å^{-3}$ (5 σ in $Fo_{15} – Fo_{1}$ map), DWD = *n* x 1.45 MGy;
(**4**) contoured at 0.273 e^{-} $Å^{-3}$ (5 σ in $Fo_{15} – Fo_{1}$ map), DWD = *n* x 1.40 MGy;
(**5**) contoured at 0.543 e^{-} $Å^{-3}$ (5 σ in $Fo_{15} – Fo_{1}$ map), DWD = *n* x 1.29 MGy;
(**6**) contoured at 0.393 e^{-} $Å^{-3}$ (5 σ in $Fo_{15} – Fo_{1}$ map), DWD = *n* x 1.23 MGy;
(**7**) contoured at 0.582 e^{-} $Å^{-3}$ (5 σ in $Fo_{15} – Fo_{1}$ map), DWD = *n* x 1.45 MGy;
(**8**) contoured at 0.446 e^{-} $Å^{-3}$ (5 σ in $Fo_{15} – Fo_{1}$ map), DWD = *n* x 1.81 MGy;
(**9**) contoured at 0.383 e^{-} $Å^{-3}$ (5 σ in $Fo_{15} – Fo_{1}$ map), DWD = *n* x 1.42 MGy.

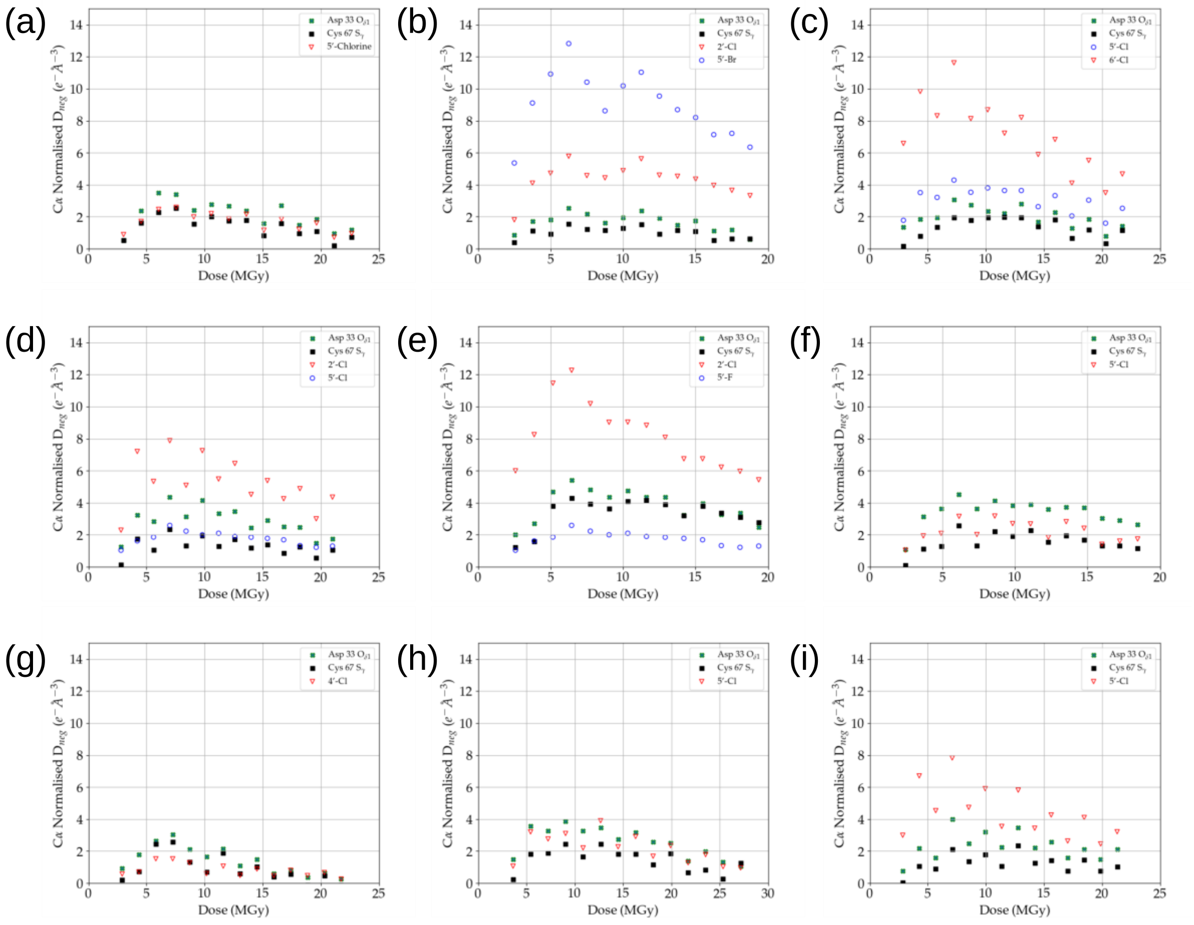

Figure S4 Radiation Induced Density Loss of BCL6 inhibitors. C_α normalised D_{neg} curves of BCL6 inhibitors **1-9** with increasing X-ray dose as compared to density loss of the BCL6 Cys67 S_γ and Asp 33 $O_{\delta 1}$ atoms. (a) Ligand **1**, (b) ligand **2**, (c) ligand **3**, (d) ligand **4**, (e) ligand **5**, (f) ligand **6**, (g) ligand **7**, (h) ligand **8**, (i) ligand **9**.

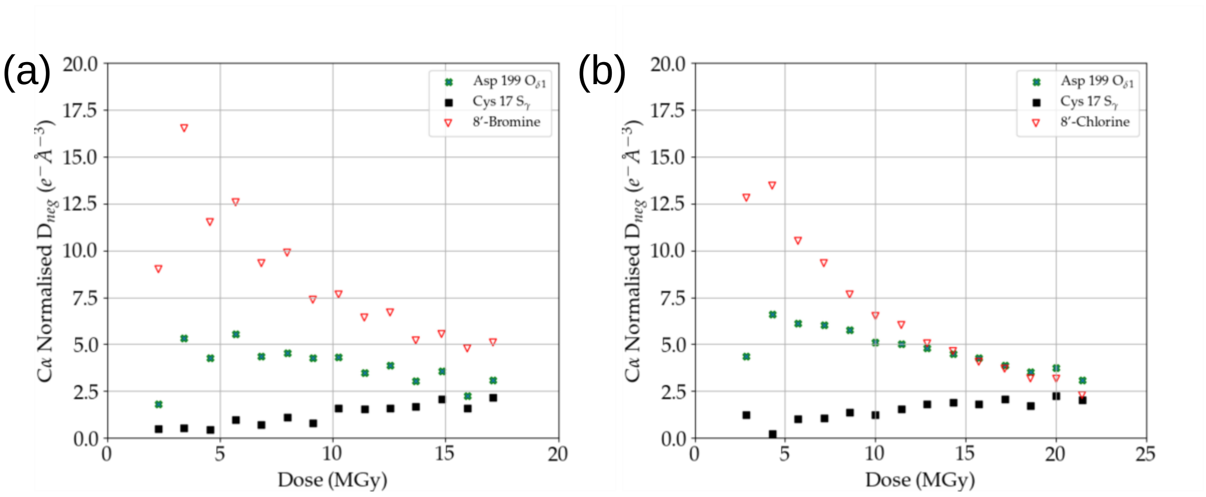

Figure S5 Radiation Induced Density Loss of HSP72 ligands. (a) C_α normalised D_{neg} curves of HSP72 ligand **10** (a) and ligand **11** (b) with increasing X-ray dose as compared to density loss of the HSP72 Cys17 S_γ and Asp199 $O_{\delta 1}$ atoms.

S1. Synthesis of BCL6 ligands

S1.1. Ligand 1: 5-((5-Chloropyrimidin-4-yl)amino)indolin-2-one

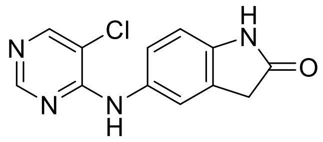

To a mixture of 5-amino-2-oxindole (30 mg, 0.2 mmol) and 4,5-dichloropyrimidine (30 mg, 0.2 mmol) in N-methylpyrrolidinone (1.7 mL) was added DIPEA (44 uL, 0.25 mmol), and the resulting mixture was heated at 120 °C under microwave irradiation for 30 min. The resulting mixture was purified by HPLC (2 injections; ACE 5 C18-PFP 250 x 21.2 mm column; 15 min gradient of 90:10 to 0:100 water:methanol (both modified with 0.1 % formic acid); flow rate 20 $mLmin^{-1}$; Agilent 6120 MS-Prep LC) affording the title compound (28 mg, 53%) as a beige solid. ^{1}H NMR (500 MHz, DMSO-d_6) δ 10.37 (s, 1H), 9.02 (s, 1H), 8.42 (s, 1 H), 8.39 (s, 1H), 7.44 (s, 1H), 7.31 (dd, J = 8.3, 1.8 Hz, 1H), 6.79 (d, J = 8.3 Hz, 1H), 3.49 (s, 2H); HRMS (ESI-TOF) m/z 261.0539 expected 261.0538 for $C_{12}H_{10}ClN_4O^+$ $[M+H]^+$.

S1.2. Ligand 2: 5-((5-Bromo-2-chloropyrimidin-4-yl)amino)indolin-2-one

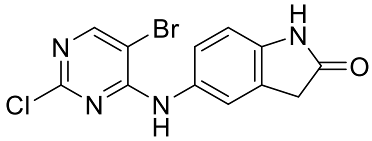

A mixture of 5-amino-2-oxindole (20 mg, 0.14 mmol), 5-Bromo-2,4-dichloropyrimidine (31 mg, 0.14 mmol), triethylamine (19 uL, 0.14 mmol) in dimethyl sulfoxide (0.5 mL) was stirred at 20 °C for 90 min. A mixture of water/ethanol (5:1 v/v; 2 mL) was added and the mixture was stirred for 3 min. The resulting beige precipitate was collected by filtration, washed with water (3 mL) and diethyl ether (3 mL) and dried under high vacuum affording the title compound (26 mg, 57%) as a beige solid. ^{1}H NMR (500 MHz, DMSO-d_6) δ 10.42 (s, 1H), 9.21 (s, 1H), 8.39 (s, 1H), 7.30 (d, J = 2.0 Hz, 1H), 7.24 (dd, J = 8.2, 2.2 Hz, 1H), 6.82 (d, J = 8.3 Hz, 1H), 3.50 (s, 2H). HRMS (ESI-TOF) m/z 338.9642 and 340.9619 expected 338.9643 and 340.9621 for $C_{12}H_9BrClN_4O$ $[M+H]^+$.

S1.3. Ligand 3: 5-((5,6-Dichloropyrimidin-4-yl)amino)indolin-2-one

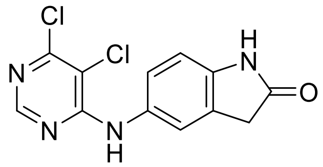

A mixture of 5-amino-2-oxindole (20 mg, 0.14 mmol), 4,5,6-trichloropyrimidine (25 mg, 0.14 mmol), triethylamine (19 uL, 0.14 mmol) in dimethyl sulfoxide (0.5 mL) was stirred at 20 °C for 90 min. A mixture of water/ethanol (5:1 v/v; 2 mL) was added and the mixture was stirred for 3 min. The resulting beige precipitate was collected by filtration, washed with water (3 mL) and diethyl ether (3 mL) and dried under high vacuum affording the title compound (25 mg, 63%) as beige solid. ^{1}H NMR (500 MHz, DMSO-d_6) δ 10.39 (s, 1H), 9.34 (s, 1H), 8.26 (s, 1H), 7.37 (d, J = 2.1 Hz, 1H), 7.26 (dd, J = 8.3, 2.1 Hz, 1H), 6.80 (d, J = 8.3 Hz, 1H), 3.50 (s, 2H). HRMS (ESI-TOF) m/z 295.0158 expected 295.0148 for $C_{12}H_9Cl_2N_4O$ $[M+H]^+$.

S1.4. Ligand 4: 5-((2,5-Dichloropyridin-4-yl)amino)indolin-2-one

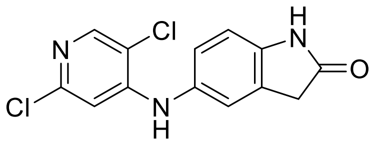

A mixture of 5-amino-2-oxindole (20 mg, 0.14 mmol), 2,4,5-trichloropyridine (25 mg, 0.14 mmol), and triethylamine (20 uL, 0.14 mmol) in dimethyl sulfoxide (0.5 mL) was stirred at rt overnight. Further 2,4,5-trichloropyridine (25 mg, 0.14 mmol) and triethylamine (20 uL, 0.14 mmol) was added, and the resulting mixture heated at 110 °C under microwave irradiation for 15 min, then to 130 °C for 1h, and then to 150 °C for 1h. The mixture was purified by reverse-phase flash chromatography (Biotage 12g SNAP Ultra C-18, 30–80% methanol in water (0.1% formic acid modifier)). Fractions containing product were combined and evaporated to give the title compound (5 mg, 13%). ^{1}H NMR (500 MHz, DMSO-d_6) δ 10.46 (s, 1H), 8.55 (s, 1H), 8.12 (s, 1H), 7.14 (d, J = 2.0 Hz, 1H), 7.09 (dd, J = 8.2, 2.2 Hz, 1H), 6.87 (d, J = 8.2 Hz, 1H), 6.48 (s, 1H), 3.52 (s, 2H). MS (ES+) m/z 294.0202 expected 294.0195 for $C_{13}H_{10}Cl_2N_3O$ $[M+H]^+$.

S1.5. Ligand 5: 5-((2-Chloro-5-fluoropyrimidin-4-yl)amino)indolin-2-one

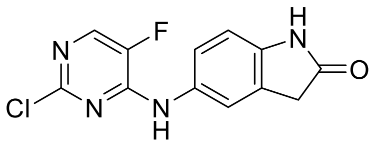

5-Amino-2-oxindole (20 mg, 0.14 mmol), 2,4-Dichloro-5-fluoropyrimidine (23 mg, 0.14 mmol), triethylamine (20 uL, 0.14 mmol) in dimethyl sulfoxide (0.5 mL) was stirred at rt

overnight, then diluted with DMSO (0.5 mL) and purified using reverse phase flash chromatography (Biotage 12g SNAP Ultra C18, 20–60% methanol in water, 0.1% formic acid modifier) to give the title compound (19 mg, 48%) as pale pink solid. ^{1}H NMR (500 MHz, DMSO-d_6) δ 10.39 (s, 1H), 9.86 (s, 1H), 8.25 (d, J = 3.5 Hz, 1H), 7.48 (d, J = 2.0 Hz, 1H), 7.40 (dd, J = 8.4, 2.3 Hz, 1H), 6.82 (d, J = 8.3 Hz, 1H), 3.51 (s, 2H). HRMS (ESI-TOF) m/z 279.0441 expected 279.0443 for $C_{12}H_9ClFN_4O$ [M+H]$^+$.

S1.6. Ligand 6: 5-((5-Chloro-2-(dimethylamino)pyrimidin-4-yl)amino)indolin-2-one

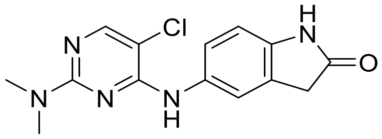

To a mixture of 5-((2,5-dichloropyrimidin-4-yl)amino)indolin-2-one (61 mg, 0.21 mmol), 3-picolylamine (0.10 mL, 1.00 mmol) and triethylamine (51 uL, 0.37 mmol) under argon was added DMF (0.6 mL) and the resulting mixture heated to 120 °C under microwave irradiation for 1 h. Two products formed – one product from addition of picolylamine into the pyrimidine, and also the product from dimethylamine (formed *in situ* through decomposition of DMF) adding into the pyrimidine. The reaction mixture was allowed to cool to rt and purified by HPLC (ACE 5 C18-PFP 250 x 21.2 mm column; 15 min gradient of 90:10 to 0:100 water:methanol (both modified with 0.1 % formic acid); flow rate 20 mLmin^{-1}; Agilent 6120 MS-Prep LC) to give the title compound as a beige solid (24 mg, 38%). ^{1}H NMR (500 MHz, DMSO-d_6) δ 10.32 (s, 1H), 8.51 (s, 1H), 7.96 (s, 1H), 7.52 (s, 1H), 7.44 (dd, J= 8.3, 1.6 Hz, 1H), 6.75 (d, J = 8.3 Hz, 1H), 3.46 (s, 2H), 3.00 (s, 6H); HRMS (ESI-TOF) m/z 304.0954 expected 304.0960 for $C_{14}H_{15}ClN_5O^+$ [M+H]$^+$.

S1.7. Ligand 7: 4-Chloro-6-((2-oxoindolin-5-yl)amino)pyrimidine-5-carbonitrile (N5065-44)

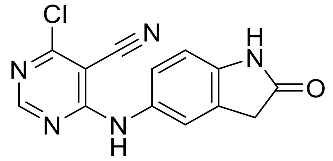

A mixture of 5-amino-2-oxindole (20 mg, 0.14 mmol, 4,6-dichloropyrimidine-5-carbonitrile (24 mg, 0.14 mmol), triethylamine (19 uL, 0.14 mmol) in dimethyl sulfoxide (0.5 mL) was stirred at 20 °C for 90 min. A mixture of water/ethanol (5:1 v/v; 2 mL) was added and the mixture was stirred for 3 min. The resulting beige precipitate was collected by filtration,

washed with water (3 mL) and diethyl ether (3 mL) and dried under high vacuum affording the title compound (28 mg, 74%) as a beige solid. ^{1}H NMR (500 MHz, DMSO-d_6) δ 10.43 (s, 1H), 10.13 (s, 1H), 8.48 (s, 1H), 7.30 (d, J = 2.0 Hz, 1H), 7.21 (dd, J = 8.2, 2.1 Hz, 1H), 6.81 (d, J = 8.2 Hz, 1H), 3.50 (s, 2H). MS (ES+) m/z 286.0506, expected 286.0496 for $C_{13}H_9ClN_5O$ $[M+H]^+$.

S1.8. Ligand 8: 5-((5-Chloro-2-(methylthio)pyrimidin-4-yl)amino)indolin-2-one

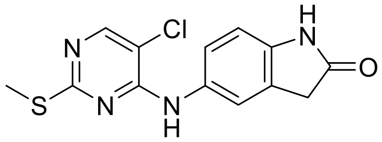

A mixture of 5-amino-2-oxindole (20 mg, 0.14 mmol, 4,5-dichloro-2-(methylsulfanyl)pyrimidine (27 mg, 0.14 mmol), triethylamine (19 uL, 0.14 mmol) in dimethyl sulfoxide (0.5 mL) was stirred at 20 °C for 90 min. A mixture of water/ethanol (5:1 v/v; 2 mL) was added and the mixture was stirred for 3 min. The resulting beige precipitate was collected by filtration, washed with water (3 mL) and diethyl ether (3 mL) and dried under high vacuum affording the title compound (12 mg, 29%) as beige solid. ^{1}H NMR (500 MHz, DMSO-d_6) δ 10.37 (s, 1H), 9.07 (s, 1H), 8.22 (s, 1H), 7.42 (d, J = 2.0 Hz, 1H), 7.34 (dd, J = 8.4, 2.2 Hz, 1H), 6.79 (d, J = 8.3 Hz, 1H), 3.48 (s, 2H), 2.36 (s, 3H). MS (ES+) m/z 307.0429, expected 307.0420 for $C_{13}H_{12}ClN_4OS$ $[M+H]^+$.

S1.9. Ligand 9: 5-Chloro-7-((2-oxoindolin-5-yl)amino)pyrazolo[1,5-*a*]pyrimidine-3-carbonitrile

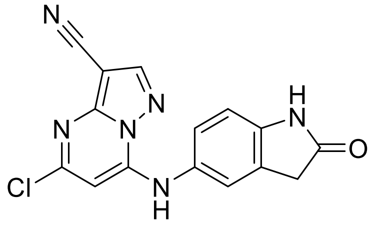

A mixture of 5-amino-2-oxindole (20 mg, 0.14 mmol), 5,7-dichloropyrazolo[1,5-*a*]pyrimidine-3-carbonitrile (29 mg, 0.14 mmol), triethylamine (19 uL, 0.14 mmol) in dimethyl sulfoxide (0.5 mL) was stirred at 20 °C for 90 min. A mixture of water/ethanol (5:1 v/v; 2 mL) was added and the mixture was stirred for 3 min. The resulting brown precipitate was collected by filtration, washed with water (3 mL) and diethyl ether (3 mL) and dried under high vacuum affording the title compound (30 mg, 68%) as brown solid. ^{1}H NMR (500 MHz, DMSO-d_6) δ 10.73 (s, 1H), 10.54 (s, 1H), 8.78 (s, 1H), 7.27 (d, J = 2.0 Hz, 1H), 7.23 (dd, J = 8.2, 2.2 Hz, 1H), 6.92 (d, J = 8.2 Hz, 1H), 6.15 (s, 1H), 3.56 (s, 2H). HRMS (ESI-TOF) m/z 325.0600 expected 325.0599 for $C_{15}H_{10}ClN_6O$ $[M+H]^+$.

S2. MSOX crystallography statistics tables

S2.1. BCL6 ligand 1

Sweep	Sweep 1	Sweep 2	Sweep 3	Sweep 4	Sweep 5
PDB Code	7GUD	7GUE	7GUF	7GUG	7GUH
Dose (MGy)[a]	1.51	3.02	4.53	6.04	7.54
Space group	$P6_122$	$P6_122$	$P6_122$	$P6_122$	$P6_122$
Unit Cell (a=b,c) (Å)	67.51, 165.98	67.55, 166.08	67.54, 166.12	67.55, 166.15	67.55, 166.22
Unit Cell (α=β,γ) (°)	90.00, 120.00	90.00, 120.00	90.00, 120.00	90.00, 120.00	90.00, 120.00
Beamline	DLS I24	DLS I24	DLS I24	DLS I24	DLS I24
Wavelength (Å)	0.9686	0.9686	0.9686	0.9686	0.9686
Resolution (Å) [b]	33.84-1.80 (1.84-1.80)	33.86-1.80 (1.84-1.80)	33.86-1.80 (1.84-1.80)	33.87-1.80 (1.84-1.80)	33.88-1.80 (1.84-1.80)
Unique Reflections [b]	21648 (1247)	21696 (1245)	21679 (1244)	21698 (1246)	21692 (1241)
Multiplicity [b]	9.1 (9.3)	9.1 (9.3)	9.1 (9.3)	9.1 (9.3)	9.0 (9.3)
$R_{p.i.m}$ (%) [b]	2.7 (16.7)	4.5 (20.5)	3.0 (20.3)	3.1 (27.8)	2.6 (29.5)
R_{meas} (%) [b]	8.1 (51.7)	13.7 (63.1)	9.1 (62.9)	9.5 (85.5)	7.9 (91.4)
CC1/2 [b,c]	0.998 (0.946)	0.990 (0.934)	0.996 (0.943)	0.997 (0.881)	0.998 (0.874)
$I / \sigma(I)$ [b]	14.5 (4.2)	10.4 (3.6)	13.2 (3.4)	11.9 (2.8)	14.1 (2.6)
Completeness (%) [b]	100.0 (100.0)	100.0 (100.0)	100.0 (100.0)	100.0 (100.0)	100.0 (100.0)
Wilson B (Å^2)	18.9	19.7	20.5	20.9	21.2
**Refinement**					
R_{work} / R_{free}	18.03 / 19.85	18.32 / 20.62	17.95 / 20.24	17.95 / 20.28	17.78 / 20.13
No. Atoms					
Protein	1176	1176	1176	1176	1176
Ligand/ion	26	26	26	26	26
Water	181	181	181	181	181
Ramachandran (#,%)					
Allowed	121 (97.58%)	121 (97.58%)	121 (97.58%)	121 (97.58%)	121 (97.58%)
Generally allowed	3 (2.42%)	3 (2.42%)	3 (2.42%)	3 (2.42%)	3 (2.42%)
Disallowed	0 (0.00%)	0 (0.00%)	0 (0.00%)	0 (0.00%)	0 (0.00%)
B-factors					
Protein	27.52	28.54	29.32	30.60	30.79
Ligand/ion	25.39	26.24	27.27	28.88	29.48
Water	45.12	46.69	48.18	50.08	50.84
R.M.S. deviations					
Bond lengths (Å)	0.01	0.01	0.01	0.01	0.01
Bond angles (°)	0.90	0.91	0.92	0.92	0.91

[a] Diffraction Weighted Dose as described by Zeldin *et al.*

[b] Values in parentheses are for the highest resolution shell.

[c] Half-dataset correlation coefficient, see: Karplus, P. A.; Diederichs, K. Linking crystallographic model and data quality. *Science* **2012**, *336*, 1030–1033.

Sweep	Sweep 6	Sweep 7	Sweep 8	Sweep 9	Sweep 10
**PDB Code**	**7GUI**	**7GUJ**	**7GUK**	**7GUL**	**7GUM**
Dose (MGy)[a]	9.05	10.56	12.07	13.58	15.08
Space group	$P6_122$	$P6_122$	$P6_122$	$P6_122$	$P6_122$
Unit Cell (a=b,c) (Å)	67.58, 166.21	67.59, 166.26	67.55, 166.24	67.58, 166.33	67.58, 166.15
Unit Cell (α=β,γ) (°)	90.00, 120.00	90.00, 120.00	90.00, 120.00	90.00, 120.00	90.00, 120.00
Beamline	DLS I24	DLS I24	DLS I24	DLS I24	DLS I24
Wavelength (Å)	0.9686	0.9686	0.9686	0.9686	0.9686
Resolution (Å)[b]	33.88-1.80 (1.84-1.80)	33.89-1.80 (1.84-1.80)	33.88-1.80 (1.84-1.80)	33.90-1.80 (1.84-1.80)	33.87-1.80 (1.84-1.80)
Unique Reflections[b]	21726 (1245)	21723 (1243)	21705 (1240)	21722 (1246)	21719 (1245)
Multiplicity[b]	9.1 (9.3)	9.1 (9.3)	9.0 (9.3)	9.0 (9.3)	9.1 (9.3)
$R_{p.i.m}$ (%)[b]	4.1 (35.7)	3.1 (40.9)	3.3 (50.7)	2.9 (50.7)	4.5 (53.6)
R_{meas} (%)[b]	12.3 (110.4)	9.4 (126.9)	10.0 (155.9)	8.7 (157.3)	13.8 (165.5)
CC1/2[b,c]	0.992 (0.821)	0.998 (0.833)	0.998 (0.685)	0.999 (0.697)	0.996 (0.704)
$I / \sigma(I)$[b]	9.6 (2.1)	11.8 (1.9)	10.6 (1.6)	12.3 (1.5)	8.0 (1.4)
Completeness (%)[b]	100.0 (100.0)	100.0 (100.0)	100.0 (100.0)	100.0 (100.0)	100.0 (100.0)
Wilson B ($Å^2$)	22.2	23.0	24.1	24.2	24.6
**Refinement**					
R_{work} / R_{free}	18.24 / 20.87	18.07 / 20.60	18.19 / 20.95	17.86 / 20.42	18.15 / 21.22
No. Atoms					
Protein	1176	1176	1176	1176	1176
Ligand/ion	26	26	26	26	26
Water	181	181	181	181	181
Ramachandran (#,%)					
Allowed	121 (97.58%)	121 (97.58%)	121 (97.58%)	121 (97.58%)	121 (97.58%)
Generally allowed	3 (2.42%)	3 (2.42%)	3 (2.42%)	3 (2.42%)	3 (2.42%)
Disallowed	0 (0.00%)	0 (0.00%)	0 (0.00%)	0 (0.00%)	0 (0.00%)
B-factors					
Protein	32.17	32.77	33.87	33.55	33.94
Ligand/ion	30.25	30.93	32.63	32.75	32.01
Water	52.28	53.16	55.07	55.05	55.02
R.M.S. deviations					
Bond lengths (Å)	0.01	0.01	0.01	0.01	0.01
Bond angles (°)	0.92	0.92	0.91	0.90	0.90

[a] Diffraction Weighted Dose as described by Zeldin *et al.*

[b] Values in parentheses are for the highest resolution shell.

[c] Half-dataset correlation coefficient, see: Karplus, P. A.; Diederichs, K. Linking crystallographic model and data quality. *Science* **2012**, *336*, 1030–1033.

Sweep	Sweep 11	Sweep 12	Sweep 13	Sweep 14	Sweep 15
**PDB Code**	**7GUN**	**7GUO**	**7GUP**	**7GUQ**	**7GUR**
Dose (MGy)[a]	16.60	18.11	19.62	21.12	22.63
Space group	$P6_122$	$P6_122$	$P6_122$	$P6_122$	$P6_122$
Unit Cell (a=b,c) (Å)	67.61, 166.28	67.54, 166.21	67.60, 166.36	67.57, 166.07	67.63, 166.27
Unit Cell (α=β,γ) (°)	90.00, 120.00	90.00, 120.00	90.00, 120.00	90.00, 120.00	90.00, 120.00
Beamline	DLS I24	DLS I24	DLS I24	DLS I24	DLS I24
Wavelength (Å)	0.9686	0.9686	0.9686	0.9686	0.9686
Resolution (Å) [b]	33.90-1.80 (1.84-1.80)	33.87-1.80 (1.84-1.80)	33.91-1.80 (1.84-1.80)	33.86-1.80 (1.84-1.80)	33.90-1.80 (1.84-1.80)
Unique Reflections [b]	21743 (1252)	21699 (1243)	21741 (1253)	21706 (1249)	21752 (1251)
Multiplicity [b]	9.0 (9.3)	9.0 (9.3)	9.0 (9.3)	9.1 (9.3)	9.0 (9.3)
$R_{p.i.m}$ (%) [b]	3.5 (68.8)	3.9 (79.0)	3.4 (81.0)	5.1 (72.7)	4.1 (104.6)
R_{meas} (%) [b]	10.6 (213.1)	11.8 (242.9)	10.4 (251.1)	15.6 (224.0)	12.5 (323.6)
CC1/2 [b,c]	0.998 (0.659)	0.998 (0.536)	0.999 (0.507)	0.993 (0.602)	0.997 (0.458)
$I / \sigma(I)$ [b]	10.1 (1.1)	8.6 (1.0)	10.0 (0.9)	6.8 (1.0)	8.2 (0.6)
Completeness (%) [b]	100.0 (100.0)	100.0 (100.0)	100.0 (100.0)	100.0 (100.0)	100.0 (100.0)
Wilson B ($Å^2$)	26.3	27.2	27.4	27.7	29.7
**Refinement**					
R_{work} / R_{free}	18.15 / 20.79	18.12 / 21.27	18.02 / 20.62	17.92 / 20.74	18.07 / 21.07
No. Atoms					
Protein	1176	1176	1176	1176	1176
Ligand/ion	26	26	26	26	26
Water	181	181	181	181	181
Ramachandran (#,%)					
Allowed	121 (97.58%)	121 (97.58%)	121 (97.58%)	121 (97.58%)	121 (97.58%)
Generally allowed	3 (2.42%)	3 (2.42%)	3 (2.42%)	3 (2.42%)	3 (2.42%)
Disallowed	0 (0.00%)	0 (0.00%)	0 (0.00%)	0 (0.00%)	0 (0.00%)
B-factors					
Protein	35.41	36.05	36.27	35.69	37.75
Ligand/ion	33.41	34.57	35.07	33.95	36.58
Water	56.69	57.88	58.54	57.24	59.18
R.M.S. deviations					
Bond lengths (Å)	0.01	0.01	0.01	0.01	0.01
Bond angles (°)	0.92	0.91	0.91	0.90	0.91

[a] Diffraction Weighted Dose as described by Zeldin *et al.*

[b] Values in parentheses are for the highest resolution shell.

[c] Half-dataset correlation coefficient, see: Karplus, P. A.; Diederichs, K. Linking crystallographic model and data quality. *Science* **2012**, *336*, 1030–1033.

S2.2. BCL6 ligand 2

Sweep	Sweep 1	Sweep 2	Sweep 3	Sweep 4	Sweep 5
**PDB Code**	**7GUS**	**7GUT**	**7GUU**	**7GUV**	**7GUW**
Dose (MGy)[a]	2.27	4.55	6.82	9.10	11.37
Space group	$P6_122$	$P6_122$	$P6_122$	$P6_122$	$P6_122$
Unit Cell (a=b,c) (Å)	67.74, 166.68	67.57, 166.46	67.75, 166.78	67.60, 166.53	67.77, 166.81
Unit Cell (α=β,γ) (°)	90.00, 120.00	90.00, 120.00	90.00, 120.00	90.00, 120.00	90.00, 120.00
Beamline	DLS I24	DLS I24	DLS I24	DLS I24	DLS I24
Wavelength (Å)	0.9686	0.9686	0.9686	0.9686	0.9686
Resolution (Å) [b]	33.97-1.75 (1.78-1.75)	33.91-1.75 (1.78-1.75)	33.99-1.75 (1.78-1.75)	33.93-1.75 (1.78-1.75)	33.99-1.75 (1.78-1.75)
Unique Reflections [b]	23750 (1284)	23604 (1276)	23773 (1295)	23647 (1292)	23790 (1285)
Multiplicity [b]	9.1 (9.3)	9.1 (9.3)	9.1 (9.3)	9.1 (9.4)	9.1 (9.3)
$R_{p.i.m}$ (%) [b]	2.6 (20.0)	2.2 (35.7)	2.7 (31.9)	2.3 (35.8)	2.6 (31.1)
R_{meas} (%) [b]	7.8 (61.9)	6.8 (110.8)	8.0 (99.0)	6.9 (111.6)	7.8 (96.5)
CC1/2 [b,c]	0.998 (0.943)	0.999 (0.827)	0.998 (0.843)	0.998 (0.856)	0.998 (0.878)
$I / \sigma(I)$ [b]	14.4 (3.4)	16.1 (2.4)	13.5 (2.4)	15.7 (2.2)	13.6 (2.3)
Completeness (%) [b]	100.0 (100.0)	100.0 (100.0)	100.0 (100.0)	100.0 (100.0)	100.0 (100.0)
Wilson B (Å^2)	20.2	19.6	20.2	21.2	21.7
**Refinement**					
R_{work} / R_{free}	18.46 / 21.54	18.38 / 21.18	18.02 / 20.32	18.15 / 20.88	18.08 / 20.77
No. Atoms					
Protein	1173	1173	1173	1173	1173
Ligand/ion	27	27	27	27	27
Water	185	185	185	185	185
Ramachandran (#,%)					
Allowed	119 (95.97%)	120 (96.77%)	119 (95.97%)	120 (96.77%)	120 (96.77%)
Generally allowed	5 (4.03%)	4 (3.23%)	5 (4.03%)	4 (3.23%)	4 (3.23%)
Disallowed	0 (0.00%)	0 (0.00%)	0 (0.00%)	0 (0.00%)	0 (0.00%)
B-factors					
Protein	28.80	29.50	30.13	30.84	31.77
Ligand/ion	26.30	27.38	28.35	29.04	30.25
Water	45.69	46.98	48.43	49.42	50.89
R.M.S. deviations					
Bond lengths (Å)	0.01	0.01	0.01	0.01	0.01
Bond angles (°)	0.87	0.88	0.88	0.88	0.88

[a] Diffraction Weighted Dose as described by Zeldin *et al.*

[b] Values in parentheses are for the highest resolution shell.

[c] Half-dataset correlation coefficient, see: Karplus, P. A.; Diederichs, K. Linking crystallographic model and data quality. *Science* **2012**, *336*, 1030–1033.

Sweep	Sweep 6	Sweep 7	Sweep 8	Sweep 9	Sweep 10
**PDB Code**	**7GUX**	**7GUY**	**7GUZ**	**7GVO**	**7GV1**
Dose (MGy)[a]	13.65	15.92	18.20	20.47	22.75
Space group	$P6_122$	$P6_122$	$P6_122$	$P6_122$	$P6_122$
Unit Cell (a=b,c) (Å)	67.61, 166.57	67.78, 166.86	67.64, 166.59	67.77, 166.81	67.64, 166.63
Unit Cell (α=β,γ) (°)	90.00, 120.00	90.00, 120.00	90.00, 120.00	90.00, 120.00	90.00, 120.00
Beamline	DLS I24	DLS I24	DLS I24	DLS I24	DLS I24
Wavelength (Å)	0.9686	0.9686	0.9686	0.9686	0.9686
Resolution (Å) [b]	33.94-1.75 (1.78-1.75)	34.00-1.75 (1.78-1.75)	33.94-1.75 (1.78-1.75)	34.00-1.75 (1.78-1.75)	33.95-1.75 (1.78-1.75)
Unique Reflections [b]	23650 (1290)	23796 (1282)	23674 (1280)	23791 (1285)	23675 (1279)
Multiplicity [b]	9.1 (9.3)	9.1 (9.3)	9.1 (9.4)	9.0 (9.3)	9.1 (9.3)
$R_{p.i.m}$ (%) [b]	2.3 (52.1)	2.7 (43.8)	2.5 (56.5)	2.8 (48.6)	2.6 (75.3)
R_{meas} (%) [b]	7.1 (162.3)	8.3 (135.9)	7.5 (175.9)	8.6 (150.4)	7.8 (234.1)
CC1/2 [b,c]	0.999 (0.684)	0.998 (0.719)	0.998 (0.706)	0.998 (0.743)	0.999 (0.571)
$I / \sigma(I)$ [b]	15.1 (1.8)	12.5 (1.8)	14.2 (1.4)	12.1 (1.5)	13.5 (1.2)
Completeness (%) [b]	100.0 (100.0)	100.0 (100.0)	100.0 (100.0)	100.0 (100.0)	100.0 (100.0)
Wilson B (Å^2)	21.7	22.7	23.5	24.2	24.2
**Refinement**					
R_{work} / R_{free}	18.02 / 20.41	17.83 / 20.36	17.99 / 20.50	18.07 / 20.92	17.82 / 20.34
No. Atoms					
Protein	1173	1173	1173	1173	1173
Ligand/ion	27	27	27	27	27
Water	185	185	185	185	185.
Ramachandran (#,%)					
Allowed	120 (96.77%)	119 (95.97%)	120 (96.77%)	120 (96.77%)	120 (96.77%)
Generally allowed	4 (3.23%)	5 (4.03%)	4 (3.23%)	4 (3.23%)	4 (3.23%)
Disallowed	0 (0.00%)	0 (0.00%)	0 (0.00%)	0 (0.00%)	0 (0.00%)
B-factors					
Protein	32.20	32.97	33.84	34.41	34.72
Ligand/ion	30.56	31.77	32.64	33.19	33.48
Water	51.86	52.61	53.68	54.55	54.84
R.M.S. deviations					
Bond lengths (Å)	0.01	0.01	0.01	0.01	0.01
Bond angles (°)	0.88	0.88	0.89	0.88	0.88

[a] Diffraction Weighted Dose as described by Zeldin *et al.*

[b] Values in parentheses are for the highest resolution shell.

[c] Half-dataset correlation coefficient, see: Karplus, P. A.; Diederichs, K. Linking crystallographic model and data quality. *Science* **2012**, *336*, 1030–1033.

Sweep	Sweep 11	Sweep 12	Sweep 13	Sweep 14	Sweep 15
**PDB Code**	**7GV2**	**7GV3**	**7GV4**	**7GV5**	**7GV6**
Dose (MGy)[a]	25.02	27.30	29.57	31.85	34.12
Space group	$P6_122$	$P6_122$	$P6_122$	$P6_122$	$P6_122$
Unit Cell (a=b,c) (Å)	67.79, 166.89	67.67, 166.61	67.76, 166.73	67.66, 166.65	67.79, 166.86
Unit Cell (α=β,γ) (°)	90.00, 120.00	90.00, 120.00	90.00, 120.00	90.00, 120.00	90.00, 120.00
Beamline	DLS I24	DLS I24	DLS I24	DLS I24	DLS I24
Wavelength (Å)	0.9686	0.9686	0.9686	0.9686	0.9686
Resolution (Å) [b]	34.01-1.75 (1.78-1.75)	33.95-1.75 (1.78-1.75)	33.98-1.75 (1.78-1.75)	33.95-1.75 (1.78-1.75)	34.00-1.75 (1.78-1.75)
Unique Reflections [b]	23807 (1282)	23701 (1289)	23773 (1287)	23668 (1283)	23801 (1281)
Multiplicity [b]	9.0 (9.3)	9.1 (9.3)	9.0 (9.3)	9.1 (9.3)	9.0 (9.3)
$R_{p.i.m}$ (%) [b]	2.9 (65.0)	2.8 (84.8)	3.0 (64.3)	2.8 (109.0)	3.1 (76.4)
R_{meas} (%) [b]	8.7 (201.2)	8.6 (263.5)	9.2 (198.3)	8.6 (339.5)	9.4 (236.4)
CC1/2 [b,c]	0.999 (0.597)	0.998 (0.532)	0.999 (0.628)	0.999 (0.355)	0.999 (0.411)
$I / \sigma(I)$ [b]	11.7 (1.2)	12.1 (0.9)	11.1 (1.1)	12.3 (0.8)	10.5 (1.0)
Completeness (%) [b]	100.0 (100.0)	100.0 (100.0)	100.0 (100.0)	100.0 (100.0)	100.0 (100.0)
Wilson B (Å^2)	24.9	26.6	26.8	27.0	27.3
**Refinement**					
R_{work} / R_{free}	17.91 / 20.33	17.88 / 20.60	17.72 / 20.45	17.65 / 20.13	17.75 / 20.22
No. Atoms					
Protein	1173	1173	1173	1173	1173
Ligand/ion	27	27	27	27	27
Water	185	185	185	185	185
Ramachandran (#,%)					
Allowed	120 (96.77%)	120 (96.77%)	121 (97.58%)	119 (95.97%)	121 (97.58%)
Generally allowed	4 (3.23%)	4 (3.23%)	3 (2.42%)	5 (4.03%)	3 (2.42%)
Disallowed	0 (0.00%)	0 (0.00%)	0 (0.00%)	0 (0.00%)	0 (0.00%)
B-factors					
Protein	35.24	36.23	35.91	36.70	36.60
Ligand/ion	34.18	35.23	34.44	35.46	35.43
Water	55.66	57.12	56.33	56.80	57.62
R.M.S. deviations					
Bond lengths (Å)	0.01	0.01	0.01	0.01	0.01
Bond angles (°)	0.88	0.88	0.88	0.88	0.88

[a] Diffraction Weighted Dose as described by Zeldin *et al.*
[b] Values in parentheses are for the highest resolution shell.
[c] Half-dataset correlation coefficient, see: Karplus, P. A.; Diederichs, K. Linking crystallographic model and data quality. *Science* **2012**, *336*, 1030–1033.

S2.3. BCL6 ligand 3

Sweep	Sweep 1	Sweep 2	Sweep 3	Sweep 4	Sweep 5
PDB Code	7GV7	7GV8	7GV9	7GVA	7GVB
Dose (MGy)[a]	1.45	2.90	4.35	5.80	7.25
Space group	$P6_122$	$P6_122$	$P6_122$	$P6_122$	$P6_122$
Unit Cell (a=b,c) (Å)	67.58, 166.30	67.55, 166.26	67.57, 166.33	67.57, 166.31	67.62, 166.50
Unit Cell (α=β,γ) (°)	90.00, 120.00	90.00, 120.00	90.00, 120.00	90.00, 120.00	90.00, 120.00
Beamline	DLS I24	DLS I24	DLS I24	DLS I24	DLS I24
Wavelength (Å)	0.9686	0.9686	0.9686	0.9686	0.9686
Resolution (Å)[b]	33.89-1.85 (1.89-1.85)	33.88-1.85 (1.89-1.85)	33.90-1.85 (1.89-1.85)	33.89-1.85 (1.89-1.85)	33.93-1.85 (1.89-1.85)
Unique Reflections[b]	19809 (1219)	20061 (1213)	19801 (1220)	20081 (1219)	19856 (1218)
Multiplicity[b]	9.2 (9.3)	9.0 (9.2)	9.1 (9.2)	9.0 (9.2)	9.2 (9.3)
$R_{p.i.m}$ (%)[b]	4.5 (14.4)	3.4 (15.3)	2.8 (15.8)	3.7 (20.9)	4.1 (24.2)
R_{meas} (%)[b]	13.8 (45.0)	10.1 (46.9)	8.4 (49.4)	11.2 (63.9)	12.6 (75.9)
CC1/2[b,c]	0.990 (0.944)	0.995 (0.926)	0.998 (0.932)	0.995 (0.904)	0.993 (0.878)
$I / \sigma(I)$[b]	10.4 (4.7)	12.2 (4.6)	14.3 (4.5)	10.8 (3.4)	10.0 (3.1)
Completeness (%)[b]	99.5 (100.0)	100.0 (100.0)	99.4 (100.0)	100.0 (100.0)	99.5 (100.0)
Wilson B ($Å^2$)	20.2	20.9	21.1	21.1	22.4
**Refinement**					
R_{work} / R_{free}	17.80 / 20.04	18.02 / 19.89	17.60 / 19.19	18.12 / 20.04	17.68 / 19.70
No. Atoms					
Protein	1139	1139	1139	1139	1139
Ligand/ion	27	27	27	27	27
Water	161	161	161	161	161
Ramachandran (#,%)					
Allowed	120 (96.77%)	120 (96.77%)	119 (95.97%)	119 (95.97%)	120 (96.77%)
Generally allowed	4 (3.23%)	4 (3.23%)	5 (4.03%)	5 (4.03%)	4 (3.23%)
Disallowed	0 (0.00%)	0 (0.00%)	0 (0.00%)	0 (0.00%)	0 (0.00%)
B-factors					
Protein	27.01	27.51	28.08	29.44	31.27
Ligand/ion	27.72	28.79	29.24	30.64	32.57
Water	43.40	44.57	45.67	47.65	49.99
R.M.S. deviations					
Bond lengths (Å)	0.01	0.01	0.01	0.01	0.01
Bond angles (°)	0.88	0.89	0.89	0.89	0.89

[a] Diffraction Weighted Dose as described by Zeldin *et al.*

[b] Values in parentheses are for the highest resolution shell.

[c] Half-dataset correlation coefficient, see: Karplus, P. A.; Diederichs, K. Linking crystallographic model and data quality. *Science* **2012**, *336*, 1030–1033.

Sweep	Sweep 6	Sweep 7	Sweep 8	Sweep 9	Sweep 10
**PDB Code**	**7GVC**	**7GVD**	**7GVE**	**7GVF**	**7GVG**
Dose (MGy)[a]	8.69	10.14	11.59	13.04	14.49
Space group	$P6_122$	$P6_122$	$P6_122$	$P6_122$	$P6_122$
Unit Cell (a=b,c) (Å)	67.57, 166.37	67.61, 166.49	67.59, 166.33	67.66, 166.60	67.56, 166.37
Unit Cell (α=β,γ) (°)	90.00, 120.00	90.00, 120.00	90.00, 120.00	90.00, 120.00	90.00, 120.00
Beamline	DLS I24	DLS I24	DLS I24	DLS I24	DLS I24
Wavelength (Å)	0.9686	0.9686	0.9686	0.9686	0.9686
Resolution (Å) [b]	33.90-1.85 (1.89-1.85)	33.92-1.85 (1.89-1.85)	33.90-1.85 (1.89-1.85)	33.95-1.85 (1.89-1.85)	33.90-1.85 (1.89-1.85)
Unique Reflections [b]	20087 (1219)	19840 (1220)	20089 (1214)	19896 (1216)	20081 (1216)
Multiplicity [b]	9.0 (9.2)	9.1 (9.2)	9.0 (9.2)	9.2 (9.3)	9.0 (9.2)
$R_{p.i.m}$ (%) [b]	3.6 (27.2)	3.0 (28.8)	3.8 (38.6)	4.5 (43.7)	4.1 (45.8)
R_{meas} (%) [b]	10.9 (83.1)	9.1 (90.1)	11.3 (118.1)	13.8 (137.7)	12.4 (139.9)
CC1/2 [b,c]	0.995 (0.853)	0.998 (0.832)	0.997 (0.75)	0.993 (0.706)	0.997 (0.673)
$I / \sigma(I)$ [b]	10.6 (2.8)	12.7 (2.5)	9.8 (1.9)	8.4 (1.7)	8.9 (1.7)
Completeness (%) [b]	100.0 (100.0)	99.4 (100.0)	100.0 (100.0)	99.5 (100.0)	100.0 (100.0)
Wilson B ($Å^2$)	22.6	23.4	25.5	26.3	27.0
**Refinement**					
R_{work} / R_{free}	17.88 / 19.79	17.66 / 19.64	18.21 / 20.64	17.73 / 19.85	17.92 / 20.21
No. Atoms					
Protein	1139	1139	1139	1139	1139
Ligand/ion	27	27	27	27	27
Water	161	161	161	161	161
Ramachandran (#,%)					
Allowed	120 (96.77%)	119 (95.97%)	119 (95.97%)	120 (96.77%)	120 (96.77%)
Generally allowed	4 (3.23%)	5 (4.03%)	5 (4.03%)	4 (3.23%)	4 (3.23%)
Disallowed	0 (0.00%)	0 (0.00%)	0 (0.00%)	0 (0.00%)	0 (0.00%)
B-factors					
Protein	31.14	32.16	33.66	35.12	34.09
Ligand/ion	32.43	33.69	35.02	36.77	35.09
Water	50.36	51.61	53.30	54.99	54.02
R.M.S. deviations					
Bond lengths (Å)	0.01	0.01	0.01	0.01	0.01
Bond angles (°)	0.88	0.89	0.89	0.90	0.88

[a] Diffraction Weighted Dose as described by Zeldin *et al.*

[b] Values in parentheses are for the highest resolution shell.

[c] Half-dataset correlation coefficient, see: Karplus, P. A.; Diederichs, K. Linking crystallographic model and data quality. *Science* **2012**, *336*, 1030–1033.

Sweep	Sweep 11	Sweep 12	Sweep 13	Sweep 14	Sweep 15
**PDB Code**	**7GVH**	**7GVI**	**7GVJ**	**7GVK**	**7GVL**
Dose (MGy)[a]	15.94	17.39	18.84	20.29	21.74
Space group	$P6_122$	$P6_122$	$P6_122$	$P6_122$	$P6_122$
Unit Cell (a=b,c) (Å)	67.64, 166.57	67.59, 166.28	67.68, 166.65	67.55, 166.33	67.65, 166.58
Unit Cell (α=β,γ) (°)	90.00, 120.00	90.00, 120.00	90.00, 120.00	90.00, 120.00	90.00, 120.00
Beamline	DLS I24	DLS I24	DLS I24	DLS I24	DLS I24
Wavelength (Å)	0.9686	0.9686	0.9686	0.9686	0.9686
Resolution (Å) [b]	33.94-1.85 (1.89-1.85)	33.89-1.85 (1.89-1.85)	33.96-1.85 (1.89-1.85)	33.89-1.85 (1.89-1.85)	33.94-1.85 (1.89-1.85)
Unique Reflections [b]	19870 (1221)	20089 (1219)	19916 (1218)	20072 (1216)	19883 (1218)
Multiplicity [b]	9.1 (9.2)	9.0 (9.2)	9.1 (9.2)	9.0 (9.2)	9.1 (9.2)
$R_{p.i.m}$ (%) [b]	3.4 (52.1)	4.3 (60.1)	4.4 (77.4)	4.7 (65.4)	3.8 (83.1)
R_{meas} (%) [b]	10.3 (163.1)	13.0 (183.4)	13.4 (243.0)	14.0 (199.5)	11.8 (259.4)
CC1/2 [b,c]	0.998 (0.650)	0.996 (0.568)	0.996 (0.459)	0.996 (0.534)	0.999 (0.420)
$I / \sigma(I)$ [b]	10.9 (1.4)	8.1 (1.2)	8.0 (1.0)	7.7 (1.2)	9.6 (0.9)
Completeness (%) [b]	99.4 (100.0)	100.0 (100.0)	99.5 (100.0)	100.0 (100.0)	99.4 (99.9)
Wilson B (Å^2)	27.1	30.5	30.1	30.7	30.2
**Refinement**					
R_{work} / R_{free}	18.00 / 20.16	18.32 / 20.75	18.13 / 20.35	18.19 / 20.40	18.54 / 20.48
No. Atoms					
Protein	1139	1139	1139	1139	1139
Ligand/ion	27	27	27	27	27
Water	161	161	161	161	161
Ramachandran (#,%)					
Allowed	119 (95.97%)	121 (97.58%)	120 (96.77%)	120 (96.77%)	120 (96.77%)
Generally allowed	5 (4.03%)	3 (2.42%)	4 (3.23%)	4 (3.23%)	4 (3.23%)
Disallowed	0 (0.00%)	0 (0.00%)	0 (0.00%)	0 (0.00%)	0 (0.00%)
B-factors					
Protein	35.21	36.51	37.72	35.73	36.55
Ligand/ion	36.67	37.32	39.49	36.66	37.95
Water	55.20	56.56	58.23	55.98	56.86
R.M.S. deviations					
Bond lengths (Å)	0.01	0.01	0.01	0.01	0.01
Bond angles (°)	0.89	0.89	0.90	0.88	0.89

[a] Diffraction Weighted Dose as described by Zeldin *et al.*

[b] Values in parentheses are for the highest resolution shell.

[c] Half-dataset correlation coefficient, see: Karplus, P. A.; Diederichs, K. Linking crystallographic model and data quality. *Science* **2012**, *336*, 1030–1033.

S2.4. BCL6 ligand 4

**Sweep**	**Sweep 1**	**Sweep 2**	**Sweep 3**	**Sweep 4**	**Sweep 5**
**PDB Code**	**7GVM**	**7GVN**	**7GVO**	**7GVP**	**7GVQ**
Dose (MGy)[a]	1.40	2.81	4.21	5.62	7.02
Space group	$P6_122$	$P6_122$	$P6_122$	$P6_122$	$P6_122$
Unit Cell (a=b,c) (Å)	67.74, 166.87	67.60, 166.82	67.77, 167.02	67.63, 166.89	67.79, 167.09
Unit Cell (α=β,γ) (°)	90.00, 120.00	90.00, 120.00	90.00, 120.00	90.00, 120.00	90.00, 120.00
Beamline	DLS I24	DLS I24	DLS I24	DLS I24	DLS I24
Wavelength (Å)	0.9686	0.9686	0.9686	0.9686	0.9686
Resolution (Å) [b]	34.00-1.90 (1.94-1.90)	33.97-1.90 (1.94-1.90)	34.02-1.90 (1.94-1.90)	33.98-1.90 (1.94-1.90)	34.04-1.90 (1.94-1.90)
Unique Reflections [b]	18736 (1180)	18655 (1175)	18760 (1177)	18670 (1173)	18782 (1171)
Multiplicity [b]	9.0 (9.4)	9.1 (9.4)	9.0 (9.4)	9.1 (9.4)	9.0 (9.4)
$R_{p.i.m}$ (%) [b]	2.0 (14.8)	2.1 (16.1)	1.9 (16.9)	2.4 (20.5)	2.1 (22.6)
R_{meas} (%) [b]	6.0 (45.9)	6.5 (49.7)	5.9 (52.2)	7.1 (63.3)	6.4 (70.1)
CC1/2 [b,c]	1.000 (0.941)	0.999 (0.936)	1.000 (0.933)	0.999 (0.917)	1.000 (0.889)
$I / \sigma(I)$ [b]	18.5 (4.4)	16.9 (4.0)	19.1 (4.1)	15.7 (3.0)	17.1 (3.0)
Completeness (%) [b]	100.0 (100.0)	100.0 (100.0)	100.0 (100.0)	100.0 (100.0)	100.0 (100.0)
Wilson B ($Å^2$)	26.2	26.1	26.7	26.4	28.0
**Refinement**					
R_{work} / R_{free}	19.62 / 22.33	19.27 / 21.74	19.18 / 20.87	19.22 / 21.25	19.24 / 21.15
No. Atoms					
Protein	1078	1078	1078	1078	1078
Ligand/ion	27	27	27	27	27
Water	138	138	138	138	138
Ramachandran (#,%)					
Allowed	120 (98.36%)	119 (97.54%)	119 (97.54%)	120 (98.36%)	120 (98.36%)
Generally allowed	2 (1.64%)	3 (2.46%)	3 (2.46%)	2 (1.64%)	2 (1.64%)
Disallowed	0 (0.00%)	0 (0.00%)	0 (0.00%)	0 (0.00%)	0 (0.00%)
B-factors					
Protein	36.12	36.01	37.05	37.16	39.07
Ligand/ion	32.80	32.54	33.87	33.85	35.99
Water	49.89	50.66	51.95	52.51	54.74
R.M.S. deviations					
Bond lengths (Å)	0.01	0.01	0.01	0.01	0.01
Bond angles (°)	0.93	0.93	0.93	0.92	0.93

[a] Diffraction Weighted Dose as described by Zeldin *et al.*
[b] Values in parentheses are for the highest resolution shell.
[c] Half-dataset correlation coefficient, see: Karplus, P. A.; Diederichs, K. Linking crystallographic model and data quality. *Science* **2012**, *336*, 1030−1033.

Sweep	Sweep 6	Sweep 7	Sweep 8	Sweep 9	Sweep 10
**PDB Code**	**7GVR**	**7GVS**	**7GVT**	**7GVU**	**7GVV**
Dose (MGy)[a]	8.43	9.83	11.23	12.64	14.04
Space group	$P6_122$	$P6_122$	$P6_122$	$P6_122$	$P6_122$
Unit Cell (a=b,c) (Å)	67.66, 166.97	67.81, 167.13	67.65, 166.97	67.82, 167.19	67.68, 167.01
Unit Cell (α=β,γ) (°)	90.00, 120.00	90.00, 120.00	90.00, 120.00	90.00, 120.00	90.00, 120.00
Beamline	DLS I24	DLS I24	DLS I24	DLS I24	DLS I24
Wavelength (Å)	0.9686	0.9686	0.9686	0.9686	0.9686
Resolution (Å) [b]	34.00-1.90 (1.94-1.90)	34.04-1.90 (1.94-1.90)	34.00-1.90 (1.94-1.90)	34.05-1.90 (1.94-1.90)	34.01-1.90 (1.94-1.90)
Unique Reflections [b]	18695 (1169)	18797 (1179)	18685 (1167)	18806 (1173)	18710 (1172)
Multiplicity [b]	9.0 (9.4)	9.0 (9.4)	9.0 (9.3)	9.0 (9.4)	9.0 (9.4)
$R_{p.i.m}$ (%) [b]	2.5 (27.0)	2.2 (25.7)	2.8 (32.6)	2.3 (32.6)	3.0 (41.3)
R_{meas} (%) [b]	7.6 (83.5)	6.6 (79.6)	8.4 (100.0)	7.0 (100.7)	9.1 (127.5)
CC1/2 [b,c]	0.999 (0.852)	1.000 (0.861)	0.999 (0.811)	1.000 (0.823)	0.999 (0.755)
$I / \sigma(I)$ [b]	14.3 (2.5)	16.9 (2.7)	13.2 (2.1)	15.5 (2.1)	12.0 (1.6)
Completeness (%) [b]	100.0 (100.0)	100.0 (100.0)	100.0 (100.0)	100.0 (100.0)	100.0 (100.0)
Wilson B (Å^2)	29.2	29.3	28.6	30.9	32.0
**Refinement**					
R_{work} / R_{free}	19.16 / 21.26	19.16 / 20.98	18.98 / 21.15	19.20 / 21.34	19.06 / 21.27
No. Atoms					
Protein	1078	1078	1078	1078	1078
Ligand/ion	27	27	27	27	27
Water	138	138	138	138	138
Ramachandran (#,%)					
Allowed	120 (98.36%)	119 (97.54%)	119 (97.54%)	119 (97.54%)	119 (97.54%)
Generally allowed	2 (1.64%)	3 (2.46%)	3 (2.46%)	3 (2.46%)	3 (2.46%)
Disallowed	0 (0.00%)	0 (0.00%)	0 (0.00%)	0 (0.00%)	0 (0.00%)
B-factors					
Protein	39.22	39.58	39.41	41.34	41.06
Ligand/ion	35.70	36.46	36.58	38.45	37.66
Water	55.76	56.03	56.43	58.38	58.88
R.M.S. deviations					
Bond lengths (Å)	0.01	0.01	0.01	0.01	0.01
Bond angles (°)	0.93	0.94	0.93	0.94	0.93

[a] Diffraction Weighted Dose as described by Zeldin *et al.*

[b] Values in parentheses are for the highest resolution shell.

[c] Half-dataset correlation coefficient, see: Karplus, P. A.; Diederichs, K. Linking crystallographic model and data quality. *Science* **2012**, *336*, 1030–1033.

Sweep	Sweep 11	Sweep 12	Sweep 13	Sweep 14	Sweep 15
**PDB Code**	**7GVW**	**7GVX**	**7GVY**	**7GVZ**	**7GW0**
Dose (MGy)[a]	15.45	16.85	18.26	19.66	21.06
Space group	$P6_122$	$P6_122$	$P6_122$	$P6_122$	$P6_122$
Unit Cell (a=b,c) (Å)	67.84, 167.20	67.64, 167.01	67.83, 167.27	67.68, 167.02	67.86, 167.23
Unit Cell (α=β,γ) (°)	90.00, 120.00	90.00, 120.00	90.00, 120.00	90.00, 120.00	90.00, 120.00
Beamline	DLS I24	DLS I24	DLS I24	DLS I24	DLS I24
Wavelength (Å)	0.9686	0.9686	0.9686	0.9686	0.9686
Resolution (Å) [b]	34.06-1.90 (1.94-1.90)	34.00-1.90 (1.94-1.90)	34.07-1.90 (1.94-1.90)	34.01-1.90 (1.94-1.90)	34.07-1.90 (1.94-1.90)
Unique Reflections [b]	18816 (1173)	18688 (1168)	18819 (1171)	18707 (1170)	18824 (1170)
Multiplicity [b]	9.0 (9.4)	9.0 (9.3)	9.0 (9.4)	9.0 (9.3)	9.0 (9.4)
$R_{p.i.m}$ (%) [b]	2.5 (37.1)	3.3 (48.2)	2.7 (43.5)	3.6 (55.0)	3.0 (48.3)
R_{meas} (%) [b]	7.7 (114.6)	10.1 (148.7)	8.1 (134.1)	10.9 (169.5)	8.9 (149.4)
CC1/2 [b,c]	0.999 (0.775)	0.999 (0.725)	0.999 (0.742)	0.999 (0.600)	0.999 (0.752)
$I / \sigma(I)$ [b]	14.3 (1.9)	10.7 (1.4)	13.1 (1.6)	9.8 (1.3)	12.0 (1.4)
Completeness (%) [b]	100.0 (100.0)	100.0 (100.0)	100.0 (100.0)	100.0 (100.0)	100.0 (100.0)
Wilson B (Å^2)	32.0	32.0	33.5	34.7	33.8
**Refinement**					
R_{work} / R_{free}	19.22 / 21.40	18.83 / 20.93	19.08 / 21.33	18.95 / 21.29	19.31 / 21.18
No. Atoms					
Protein	1078	1078	1078	1078	1078
Ligand/ion	27	27	27	27	27
Water	138	138	138	138	138
Ramachandran (#,%)					
Allowed	119 (97.54%)	119 (97.54%)	119 (97.54%)	119 (97.54%)	119 (97.54%)
Generally allowed	3 (2.46%)	3 (2.46%)	3 (2.46%)	3 (2.46%)	3 (2.46%)
Disallowed	0 (0.00%)	0 (0.00%)	0 (0.00%)	0 (0.00%)	0 (0.00%)
B-factors					
Protein	41.49	40.90	42.86	42.72	42.77
Ligand/ion	38.43	37.67	40.08	39.52	39.92
Water	59.12	58.59	60.98	60.42	60.43
R.M.S. deviations					
Bond lengths (Å)	0.01	0.01	0.01	0.01	0.01
Bond angles (°)	0.95	0.94	0.94	0.94	0.96

[a] Diffraction Weighted Dose as described by Zeldin *et al.*
[b] Values in parentheses are for the highest resolution shell.
[c] Half-dataset correlation coefficient, see: Karplus, P. A.; Diederichs, K. Linking crystallographic model and data quality. *Science* **2012**, *336*, 1030–1033.

S2.5. BCL6 ligand 5

Sweep	Sweep 1	Sweep 2	Sweep 3	Sweep 4	Sweep 5
**PDB Code**	**7GW1**	**7GW2**	**7GW3**	**7GW4**	**7GW5**
Dose (MGy)[a]	1.29	2.58	3.87	5.16	6.45
Space group	$P6_122$	$P6_122$	$P6_122$	$P6_122$	$P6_122$
Unit Cell (a=b,c) (Å)	67.49, 166.30	67.49, 166.03	67.53, 166.30	67.54, 166.15	67.62, 166.59
Unit Cell (α=β,γ) (°)	90.00, 120.00	90.00, 120.00	90.00, 120.00	90.00, 120.00	90.00, 120.00
Beamline	DLS I24	DLS I24	DLS I24	DLS I24	DLS I24
Wavelength (Å)	0.9688	0.9688	0.9688	0.9688	0.9688
Resolution (Å)[b]	33.88-1.75 (1.78-1.75)	33.84-1.75 (1.78-1.75)	33.89-1.75 (1.78-1.75)	33.87-1.75 (1.78-1.75)	33.94-1.75 (1.78-1.75)
Unique Reflections[b]	23337 (1268)	23496 (1265)	23348 (1270)	23558 (1275)	23470 (1284)
Multiplicity[b]	9.2 (9.3)	9.1 (9.4)	9.2 (9.3)	9.1 (9.3)	9.1 (9.3)
$R_{p.i.m}$ (%)[b]	2.7 (8.8)	2.4 (8.0)	2.9 (10.5)	3.0 (10.7)	2.6 (14.2)
R_{meas} (%)[b]	8.5 (26.9)	7.3 (25.1)	8.8 (32.2)	9.2 (33.6)	8.0 (43.5)
CC1/2[b,c]	0.997 (0.97)	0.997 (0.975)	0.996 (0.961)	0.996 (0.962)	0.998 (0.924)
I / σ(I)	15.8 (6.7)	18.2 (7.4)	14.9 (5.9)	14.4 (5.6)	16.1 (4.2)
Completeness (%)[b]	99.7 (100.0)	100.0 (100.0)	99.7 (100.0)	100.0 (100.0)	99.7 (100.0)
Wilson B ($Å^2$)	17.9	17.8	18.7	19.5	19.6
**Refinement**					
R_{work} / R_{free}	17.00 / 19.12	16.87 / 18.80	16.86 / 18.98	16.97 / 18.94	17.07 / 19.12
No. Atoms					
Protein	1190	1190	1190	1190	1190
Ligand/ion	27	27	27	27	27
Water	152	152	152	152	152
Ramachandran (#,%)					
Allowed	119 (95.97%)	119 (95.97%)	120 (96.77%)	119 (95.97%)	119 (95.97%)
Generally allowed	5 (4.03%)	5 (4.03%)	4 (3.23%)	5 (4.03%)	5 (4.03%)
Disallowed	0 (0.00%)	0 (0.00%)	0 (0.00%)	0 (0.00%)	0 (0.00%)
B-factors					
Protein	25.02	25.15	25.79	26.98	27.97
Ligand/ion	24.93	25.08	25.93	26.62	28.10
Water	42.74	43.36	44.75	46.25	48.03
R.M.S. deviations					
Bond lengths (Å)	0.01	0.01	0.01	0.01	0.01
Bond angles (°)	0.89	0.90	0.90	0.90	0.89

[a] Diffraction Weighted Dose as described by Zeldin *et al.*
[b] Values in parentheses are for the highest resolution shell.
[c] Half-dataset correlation coefficient, see: Karplus, P. A.; Diederichs, K. Linking crystallographic model and data quality. *Science* **2012**, *336*, 1030–1033.

Sweep	Sweep 6	Sweep 7	Sweep 8	Sweep 9	Sweep 10
**PDB Code**	**7GW6**	**7GW7**	**7GW8**	**7GW9**	**7GWA**
Dose (MGy)[a]	7.74	9.03	10.32	11.61	12.90
Space group	$P6_122$	$P6_122$	$P6_122$	$P6_122$	$P6_122$
Unit Cell (a=b,c) (Å)	67.58, 166.16	67.63, 166.43	67.64, 166.28	67.69, 166.71	67.69, 166.37
Unit Cell (α=β,γ) (°)	90.00, 120.00	90.00, 120.00	90.00, 120.00	90.00, 120.00	90.00, 120.00
Beamline	DLS I24	DLS I24	DLS I24	DLS I24	DLS I24
Wavelength (Å)	0.9683	0.9688	0.9688	0.9688	0.9688
Resolution (Å) [b]	33.87-1.75 (1.78-1.75)	33.92-1.75 (1.78-1.75)	33.90-1.75 (1.78-1.75)	33.97-1.75 (1.78-1.75)	33.92-1.75 (1.78-1.75)
Unique Reflections [b]	23581 (1276)	23438 (1284)	23653 (1285)	23518 (1323)	23682 (1282)
Multiplicity [b]	9.1 (9.3)	9.2 (9.4)	9.1 (9.2)	9.1 (9.3)	9.0 (9.2)
$R_{p.i.m}$ (%) [b]	2.3 (13.8)	2.7 (19.3)	2.8 (20.5)	2.9 (29.9)	2.6 (30.7)
R_{meas} (%) [b]	6.8 (43.6)	8.1 (59.1)	8.5 (64.4)	8.9 (90.5)	7.8 (96.5)
CC1/2 [b,c]	0.998 (0.933)	0.998 (0.897)	0.998 (0.885)	0.998 (0.761)	0.998 (0.793)
$I / \sigma(I)$	17.4 (4.5)	14.7 (3.8)	14.1 (3.2)	13.8 (2.2)	14.3 (2.2)
Completeness (%) [b]	100.0 (100.0)	99.7 (100.0)	100.0 (100.0)	99.7 (100.0)	100.0 (100.0)
Wilson B ($Å^2$)	20.3	20.9	21.5	21.8	22.8
**Refinement**					
R_{work} / R_{free}	16.99 / 19.06	16.77 / 18.70	17.24 / 19.37	17.25 / 19.19	17.42 / 19.26
No. Atoms					
Protein	1190	1190	1190	1190	1190
Ligand/ion	27	27	27	27	27
Water	152	152	152	152	152
Ramachandran (#,%)					
Allowed	119 (95.97%)	119 (95.97%)	119 (95.97%)	119 (95.97%)	119 (95.97%)
Generally allowed	5 (4.03%)	5 (4.03%)	5 (4.03%)	5 (4.03%)	5 (4.03%)
Disallowed	0 (0.00%)	0 (0.00%)	0 (0.00%)	0 (0.00%)	0 (0.00%)
B-factors					
Protein	28.63	29.25	30.94	31.57	32.64
Ligand/ion	28.62	29.23	30.98	31.67	32.53
Water	49.16	50.51	52.56	53.94	55.12
R.M.S. deviations					
Bond lengths (Å)	0.01	0.01	0.01	0.01	0.01
Bond angles (°)	0.90	0.90	0.90	0.89	0.90

[a] Diffraction Weighted Dose as described by Zeldin *et al.*

[b] Values in parentheses are for the highest resolution shell.

[c] Half-dataset correlation coefficient, see: Karplus, P. A.; Diederichs, K. Linking crystallographic model and data quality. *Science* **2012**, *336*, 1030–1033.

Sweep	Sweep 11	Sweep 12	Sweep 13	Sweep 14	Sweep 15
**PDB Code**	**7GWB**	**7GWC**	**7GWD**	**7GWE**	**7GWF**
Dose (MGy)[a]	14.19	15.48	16.77	18.06	19.35
Space group	$P6_122$	$P6_122$	$P6_122$	$P6_122$	$P6_122$
Unit Cell (a=b,c) (Å)	67.68, 166.44	67.71, 166.35	67.75, 166.78	67.76, 166.49	67.73, 166.46
Unit Cell (α=β,γ) (°)	90.00, 120.00	90.00, 120.00	90.00, 120.00	90.00, 120.00	90.00, 120.00
Beamline	DLS I24	DLS I24	DLS I24	DLS I24	DLS I24
Wavelength (Å)	0.9688	0.9688	0.9688	0.9688	0.9688
Resolution (Å) [b]	33.93-1.75 (1.78-1.75)	33.92-1.75 (1.78-1.75)	33.99-1.75 (1.78-1.75)	33.95-1.75 (1.78-1.75)	33.94-1.75 (1.78-1.75)
Unique Reflections [b]	23467 (1286)	23694 (1278)	23568 (1297)	23750 (1335)	23505 (1290)
Multiplicity [b]	9.1 (9.3)	9.0 (9.2)	9.1 (9.2)	9.0 (9.2)	9.1 (9.3)
$R_{p.i.m}$ (%)	3.0 (42.8)	3.2 (47.1)	3.5 (74.7)	3.2 (71.2)	3.6 (103.2)
R_{meas} (%) [b]	9.0 (130.1)	9.7 (148.5)	10.5 (223.0)	9.7 (225.1)	11.0 (310.5)
CC1/2 [b,c]	0.998 (0.687)	0.998 (0.653)	0.998 (0.433)	0.998 (0.49)	0.998 (0.381)
$I / \sigma(I)$ [b]	12.6 (1.9)	11.4 (1.6)	10.5 (1.2)	11.6 (1.0)	10.3 (1.0)
Completeness (%) [b]	99.7 (100.0)	100.0 (100.0)	99.7 (100.0)	100.0 (100.0)	99.7 (100.0)
Wilson B (Å^2)	23.7	25.0	26.3	27.4	29.9
**Refinement**					
R_{work} / R_{free}	17.24 / 19.15	17.70 / 19.61	17.89 / 19.89	17.92 / 19.94	17.71 / 19.72
No. Atoms					
Protein	1190	1190	1190	1190	1190
Ligand/ion	27	27	27	27	27
Water	152	152	152	152	152
Ramachandran (#,%)					
Allowed	119 (95.97%)	120 (96.77%)	119 (95.97%)	120 (96.77%)	120 (96.77%)
Generally allowed	5 (4.03%)	4 (3.23%)	5 (4.03%)	4 (3.23%)	4 (3.23%)
Disallowed	0 (0.00%)	0 (0.00%)	0 (0.00%)	0 (0.00%)	0 (0.00%)
B-factors					
Protein	33.08	34.84	35.43	36.24	36.86
Ligand/ion	33.20	34.73	35.88	35.85	37.55
Water	56.36	57.30	58.96	60.12	59.98
R.M.S. deviations					
Bond lengths (Å)	0.01	0.01	0.01	0.01	0.01
Bond angles (°)	0.90	0.89	0.89	0.89	0.89

[a] Diffraction Weighted Dose as described by Zeldin *et al.*

[b] Values in parentheses are for the highest resolution shell.

[c] Half-dataset correlation coefficient, see: Karplus, P. A.; Diederichs, K. Linking crystallographic model and data quality. *Science* **2012**, *336*, 1030–1033.

S2.6. BCL6 ligand 6

Sweep	Sweep 1	Sweep 2	Sweep 3	Sweep 4	Sweep 5
**PDB Code**	**7GWG**	**7GWH**	**7GWI**	**7GWJ**	**7GWK**
Dose (MGy)[a]	1.23	2.46	3.70	4.93	6.16
Space group	$P6_122$	$P6_122$	$P6_122$	$P6_122$	$P6_122$
Unit Cell (a=b,c) (Å)	67.74, 166.87	67.60, 166.82	67.77, 167.02	67.63, 166.89	67.79, 167.09
Unit Cell (α=β,γ) (°)	90.00, 120.00	90.00, 120.00	90.00, 120.00	90.00, 120.00	90.00, 120.00
Beamline	DLS I24	DLS I24	DLS I24	DLS I24	DLS I24
Wavelength (Å)	0.9686	0.9686	0.9686	0.9686	0.9686
Resolution (Å) [b]	34.00-1.90 (1.94-1.90)	33.97-1.90 (1.94-1.90)	34.02-1.90 (1.94-1.90)	33.98-1.90 (1.94-1.90)	34.04-1.90 (1.94-1.90)
Unique Reflections [b]	18736 (1180)	18655 (1175)	18760 (1177)	18670 (1173)	18782 (1171)
Multiplicity [b]	9.0 (9.4)	9.1 (9.4)	9.0 (9.4)	9.1 (9.4)	9.0 (9.4)
$R_{p.i.m}$ (%) [b]	2.0 (14.8)	2.1 (16.1)	1.9 (16.9)	2.4 (20.5)	2.1 (22.6)
R_{meas} (%) [b]	6.0 (45.9)	6.5 (49.7)	5.9 (52.2)	7.1 (63.3)	6.4 (70.1)
CC1/2 [b,c]	1.000 (0.941)	0.999 (0.936)	1.0 (0.933)	0.999 (0.917)	1.0 (0.889)
$I / \sigma(I)$ [b]	18.5 (4.4)	16.9 (4.0)	19.1 (4.1)	15.7 (3.0)	17.1 (3.0)
Completeness (%) [b]	100.0 (100.0)	100.0 (100.0)	100.0 (100.0)	100.0 (100.0)	100.0 (100.0)
Wilson B ($Å^2$)	26.2	26.1	26.7	26.4	28.0
**Refinement**					
R_{work} / R_{free}	19.62 / 22.33	19.27 / 21.74	19.18 / 20.87	19.22 / 21.25	19.24 / 21.15
No. Atoms					
Protein	1078	1078	1078	1078	1078
Ligand/ion	27	27	27	27	27
Water	138	138	138	138	138
Ramachandran (#,%)					
Allowed	120 (98.36%)	119 (97.54%)	119 (97.54%)	120 (98.36%)	120 (98.36%)
Generally allowed	2 (1.64%)	3 (2.46%)	3 (2.46%)	2 (1.64%)	2 (1.64%)
Disallowed	0 (0.00%)	0 (0.00%)	0 (0.00%)	0 (0.00%)	0 (0.00%)
B-factors					
Protein	36.12	36.01	37.05	37.16	39.07
Ligand/ion	32.80	32.54	33.87	33.85	35.99
Water	49.89	50.66	51.95	52.51	54.74
R.M.S. deviations					
Bond lengths (Å)	0.01	0.01	0.01	0.01	0.01
Bond angles (°)	0.93	0.93	0.93	0.92	0.93

[a] Diffraction Weighted Dose as described by Zeldin *et al.*

[b] Values in parentheses are for the highest resolution shell.

[c] Half-dataset correlation coefficient, see: Karplus, P. A.; Diederichs, K. Linking crystallographic model and data quality. *Science* **2012**, *336*, 1030–1033.

Sweep	Sweep 6	Sweep 7	Sweep 8	Sweep 9	Sweep 10
**PDB Code**	**7GWL**	**7GWM**	**7GWN**	**7GWO**	**7GWP**
Dose (MGy)[a]	7.39	8.62	9.86	11.09	12.32
Space group	$P6_122$	$P6_122$	$P6_122$	$P6_122$	$P6_122$
Unit Cell (a=b,c) (Å)	67.66, 166.97	67.81, 167.13	67.65, 166.97	67.82, 167.19	67.68, 167.01
Unit Cell (α=β,γ) (°)	90.00, 120.00	90.00, 120.00	90.00, 120.00	90.00, 120.00	90.00, 120.00
Beamline	DLS I24	DLS I24	DLS I24	DLS I24	DLS I24
Wavelength (Å)	0.9686	0.9686	0.9686	0.9686	0.9686
Resolution (Å) [b]	34.00-1.90 (1.94-1.90)	34.04-1.90 (1.94-1.90)	34.00-1.90 (1.94-1.90)	34.05-1.90 (1.94-1.90)	34.01-1.90 (1.94-1.90)
Unique Reflections [b]	18695 (1169)	18797 (1179)	18685 (1167)	18806 (1173)	18710 (1172)
Multiplicity [b]	9.0 (9.4)	9.0 (9.4)	9.0 (9.3)	9.0 (9.4)	9.0 (9.4)
$R_{p.i.m}$ (%) [b]	2.5 (27.0)	2.2 (25.7)	2.8 (32.6)	2.3 (32.6)	3.0 (41.3)
R_{meas} (%) [b]	7.6 (83.5)	6.6 (79.6)	8.4 (100.0)	7.0 (100.7)	9.1 (127.5)
CC1/2 [b,c]	0.999 (0.852)	1.000 (0.861)	0.999 (0.811)	1.000 (0.823)	0.999 (0.755)
$I / \sigma(I)$ [b]	14.3 (2.5)	16.9 (2.7)	13.2 (2.1)	15.5 (2.1)	12.0 (1.6)
Completeness (%) [b]	100.0 (100.0)	100.0 (100.0)	100.0 (100.0)	100.0 (100.0)	100.0 (100.0)
Wilson B (Å^2)	29.2	29.3	28.6	30.9	32.0
**Refinement**					
R_{work} / R_{free}	19.16 / 21.26	19.16 / 20.98	18.98 / 21.15	19.20 / 21.34	19.06 / 21.27
No. Atoms					
Protein	1078	1078	1078	1078	1078
Ligand/ion	27	27	27	27	27
Water	138	138	138	138	138
Ramachandran (#,%)					
Allowed	120 (98.36%)	119 (97.54%)	119 (97.54%)	119 (97.54%)	119 (97.54%)
Generally allowed	2 (1.64%)	3 (2.46%)	3 (2.46%)	3 (2.46%)	3 (2.46%)
Disallowed	0 (0.00%)	0 (0.00%)	0 (0.00%)	0 (0.00%)	0 (0.00%)
B-factors					
Protein	39.22	39.58	39.41	41.34	41.06
Ligand/ion	35.70	36.46	36.58	38.45	37.66
Water	55.76	56.03	56.43	58.38	58.88
R.M.S. deviations					
Bond lengths (Å)	0.01	0.01	0.01	0.01	0.01
Bond angles (°)	0.93	0.94	0.93	0.94	0.93

[a] Diffraction Weighted Dose as described by Zeldin *et al.*

[b] Values in parentheses are for the highest resolution shell.

[c] Half-dataset correlation coefficient, see: Karplus, P. A.; Diederichs, K. Linking crystallographic model and data quality. *Science* **2012**, *336*, 1030–1033.

Sweep	Sweep 11	Sweep 12	Sweep 13	Sweep 14	Sweep 15
**PDB Code**	**7GWQ**	**7GWR**	**7GWS**	**7GWT**	**7GWU**
Dose (MGy)[a]	13.55	14.78	16.01	17.25	18.48
Space group	$P6_122$	$P6_122$	$P6_122$	$P6_122$	$P6_122$
Unit Cell (a=b,c) (Å)	67.84, 167.20	67.64, 167.01	67.83, 167.27	67.68, 167.02	67.86, 167.23
Unit Cell (α=β,γ) (°)	90.00, 120.00	90.00, 120.00	90.00, 120.00	90.00, 120.00	90.00, 120.00
Beamline	DLS I24	DLS I24	DLS I24	DLS I24	DLS I24
Wavelength (Å)	0.9686	0.9686	0.9686	0.9686	0.9686
Resolution (Å) [b]	34.06-1.90 (1.94-1.90)	34.00-1.90 (1.94-1.90)	34.07-1.90 (1.94-1.90)	34.01-1.90 (1.94-1.90)	34.07-1.90 (1.94-1.90)
Unique Reflections [b]	18816 (1173)	18688 (1168)	18819 (1171)	18707 (1170)	18824 (1170)
Multiplicity [b]	9.0 (9.4)	9.0 (9.3)	9.0 (9.4)	9.0 (9.3)	9.0 (9.4)
$R_{p.i.m}$ (%) [b]	2.5 (37.1)	3.3 (48.2)	2.7 (43.5)	3.6 (55.0)	3.0 (48.3)
R_{meas} (%) [b]	7.7 (114.6)	10.1 (148.7)	8.1 (134.1)	10.9 (169.5)	8.9 (149.4)
CC1/2 [b,c]	0.999 (0.775)	0.999 (0.725)	0.999 (0.742)	0.999 (0.600)	0.999 (0.752)
I / σ(I) [b]	14.3 (1.9)	10.7 (1.4)	13.1 (1.6)	9.8 (1.3)	12.0 (1.4)
Completeness (%) [b]	100.0 (100.0)	100.0 (100.0)	100.0 (100.0)	100.0 (100.0)	100.0 (100.0)
Wilson B (Å^2)	32.0	32.0	33.5	34.7	33.8
**Refinement**					
R_{work} / R_{free}	19.22 / 21.40	18.83 / 20.93	19.08 / 21.33	18.95 / 21.29	19.31 / 21.18
No. Atoms					
Protein	1078	1078	1078	1078	1078
Ligand/ion	27	27	27	27	27
Water	138	138	138	138	138
Ramachandran (#,%)					
Allowed	119 (97.54%)	119 (97.54%)	119 (97.54%)	119 (97.54%)	119 (97.54%)
Generally allowed	3 (2.46%)	3 (2.46%)	3 (2.46%)	3 (2.46%)	3 (2.46%)
Disallowed	0 (0.00%)	0 (0.00%)	0 (0.00%)	0 (0.00%)	0 (0.00%)
B-factors					
Protein	41.49	40.90	42.86	42.72	42.77
Ligand/ion	38.43	37.67	40.08	39.52	39.92
Water	59.12	58.59	60.98	60.42	60.43
R.M.S. deviations					
Bond lengths (Å)	0.01	0.01	0.01	0.01	0.01
Bond angles (°)	0.95	0.94	0.94	0.94	0.96

[a] Diffraction Weighted Dose as described by Zeldin *et al.*

[b] Values in parentheses are for the highest resolution shell.

[c] Half-dataset correlation coefficient, see: Karplus, P. A.; Diederichs, K. Linking crystallographic model and data quality. *Science* **2012**, *336*, 1030–1033.

S2.7. BCL6 ligand 7

Sweep	Sweep 1	Sweep 2	Sweep 3	Sweep 4	Sweep 5
**PDB Code**	**7GWV**	**7GWW**	**7GWX**	**7GWY**	**7GWZ**
Dose (MGy)[a]	1.45	2.90	4.35	5.80	7.25
Space group	$P6_122$	$P6_122$	$P6_122$	$P6_122$	$P6_122$
Unit Cell (a=b,c) (Å)	67.35, 165.67	67.37, 165.72	67.39, 165.76	67.40, 165.81	67.39, 165.75
Unit Cell (α=β,γ) (°)	90.00, 120.00	90.00, 120.00	90.00, 120.00	90.00, 120.00	90.00, 120.00
Beamline	DLS I24	DLS I24	DLS I24	DLS I24	DLS I24
Wavelength (Å)	0.9686	0.9686	0.9686	0.9686	0.9686
Resolution (Å) [b]	33.77-1.70 (1.73-1.70)	33.78-1.70 (1.73-1.70)	33.79-1.70 (1.73-1.70)	33.80-1.70 (1.73-1.70)	33.79-1.70 (1.73-1.70)
Unique Reflections [b]	25384 (1316)	25428 (1315)	25417 (1323)	25464 (1325)	25415 (1321)
Multiplicity [b]	9.1 (9.3)	9.1 (9.3)	9.1 (9.3)	9.1 (9.3)	9.1 (9.3)
$R_{p.i.m}$ (%) [b]	2.0 (23.5)	2.0 (35.5)	2.0 (31.8)	2.0 (29.5)	2.5 (38.6)
R_{meas} (%) [b]	6.0 (73.5)	6.0 (108.2)	6.1 (97.8)	6.1 (90.6)	7.5 (120.1)
CC1/2 [b,c]	0.999 (0.935)	0.999 (0.861)	1.000 (0.873)	1.000 (0.918)	0.999 (0.853)
$I / \sigma(I)$ [b]	21.8 (3.4)	21.7 (2.4)	20.8 (2.0)	19.1 (2.3)	15.4 (1.8)
Completeness (%) [b]	100.0 (100.0)	100.0 (100.0)	100.0 (100.0)	100.0 (100.0)	100.0 (100.0)
Wilson B (Å^2)	14.4	14.1	15.0	15.7	17.2
**Refinement**					
R_{work} / R_{free}	18.82 / 20.97	19.23 / 21.67	18.90 / 21.23	18.71 / 21.01	18.16 / 20.25
No. Atoms					
Protein	1126	1126	1126	1126	1126
Ligand/ion	29	29	29	29	29
Water	208	208	208	208	208
Ramachandran (#,%)					
Allowed	119 (95.97%)	119 (95.97%)	119 (95.97%)	119 (95.97%)	119 (95.97%)
Generally allowed	5 (4.03%)	5 (4.03%)	5 (4.03%)	5 (4.03%)	5 (4.03%)
Disallowed	0 (0.00%)	0 (0.00%)	0 (0.00%)	0 (0.00%)	0 (0.00%)
B-factors					
Protein	21.67	22.72	23.88	24.46	26.13
Ligand/ion	23.94	25.06	26.47	26.94	28.81
Water	39.31	41.11	43.39	43.90	46.66
R.M.S. deviations					
Bond lengths (Å)	0.01	0.01	0.01	0.01	0.01
Bond angles (°)	0.89	0.90	0.89	0.89	0.89

[a] Diffraction Weighted Dose as described by Zeldin *et al.*

[b] Values in parentheses are for the highest resolution shell.

[c] Half-dataset correlation coefficient, see: Karplus, P. A.; Diederichs, K. Linking crystallographic model and data quality. *Science* **2012**, *336*, 1030–1033.

Sweep	Sweep 6	Sweep 7	Sweep 8	Sweep 9	Sweep 10
**PDB Code**	**7GX0**	**7GX1**	**7GX2**	**7GX3**	**7GX4**
Dose (MGy)[a]	8.70	10.14	11.59	13.04	14.49
Space group	$P6_122$	$P6_122$	$P6_122$	$P6_122$	$P6_122$
Unit Cell (a=b,c) (Å)	67.41, 165.88	67.37, 165.72	67.44, 165.85	67.35, 165.58	67.42, 165.89
Unit Cell (α=β,γ) (°)	90.00, 120.00	90.00, 120.00	90.00, 120.00	90.00, 120.00	90.00, 120.00
Beamline	DLS I24	DLS I24	DLS I24	DLS I24	DLS I24
Wavelength (Å)	0.9686	0.9686	0.9686	0.9686	0.9686
Resolution (Å) [b]	33.81-1.70 (1.73-1.70)	33.78-1.70 (1.73-1.70)	33.81-1.70 (1.73-1.70)	33.76-1.70 (1.73-1.70)	33.81-1.70 (1.73-1.70)
Unique Reflections [b]	25492 (1325)	25394 (1314)	25503 (1326)	25367 (1313)	25496 (1329)
Multiplicity [b]	9.1 (9.3)	9.1 (9.3)	9.1 (9.3)	9.1 (9.3)	9.1 (9.3)
$R_{p.i.m}$ (%) [b]	2.6 (56.7)	2.7 (49.0)	2.7 (47.5)	3.8 (55.8)	3.5 (83.4)
R_{meas} (%) [b]	7.8 (174.0)	8.3 (151.8)	8.3 (146.2)	11.7 (171.8)	10.6 (255.9)
CC1/2 [b,c]	0.999 (0.693)	0.999 (0.734)	0.999 (0.825)	0.998 (0.753)	0.999 (0.581)
$I / \sigma(I)$ [b]	14.4 (1.3)	12.5 (1.4)	12.8 (1.4)	8.7 (1.3)	9.4 (0.9)
Completeness (%) [b]	100.0 (100.0)	100.0 (100.0)	100.0 (100.0)	100.0 (100.0)	100.0 (100.0)
Wilson B (Å^2)	17.6	19.3	19.2	22.2	21.9
**Refinement**					
R_{work} / R_{free}	18.73 / 20.91	18.41 / 20.80	18.31 / 20.66	17.52 / 19.98	18.28 / 20.92
No. Atoms					
Protein	1126	1126	1126	1126	1126
Ligand/ion	29	29	29	29	29
Water	208	208	208	208	208
Ramachandran (#,%)					
Allowed	119 (95.97%)	120 (96.77%)	120 (96.77%)	121 (97.58%)	119 (95.97%)
Generally allowed	5 (4.03%)	4 (3.23%)	4 (3.23%)	3 (2.42%)	5 (4.03%)
Disallowed	0 (0.00%)	0 (0.00%)	0 (0.00%)	0 (0.00%)	0 (0.00%)
B-factors					
Protein	27.03	28.17	27.97	28.42	29.86
Ligand/ion	30.08	31.38	30.80	31.56	32.94
Water	47.92	49.62	49.17	50.22	51.67
R.M.S. deviations					
Bond lengths (Å)	0.01	0.01	0.01	0.01	0.01
Bond angles (°)	0.90	0.89	0.89	0.88	0.89

[a] Diffraction Weighted Dose as described by Zeldin *et al.*

[b] Values in parentheses are for the highest resolution shell.

[c] Half-dataset correlation coefficient, see: Karplus, P. A.; Diederichs, K. Linking crystallographic model and data quality. *Science* **2012**, *336*, 1030–1033.

Sweep	Sweep 11	Sweep 12	Sweep 13	Sweep 14	Sweep 15
**PDB Code**	**7GX5**	**7GX6**	**7GX7**	**7GX8**	**7GX9**
Dose (MGy)[a]	15.94	17.39	18.84	20.29	21.74
Space group	$P6_122$	$P6_122$	$P6_122$	$P6_122$	$P6_122$
Unit Cell (a=b,c) (Å)	67.34, 165.58	67.46, 165.86	67.33, 165.49	67.41, 165.84	67.32, 165.51
Unit Cell (α=β,γ) (°)	90.00, 120.00	90.00, 120.00	90.00, 120.00	90.00, 120.00	90.00, 120.00
Beamline	DLS I24	DLS I24	DLS I24	DLS I24	DLS I24
Wavelength (Å)	0.9686	0.9686	0.9686	0.9686	0.9686
Resolution (Å) [b]	33.76-1.70 (1.73-1.70)	33.81-1.70 (1.73-1.70)	33.74-1.70 (1.73-1.70)	33.80-1.70 (1.73-1.70)	33.74-1.70 (1.73-1.70)
Unique Reflections [b]	25348 (1311)	25520 (1324)	25334 (1309)	25485 (1328)	25323 (1303)
Multiplicity [b]	9.1 (9.3)	9.1 (9.3)	9.1 (9.3)	9.1 (9.3)	9.1 (9.3)
$R_{p.i.m}$ (%) [b]	4.6 (70.4)	4.0 (77.1)	5.5 (74.9)	4.9 (122.4)	5.6 (87.7)
R_{meas} (%) [b]	14.0 (218.7)	12.2 (235.7)	16.7 (231.0)	14.9 (375.5)	17.0 (273.5)
CC1/2 [b,c]	0.995 (0.505)	0.997 (0.637)	0.992 (0.646)	0.996 (0.427)	0.991 (0.442)
$I / \sigma(I)$ [b]	7.7 (1.0)	8.3 (1.0)	7.0 (1.1)	7.0 (0.7)	6.7 (0.8)
Completeness (%) [b]	100.0 (100.0)	100.0 (100.0)	100.0 (100.0)	100.0 (100.0)	100.0 (100.0)
Wilson B ($Å^2$)	23.1	23.2	23.5	25.1	25.7
**Refinement**					
R_{work} / R_{free}	17.92 / 20.61	17.97 / 20.69	17.44 / 19.96	18.06 / 20.89	17.74 / 20.45
No. Atoms					
Protein	1126	1126	1126	1126	1126
Ligand/ion	29	29	29	29	29
Water	208	208	208	208	208
Ramachandran (#,%)					
Allowed	120 (96.77%)	121 (97.58%)	121 (97.58%)	119 (95.97%)	119 (95.97%)
Generally allowed	4 (3.23%)	3 (2.42%)	3 (2.42%)	5 (4.03%)	5 (4.03%)
Disallowed	0 (0.00%)	0 (0.00%)	0 (0.00%)	0 (0.00%)	0 (0.00%)
B-factors					
Protein	29.02	29.95	28.40	30.69	29.30
Ligand/ion	32.48	32.58	31.51	33.83	32.58
Water	51.27	51.69	50.60	53.41	51.70
R.M.S. deviations					
Bond lengths (Å)	0.01	0.01	0.01	0.01	0.01
Bond angles (°)	0.88	0.88	0.88	0.89	0.88

[a] Diffraction Weighted Dose as described by Zeldin *et al.*

[b] Values in parentheses are for the highest resolution shell.

[c] Half-dataset correlation coefficient, see: Karplus, P. A.; Diederichs, K. Linking crystallographic model and data quality. *Science* **2012**, *336*, 1030–1033.

S2.8. BCL6 ligand 8

Sweep	Sweep 1	Sweep 2	Sweep 3	Sweep 4	Sweep 5
**PDB Code**	**7GXA**	**7GXB**	**7GXC**	**7GXD**	**7GXE**
Dose (MGy)[a]	1.81	3.62	5.43	7.24	9.05
Space group	$P6_122$	$P6_122$	$P6_122$	$P6_122$	$P6_122$
Unit Cell (a=b,c) (Å)	67.69, 166.53	67.61, 166.48	67.67, 166.53	67.60, 166.48	67.78, 166.70
Unit Cell (α=β,γ) (°)	90.00, 120.00	90.00, 120.00	90.00, 120.00	90.00, 120.00	90.00, 120.00
Beamline	DLS I24	DLS I24	DLS I24	DLS I24	DLS I24
Wavelength (Å)	0.9686	0.9686	0.9686	0.9686	0.9686
Resolution (Å) [b]	33.94-1.95 (2.00-1.95)	33.92-1.95 (2.00-1.95)	33.94-1.95 (2.00-1.95)	33.92-1.95 (2.00-1.95)	33.98-1.95 (2.00-1.95)
Unique Reflections [b]	17317 (1187)	17221 (1189)	17308 (1184)	17211 (1187)	17376 (1197)
Multiplicity [b]	9.0 (9.3)	9.1 (9.3)	9.0 (9.3)	9.0 (9.3)	9.0 (9.3)
$R_{p.i.m}$ (%) [b]	2.2 (10.5)	4.2 (10.7)	3.1 (14.6)	3.0 (14.4)	2.5 (19.5)
R_{meas} (%) [b]	6.6 (32.4)	12.8 (33.6)	9.3 (44.7)	9.2 (44.7)	7.6 (59.6)
CC1/2 [b,c]	0.999 (0.970)	0.990 (0.962)	0.997 (0.955)	0.998 (0.938)	0.999 (0.917)
$I / \sigma(I)$ [b]	18.8 (6.3)	10.9 (5.8)	13.0 (4.8)	13.2 (4.5)	15.8 (3.7)
Completeness (%) [b]	100.0 (100.0)	100.0 (100.0)	100.0 (100.0)	100.0 (100.0)	100.0 (100.0)
Wilson B ($Å^2$)	20.6	20.9	22.5	22.3	24.5
**Refinement**					
R_{work} / R_{free}	17.86 / 20.53	17.81 / 20.61	17.77 / 20.73	17.68 / 20.76	17.54 / 20.23
No. Atoms					
Protein	1092	1092	1092	1092	1092
Ligand/ion	29	29	29	29	29
Water	168	168	168	168	168
Ramachandran (#,%)					
Allowed	120 (97.56%)	120 (97.56%)	120 (97.56%)	120 (97.56%)	120 (97.56%)
Generally allowed	3 (2.44%)	3 (2.44%)	3 (2.44%)	3 (2.44%)	3 (2.44%)
Disallowed	0 (0.00%)	0 (0.00%)	0 (0.00%)	0 (0.00%)	0 (0.00%)
B-factors					
Protein	28.16	28.28	30.42	30.75	33.15
Ligand/ion	28.42	28.52	31.01	31.38	33.74
Water	45.70	46.72	49.14	50.26	53.35
R.M.S. deviations					
Bond lengths (Å)	0.01	0.01	0.01	0.01	0.01
Bond angles (°)	0.93	0.93	0.93	0.93	0.92

[a] Diffraction Weighted Dose as described by Zeldin *et al.*

[b] Values in parentheses are for the highest resolution shell.

[c] Half-dataset correlation coefficient, see: Karplus, P. A.; Diederichs, K. Linking crystallographic model and data quality. *Science* **2012**, *336*, 1030–1033.

Sweep	Sweep 6	Sweep 7	Sweep 8	Sweep 9	Sweep 10
**PDB Code**	**7GXF**	**7GXG**	**7GXH**	**7GXI**	**7GXJ**
Dose (MGy)[a]	10.86	12.67	14.48	16.29	18.10
Space group	$P6_122$	$P6_122$	$P6_122$	$P6_122$	$P6_122$
Unit Cell (a=b,c) (Å)	67.61, 166.56	67.72, 166.64	67.62, 166.52	67.84, 166.76	67.61, 166.52
Unit Cell (α=β,γ) (°)	90.00, 120.00	90.00, 120.00	90.00, 120.00	90.00, 120.00	90.00, 120.00
Beamline	DLS I24	DLS I24	DLS I24	DLS I24	DLS I24
Wavelength (Å)	0.9686	0.9686	0.9686	0.9686	0.9686
Resolution (Å) [b]	33.93-1.95 (2.00-1.95)	33.96-1.95 (2.00-1.95)	33.93-1.95 (2.00-1.95)	34.00-1.95 (2.00-1.95)	33.93-1.95 (2.00-1.95)
Unique Reflections [b]	17227 (1188)	17351 (1201)	17223 (1187)	17419 (1209)	17223 (1188)
Multiplicity [b]	9.1 (9.3)	9.0 (9.3)	9.0 (9.2)	8.9 (9.2)	9.0 (9.3)
$R_{p.i.m}$ (%) [b]	3.9 (18.2)	3.5 (28.6)	3.5 (26.0)	3.1 (38.3)	4.3 (31.1)
R_{meas} (%) [b]	12.0 (56.8)	10.4 (87.5)	10.6 (80.9)	9.4 (117.5)	13.0 (96.9)
CC1/2 [b,c]	0.995 (0.916)	0.995 (0.845)	0.998 (0.844)	0.999 (0.781)	0.996 (0.794)
$I / \sigma(I)$ [b]	10.0 (3.7)	11.1 (2.7)	11.1 (2.6)	12.9 (1.9)	8.7 (2.3)
Completeness (%) [b]	100.0 (100.0)	100.0 (100.0)	100.0 (100.0)	100.0 (100.0)	100.0 (100.0)
Wilson B (Å^2)	25.3	26.8	26.5	29.1	29.1
**Refinement**					
R_{work} / R_{free}	17.58 / 20.13	17.81 / 20.71	17.77 / 20.84	17.92 / 20.12	17.65 / 20.49
No. Atoms					
Protein	1092	1092	1092	1092	1092
Ligand/ion	29	29	29	29	29
Water	168	168	168	168	168
Ramachandran (#,%)					
Allowed	120 (97.56%)	120 (97.56%)	120 (97.56%)	120 (97.56%)	120 (97.56%)
Generally allowed	3 (2.44%)	3 (2.44%)	3 (2.44%)	3 (2.44%)	3 (2.44%)
Disallowed	0 (0.00%)	0 (0.00%)	0 (0.00%)	0 (0.00%)	0 (0.00%)
B-factors					
Protein	33.70	35.02	35.35	37.20	36.97
Ligand/ion	34.07	35.67	35.98	38.15	37.60
Water	54.27	55.72	56.62	58.92	58.48
R.M.S. deviations					
Bond lengths (Å)	0.01	0.01	0.01	0.01	0.01
Bond angles (°)	0.93	0.93	0.92	0.93	0.93

[a] Diffraction Weighted Dose as described by Zeldin *et al.*
[b] Values in parentheses are for the highest resolution shell.
[c] Half-dataset correlation coefficient, see: Karplus, P. A.; Diederichs, K. Linking crystallographic model and data quality. *Science* **2012**, *336*, 1030–1033.

Sweep	Sweep 11	Sweep 12	Sweep 13	Sweep 14	Sweep 15
**PDB Code**	**7GXK**	**7GXL**	**7GXM**	**7GXN**	**7GXO**
Dose (MGy)[a]	19.91	21.72	23.53	25.34	27.15
Space group	$P6_122$	$P6_122$	$P6_122$	$P6_122$	$P6_122$
Unit Cell (a=b,c) (Å)	67.74, 166.66	67.63, 166.50	67.86, 166.74	67.60, 166.42	67.74, 166.62
Unit Cell (α=β,γ) (°)	90.00, 120.00	90.00, 120.00	90.00, 120.00	90.00, 120.00	90.00, 120.00
Beamline	DLS I24	DLS I24	DLS I24	DLS I24	DLS I24
Wavelength (Å)	0.9686	0.9686	0.9686	0.9686	0.9686
Resolution (Å) [b]	33.97-1.95 (2.00-1.95)	33.93-1.95 (2.00-1.95)	34.00-1.95 (2.00-1.95)	33.91-1.95 (2.00-1.95)	33.96-1.95 (2.00-1.95)
Unique Reflections [b]	17363 (1194)	17222 (1176)	17424 (1200)	17202 (1188)	17360 (1195)
Multiplicity [b]	8.9 (9.2)	9.0 (9.2)	8.9 (9.2)	9.0 (9.2)	8.9 (9.2)
$R_{p.i.m}$ (%) [b]	4.0 (51.0)	4.0 (38.7)	3.9 (62.9)	4.7 (41.1)	4.7 (74.3)
R_{meas} (%) [b]	12.1 (156.3)	12.3 (120.6)	11.7 (192.2)	14.2 (127.9)	14.3 (227.6)
CC1/2 [b,c]	0.999 (0.686)	0.998 (0.705)	0.999 (0.552)	0.996 (0.700)	0.998 (0.482)
$I / \sigma(I)$ [b]	9.4 (1.6)	9.4 (1.8)	10.7 (1.3)	7.9 (1.7)	8.2 (1.1)
Completeness (%) [b]	100.0 (100.0)	100.0 (100.0)	100.0 (100.0)	100.0 (100.0)	100.0 (100.0)
Wilson B ($Å^2$)	30.8	30.9	31.8	32.0	31.4
**Refinement**					
R_{work} / R_{free}	17.93 / 20.64	17.75 / 20.76	17.88 / 20.72	17.62 / 20.24	17.88 / 21.23
No. Atoms					
Protein	1092	1092	1092	1092	1092
Ligand/ion	29	29	29	29	29
Water	168	168	168	168	168
Ramachandran (#,%)					
Allowed	121 (98.37%)	120 (97.56%)	120 (97.56%)	119 (96.75%)	121 (98.37%)
Generally allowed	2 (1.63%)	3 (2.44%)	3 (2.44%)	4 (3.25%)	2 (1.63%)
Disallowed	0 (0.00%)	0 (0.00%)	0 (0.00%)	0 (0.00%)	0 (0.00%)
B-factors					
Protein	38.42	37.72	39.60	37.74	40.14
Ligand/ion	39.61	38.74	40.63	38.26	41.81
Water	60.55	59.25	61.37	58.95	62.23
R.M.S. deviations					
Bond lengths (Å)	0.01	0.01	0.01	0.01	0.01
Bond angles (°)	0.94	0.92	0.94	0.93	0.95

[a] Diffraction Weighted Dose as described by Zeldin *et al.*
[b] Values in parentheses are for the highest resolution shell.
[c] Half-dataset correlation coefficient, see: Karplus, P. A.; Diederichs, K. Linking crystallographic model and data quality. *Science* **2012**, *336*, 1030–1033.

S2.9. BCL6 ligand 9

Sweep	Sweep 1	Sweep 2	Sweep 3	Sweep 4	Sweep 5
**PDB Code**	**7GXP**	**7GXQ**	**7GXR**	**7GXS**	**7GXT**
Dose (MGy)[a]	1.42	2.84	4.26	5.69	7.11
Space group	$P6_122$	$P6_122$	$P6_122$	$P6_122$	$P6_122$
Unit Cell (a=b,c) (Å)	67.58, 165.83	67.45, 165.57	67.61, 165.88	67.51, 165.55	67.68, 166.12
Unit Cell (α=β,γ) (°)	90.00, 120.00	90.00, 120.00	90.00, 120.00	90.00, 120.00	90.00, 120.00
Beamline	DLS I24	DLS I24	DLS I24	DLS I24	DLS I24
Wavelength (Å)	0.9686	0.9686	0.9686	0.9686	0.9686
Resolution (Å) [b]	33.83-1.85 (1.89-1.85)	33.77-1.85 (1.89-1.85)	33.84-1.85 (1.89-1.85)	33.78-1.85 (1.89-1.85)	33.89-1.85 (1.89-1.85)
Unique Reflections [b]	18223 (1144)	19936 (1200)	18263 (1153)	19958 (1204)	18302 (1146)
Multiplicity [b]	9.9 (9.8)	9.1 (9.3)	9.9 (9.8)	9.1 (9.3)	9.9 (9.9)
$R_{p.i.m}$ (%) [b]	2.3 (14.9)	2.2 (11.9)	2.0 (15.5)	2.3 (15.1)	2.2 (22.7)
R_{meas} (%) [b]	7.6 (47.9)	6.9 (38.5)	6.7 (50.3)	7.4 (49.8)	7.3 (73.5)
CC1/2 [b,c]	0.999 (0.923)	0.999 (0.942)	0.999 (0.926)	0.999 (0.925)	0.999 (0.854)
I / σ(I) [b]	15.9 (4.4)	16.5 (4.6)	17.9 (4.3)	15.4 (4.0)	15.7 (2.9)
Completeness (%) [b]	92.8 (95.2)	100.0 (100.0)	92.9 (95.1)	100.0 (100.0)	92.8 (95.1)
Wilson B ($Å^2$)	19.3	20.6	20.9	21.7	22.9
**Refinement**					
R_{work} / R_{free}	17.92 / 21.34	18.13 / 21.28	17.51 / 20.87	18.11 / 21.82	18.05 / 21.41
No. Atoms					
Protein	1118	1118	1118	1118	1118
Ligand/ion	31	31	31	31	31
Water	170	170	170	170	170
Ramachandran (#,%)					
Allowed	119 (96.75%)	119 (96.75%)	119 (96.75%)	119 (96.75%)	119 (96.75%)
Generally allowed	4 (3.25%)	4 (3.25%)	4 (3.25%)	4 (3.25%)	4 (3.25%)
Disallowed	0 (0.00%)	0 (0.00%)	0 (0.00%)	0 (0.00%)	0 (0.00%)
B-factors					
Protein	28.24	28.61	29.78	30.02	32.56
Ligand/ion	29.21	29.41	30.79	30.91	33.38
Water	44.23	45.97	47.15	48.60	50.78
R.M.S. deviations					
Bond lengths (Å)	0.01	0.01	0.01	0.01	0.01
Bond angles (°)	0.94	0.96	0.94	0.95	0.95

[a] Diffraction Weighted Dose as described by Zeldin *et al.*

[b] Values in parentheses are for the highest resolution shell.

[c] Half-dataset correlation coefficient, see: Karplus, P. A.; Diederichs, K. Linking crystallographic model and data quality. *Science* **2012**, *336*, 1030–1033.

Sweep	Sweep 6	Sweep 7	Sweep 8	Sweep 9	Sweep 10
PDB Code	7GXU	7GXV	7GXW	7GXX	7GXY
Dose (MGy)[a]	8.53	9.95	11.37	12.79	14.22
Space group	$P6_122$	$P6_122$	$P6_122$	$P6_122$	$P6_122$
Unit Cell (a=b,c) (Å)	67.53, 165.62	67.68, 166.02	67.54, 165.55	67.73, 166.24	67.56, 165.61
Unit Cell (α=β,γ) (°)	90.00, 120.00	90.00, 120.00	90.00, 120.00	90.00, 120.00	90.00, 120.00
Beamline	DLS I24	DLS I24	DLS I24	DLS I24	DLS I24
Wavelength (Å)	0.9686	0.9686	0.9686	0.9686	0.9686
Resolution (Å) [b]	33.79-1.85 (1.89-1.85)	33.87-1.85 (1.89-1.85)	33.78-1.85 (1.89-1.85)	33.91-1.85 (1.89-1.85)	33.80-1.85 (1.89-1.85)
Unique Reflections [b]	19987 (1214)	18315 (1154)	19976 (1214)	18336 (1145)	20001 (1214)
Multiplicity [b]	9.0 (9.3)	9.9 (9.8)	9.0 (9.3)	9.9 (9.9)	9.0 (9.2)
$R_{p.i.m}$ (%) [b]	2.5 (20.1)	2.2 (25.5)	2.7 (23.2)	2.4 (34.8)	3.0 (30.1)
R_{meas} (%) [b]	8.0 (64.2)	7.2 (82.9)	8.6 (75.7)	8.0 (113.2)	9.5 (95.3)
CC1/2 [b,c]	0.999 (0.877)	0.999 (0.841)	0.999 (0.861)	0.999 (0.783)	0.999 (0.764)
$I / \sigma(I)$ [b]	14.1 (2.8)	16.0 (2.7)	13.1 (2.6)	14.4 (1.9)	12.1 (2.0)
Completeness (%) [b]	100.0 (100.0)	92.9 (95.2)	100.0 (100.0)	92.8 (95.3)	100.0 (100.0)
Wilson B ($Å^2$)	22.3	24.0	24.6	25.9	26.2
**Refinement**					
R_{work} / R_{free}	18.29 / 21.85	17.77 / 20.98	18.25 / 21.52	18.19 / 22.16	18.43 / 22.09
No. Atoms					
Protein	1118	1118	1118	1118	1118
Ligand/ion	31	31	31	31	31
Water	170	170	170	170	170
Ramachandran (#,%)					
Allowed	119 (96.75%)	119 (96.75%)	120 (97.56%)	119 (96.75%)	119 (96.75%)
Generally allowed	4 (3.25%)	4 (3.25%)	3 (2.44%)	4 (3.25%)	4 (3.25%)
Disallowed	0 (0.00%)	0 (0.00%)	0 (0.00%)	0 (0.00%)	0 (0.00%)
B-factors					
Protein	31.48	33.46	32.79	35.01	33.49
Ligand/ion	32.05	34.03	33.05	35.64	34.08
Water	50.91	52.37	52.77	54.14	53.78
R.M.S. deviations					
Bond lengths (Å)	0.01	0.01	0.01	0.01	0.01
Bond angles (°)	0.96	0.95	0.96	0.96	0.95

[a] Diffraction Weighted Dose as described by Zeldin *et al.*

[b] Values in parentheses are for the highest resolution shell.

[c] Half-dataset correlation coefficient, see: Karplus, P. A.; Diederichs, K. Linking crystallographic model and data quality. *Science* **2012**, *336*, 1030–1033.

Sweep	Sweep 11	Sweep 12	Sweep 13	Sweep 14	Sweep 15
**PDB Code**	**7GXZ**	**7GY0**	**7GY1**	**7GY2**	**7GY3**
Dose (MGy)[a]	15.64	17.06	18.48	19.90	21.32
Space group	$P6_122$	$P6_122$	$P6_122$	$P6_122$	$P6_122$
Unit Cell (a=b,c) (Å)	67.71, 166.06	67.55, 165.57	67.76, 166.29	67.57, 165.58	67.74, 166.08
Unit Cell (α=β,γ) (°)	90.00, 120.00	90.00, 120.00	90.00, 120.00	90.00, 120.00	90.00, 120.00
Beamline	DLS I24	DLS I24	DLS I24	DLS I24	DLS I24
Wavelength (Å)	0.9686	0.9686	0.9686	0.9686	0.9686
Resolution (Å) [b]	33.88-1.85 (1.89-1.85)	33.79-1.85 (1.89-1.85)	33.92-1.85 (1.89-1.85)	33.79-1.85 (1.89-1.85)	33.89-1.85 (1.89-1.85)
Unique Reflections [b]	18328 (1149)	19984 (1212)	18352 (1138)	20001 (1214)	18342 (1145)
Multiplicity [b]	9.9 (9.9)	9.0 (9.2)	9.9 (9.8)	9.0 (9.2)	9.9 (9.9)
$R_{p.i.m}$ (%) [b]	2.4 (37.5)	3.1 (34.1)	2.7 (51.4)	3.4 (38.4)	2.8 (56.0)
R_{meas} (%) [b]	8.0 (122.6)	9.8 (110.2)	9.0 (168.4)	10.8 (121.4)	9.2 (183.6)
CC1/2 [b,c]	0.999 (0.765)	0.999 (0.748)	0.999 (0.675)	0.999 (0.686)	0.999 (0.652)
$I / \sigma(I)$ [b]	14.5 (1.8)	11.3 (1.8)	12.7 (1.3)	10.4 (1.6)	12.5 (1.1)
Completeness (%) [b]	92.9 (95.1)	100.0 (100.0)	92.8 (95.3)	100.0 (100.0)	92.9 (95.0)
Wilson B (Å^2)	26.5	28.0	28.4	28.3	28.2
**Refinement**					
R_{work} / R_{free}	18.20 / 21.85	18.19 / 21.60	18.40 / 21.33	18.33 / 22.05	18.26 / 21.43
No. Atoms					
Protein	1118	1118	1118	1118	1118
Ligand/ion	31	31	31	31	31
Water	170	170	170	170	170
Ramachandran (#,%)					
Allowed	119 (96.75%)	119 (96.75%)	119 (96.75%)	120 (97.56%)	119 (96.75%)
Generally allowed	4 (3.25%)	4 (3.25%)	4 (3.25%)	3 (2.44%)	4 (3.25%)
Disallowed	0 (0.00%)	0 (0.00%)	0 (0.00%)	0 (0.00%)	0 (0.00%)
B-factors					
Protein	35.41	34.66	36.69	34.92	37.16
Ligand/ion	35.87	35.00	37.40	34.91	37.61
Water	54.88	55.28	56.35	55.88	56.92
R.M.S. deviations					
Bond lengths (Å)	0.01	0.01	0.01	0.01	0.01
Bond angles (°)	0.95	0.96	0.97	0.95	0.96

[a] Diffraction Weighted Dose as described by Zeldin *et al.*

[b] Values in parentheses are for the highest resolution shell.

[c] Half-dataset correlation coefficient, see: Karplus, P. A.; Diederichs, K. Linking crystallographic model and data quality. *Science* **2012**, *336*, 1030–1033.

S2.10. HSP72 ligand 10

Sweep	Sweep 1	Sweep 2	Sweep 3	Sweep 4	Sweep 5
**PDB Code**	**7GY4**	**7GY5**	**7GY6**	**7GY7**	**7GY8**
Dose (MGy)[a]	1.14	2.28	3.42	4.56	5.70
Space group	$P2_12_12_1$	$P2_12_12_1$	$P2_12_12_1$	$P2_12_12_1$	$P2_12_12_1$
Unit Cell (a,b,c) (Å)	47.74, 89.31, 96.70	47.76, 89.36, 96.88	47.80, 89.41, 96.81	47.83, 89.47, 97.00	47.85, 89.50, 96.90
Unit Cell (α=β=γ) (°)	90.00	90.00	90.00	90.00	90.00
Beamline	DLS I24	DLS I24	DLS I24	DLS I24	DLS I24
Wavelength (Å)	0.9192	0.9192	0.9192	0.9192	0.9192
Resolution (Å) [b]	38.60-1.92 (1.97-1.92)	38.63-1.92 (1.97-1.92)	38.65-1.92 (1.97-1.92)	38.68-1.92 (1.97-1.92)	38.69-1.92 (1.97-1.92)
Unique Reflections [b]	32381 (2128)	32469 (2142)	32478 (2135)	32592 (2139)	32576 (2138)
Multiplicity [b]	6.3 (6.4)	6.3 (6.4)	6.3 (6.4)	6.3 (6.4)	6.3 (6.4)
$R_{p.i.m}$ (%) [b]	4.0 (12.5)	4.1 (16.0)	4.0 (15.3)	4.3 (20.1)	4.1 (18.9)
R_{meas} (%) [b]	10.0 (31.9)	10.2 (40.6)	10.0 (39.1)	10.8 (51.1)	10.2 (48.1)
CC1/2 [b,c]	0.993 (0.955)	0.996 (0.927)	0.994 (0.933)	0.996 (0.89)	0.995 (0.889)
$I / \sigma(I)$ [b]	11.4 (5.4)	10.8 (4.5)	10.9 (4.5)	9.9 (3.6)	10.1 (3.7)
Completeness (%) [b]	100.0 (100.0)	100.0 (100.0)	100.0 (100.0)	100.0 (100.0)	100.0 (100.0)
Wilson B (Å^2)	18.9	19.8	21.3	21.8	23.1
**Refinement**					
R_{work} / R_{free}	16.93 / 21.47	16.93 / 21.79	16.84 / 21.65	16.77 / 21.62	16.75 / 21.51
No. Atoms					
Protein	3031	3031	3031	3031	3031
Ligand/ion	35	35	35	35	35
Water	492	492	492	492	492
Ramachandran (#,%)					
Allowed	382 (98.45%)	383 (98.71%)	384 (98.97%)	383 (98.71%)	383 (98.71%)
Generally allowed	6 (1.55%)	5 (1.29%)	4 (1.03%)	5 (1.29%)	5 (1.29%)
Disallowed	0 (0.00%)	0 (0.00%)	0 (0.00%)	0 (0.00%)	0 (0.00%)
B-factors					
Protein	27.30	27.67	29.05	29.80	31.09
Ligand/ion	36.56	37.37	38.97	40.64	42.26
Water	38.08	39.17	41.35	42.85	44.73
R.M.S. deviations					
Bond lengths (Å)	0.01	0.01	0.01	0.01	0.01
Bond angles (°)	0.99	0.99	0.98	0.99	0.99

[a] Diffraction Weighted Dose as described by Zeldin *et al.*

[b] Values in parentheses are for the highest resolution shell.

[c] Half-dataset correlation coefficient, see: Karplus, P. A.; Diederichs, K. Linking crystallographic model and data quality. *Science* **2012**, *336*, 1030–1033.

Sweep	Sweep 6	Sweep 7	Sweep 8	Sweep 9	Sweep 10
**PDB Code**	**7GY9**	**7GYA**	**7GYB**	**7GYC**	**7GYD**
Dose (MGy)[a]	6.84	7.98	9.12	10.26	11.40
Space group	$P2_12_12_1$	$P2_12_12_1$	$P2_12_12_1$	$P2_12_12_1$	$P2_12_12_1$
Unit Cell (a,b,c) (Å)	47.87, 89.56, 97.07	47.90, 89.60, 97.00	47.93, 89.67, 97.18	47.95, 89.71, 97.11	47.97, 89.74, 97.23
Unit Cell (α=β=γ) (°)	90.00	90.00	90.00	90.00	90.00
Beamline	DLS I24	DLS I24	DLS I24	DLS I24	DLS I24
Wavelength (Å)	0.9192	0.9192	0.9192	0.9192	0.9192
Resolution (Å) [b]	38.72-1.92 (1.97-1.92)	38.73-1.92 (1.97-1.92)	38.76-1.92 (1.97-1.92)	38.77-1.92 (1.97-1.92)	38.79-1.92 (1.97-1.92)
Unique Reflections [b]	32670 (2136)	32672 (2134)	32784 (2134)	32780 (2132)	32851 (2142)
Multiplicity [b]	6.3 (6.4)	6.3 (6.4)	6.3 (6.4)	6.3 (6.4)	6.3 (6.4)
$R_{p.i.m}$ (%) [b]	4.6 (25.6)	4.3 (24.0)	5.1 (32.2)	4.5 (29.6)	5.8 (40.6)
R_{meas} (%) [b]	11.5 (64.9)	10.7 (61.0)	12.8 (81.8)	11.3 (75.2)	14.5 (103.1)
CC1/2 [b,c]	0.996 (0.816)	0.995 (0.814)	0.996 (0.685)	0.996 (0.712)	0.995 (0.552)
I / σ(I) [b]	9.1 (3.0)	9.4 (2.9)	8.2 (2.4)	8.8 (2.4)	7.2 (1.9)
Completeness (%) [b]	100.0 (100.0)	100.0 (100.0)	100.0 (100.0)	100.0 (100.0)	100.0 (100.0)
Wilson B ($Å^2$)	23.8	25.4	25.6	27.9	29.2
**Refinement**					
R_{work} / R_{free}	16.73 / 21.44	16.80 / 21.61	16.82 / 21.69	16.94 / 21.86	17.26 / 22.51
No. Atoms					
Protein	3031	3031	3031	3031	3031
Ligand/ion	35	35	35	35	35
Water	492	492	492	492	492
Ramachandran (#,%)					
Allowed	383 (98.71%)	383 (98.71%)	383 (98.71%)	383 (98.71%)	383 (98.71%)
Generally allowed	5 (1.29%)	5 (1.29%)	5 (1.29%)	5 (1.29%)	5 (1.29%)
Disallowed	0 (0.00%)	0 (0.00%)	0 (0.00%)	0 (0.00%)	0 (0.00%)
B-factors					
Protein	32.11	33.56	34.17	36.09	36.58
Ligand/ion	43.91	46.57	47.58	36.47	36.69
Water	46.58	48.33	49.80	51.83	52.64
R.M.S. deviations					
Bond lengths (Å)	0.01	0.01	0.01	0.01	0.01
Bond angles (°)	0.99	0.99	0.99	0.99	1.00

[a] Diffraction Weighted Dose as described by Zeldin *et al.*

[b] Values in parentheses are for the highest resolution shell.

[c] Half-dataset correlation coefficient, see: Karplus, P. A.; Diederichs, K. Linking crystallographic model and data quality. *Science* **2012**, *336*, 1030–1033.

Sweep	Sweep 11	Sweep 12	Sweep 13	Sweep 14	Sweep 15
**PDB Code**	**7GYE**	**7GYF**	**7GYG**	**7GYH**	**7GYI**
Dose (MGy)[a]	12.54	13.68	14.82	15.96	17.10
Space group	$P2_12_12_1$	$P2_12_12_1$	$P2_12_12_1$	$P2_12_12_1$	$P2_12_12_1$
Unit Cell (a,b,c) (Å)	47.98, 89.79, 97.19	48.09, 89.87, 97.43	48.02, 89.86, 97.27	48.04, 89.89, 97.33	48.05, 89.87, 97.31
Unit Cell (α=β=γ) (°)	90.00	90.00	90.00	90.00	90.00
Beamline	DLS I24	DLS I24	DLS I24	DLS I24	DLS I24
Wavelength (Å)	0.9192	0.9192	0.9192	0.9192	0.9192
Resolution (Å) [b]	38.80-1.92 (1.97-1.92)	38.88-1.92 (1.97-1.92)	38.83-1.92 (1.97-1.92)	38.85-1.92 (1.97-1.92)	38.85-1.92 (1.97-1.92)
Unique Reflections [b]	32871 (2146)	33062 (2185)	32939 (2139)	32987 (2163)	32976 (2158)
Multiplicity [b]	6.3 (6.4)	6.3 (6.4)	6.3 (6.4)	6.3 (6.3)	6.3 (6.4)
$R_{p.i.m}$ (%) [b]	4.8 (37.9)	6.4 (53.3)	5.1 (42.9)	7.3 (65.8)	5.5 (50.3)
R_{meas} (%) [b]	12.0 (96.3)	16.1 (134.8)	12.8 (109.1)	18.4 (166.6)	13.9 (127.9)
CC1/2 [b,c]	0.996 (0.525)	0.994 (0.369)	0.996 (0.390)	0.994 (0.207)	0.996 (0.245)
$I / \sigma(I)$ [b]	8.1 (1.9)	6.6 (1.6)	7.7 (1.6)	6.0 (1.3)	7.2 (1.4)
Completeness (%) [b]	100.0 (100.0)	100.0 (100.0)	100.0 (100.0)	100.0 (100.0)	100.0 (100.0)
Wilson B (Å^2)	30.3	32.1	33.3	25.6	36.2
**Refinement**					
R_{work} / R_{free}	17.09 / 22.15	17.49 / 23.03	17.29 / 22.86	17.73 / 23.51	17.44 / 22.55
No. Atoms					
Protein	3031	3031	3031	3031	3031
Ligand/ion	35	35	35	35	35
Water	492	492	492	492	492
Ramachandran (#,%)					
Allowed	384 (98.97%)	384 (98.97%)	385 (99.23%)	386 (99.48%)	384 (98.97%)
Generally allowed	4 (1.03%)	4 (1.03%)	3 (0.77%)	2 (0.52%)	4 (1.03%)
Disallowed	0 (0.00%)	0 (0.00%)	0 (0.00%)	0 (0.00%)	0 (0.00%)
B-factors					
Protein	38.61	39.16	41.24	41.22	43.42
Ligand/ion	39.63	40.24	42.28	43.06	43.85
Water	54.65	55.25	57.91	57.95	59.48
R.M.S. deviations					
Bond lengths (Å)	0.01	0.01	0.01	0.01	0.01
Bond angles (°)	1.00	0.99	1.01	1.01	1.01

[a] Diffraction Weighted Dose as described by Zeldin *et al.*

[b] Values in parentheses are for the highest resolution shell.

[c] Half-dataset correlation coefficient, see: Karplus, P. A.; Diederichs, K. Linking crystallographic model and data quality. *Science* **2012**, *336*, 1030–1033.

S2.11. HSP72 ligand 11

Sweep	Sweep 1	Sweep 2	Sweep 3	Sweep 4	Sweep 5
**PDB Code**	**7GYJ**	**7GYK**	**7GYL**	**7GYM**	**7GYN**
Dose (MGy)[a]	1.43	2.86	4.29	5.72	7.15
Space group	$P2_12_12_1$	$P2_12_12_1$	$P2_12_12_1$	$P2_12_12_1$	$P2_12_12_1$
Unit Cell (a,b,c) (Å)	52.07, 82.11, 93.31	52.10, 82.07, 93.40	52.08, 82.21, 93.45	52.15, 82.14, 93.52	52.10, 82.30, 93.58
Unit Cell (α=β,γ) (°)	90.00	90.00	90.00	90.00	90.00
Beamline	DLS I24	DLS I24	DLS I24	DLS I24	DLS I24
Wavelength (Å)	0.9192	0.9192	0.9192	0.9192	0.9192
Resolution (Å) [b]	39.78-2.15 (2.22-2.15)	39.79-2.15 (2.22-2.15)	39.80-2.15 (2.22-2.15)	39.83-2.15 (2.22-2.15)	39.83-2.15 (2.22-2.15)
Unique Reflections [b]	22462 (1913)	22479 (1909)	22531 (1922)	22560 (1928)	22587 (1924)
Multiplicity [b]	6.3 (6.3)	6.3 (6.3)	6.3 (6.3)	6.3 (6.3)	6.3 (6.3)
$R_{p.i.m}$ (%) [b]	2.4 (7.8)	2.5 (10.1)	2.6 (10.2)	2.9 (13.5)	3.1 (14.4)
R_{meas} (%) [b]	6.0 (19.8)	6.3 (25.5)	6.7 (25.9)	7.3 (34.0)	7.8 (36.3)
CC1/2 [b,c]	0.999 (0.983)	0.999 (0.976)	0.999 (0.976)	0.999 (0.963)	0.998 (0.953)
$I / \sigma(I)$ [b]	18.4 (8.0)	18.2 (6.6)	16.4 (6.3)	15.4 (5.2)	13.8 (4.8)
Completeness (%) [b]	100.0 (100.0)	100.0 (100.0)	100.0 (100.0)	100.0 (100.0)	100.0 (100.0)
Wilson B (Å^2)	22.6	22.7	23.9	24.6	26.4
**Refinement**					
R_{work} / R_{free}	16.13 / 20.39	16.17 / 20.45	16.10 / 20.48	16.19 / 20.69	16.13 / 20.94
No. Atoms					
Protein	2983	2983	2983	2983	2983
Ligand/ion	92	92	92	92	92
Water	319	319	319	319	319
Ramachandran (#,%)					
Allowed	379 (98.96%)	377 (98.43%)	378 (98.69%)	377 (98.43%)	377 (98.43%)
Generally allowed	4 (1.04%)	6 (1.57%)	5 (1.31%)	6 (1.57%)	6 (1.57%)
Disallowed	0 (0.00	0 (0.00%)	0 (0.00%)	0 (0.00%)	0 (0.00%)
B-factors					
Protein	30.80	31.40	32.41	32.90	34.71
Ligand/ion	44.48	46.24	47.70	49.62	52.08
Water	40.72	42.00	43.61	44.79	47.50
R.M.S. deviations					
Bond lengths (Å)	0.01	0.01	0.01	0.01	0.01
Bond angles (°)	0.99	0.99	0.99	0.99	0.99

[a] Diffraction Weighted Dose as described by Zeldin *et al.*

[b] Values in parentheses are for the highest resolution shell.

[c] Half-dataset correlation coefficient, see: Karplus, P. A.; Diederichs, K. Linking crystallographic model and data quality. *Science* **2012**, *336*, 1030–1033.

Sweep	Sweep 6	Sweep 7	Sweep 8	Sweep 9	Sweep 10
**PDB Code**	**7GYO**	**7GYP**	**7GYQ**	**7GYR**	**7GYS**
Dose (MGy)[a]	8.58	10.01	11.44	12.87	14.30
Space group	$P2_12_12_1$	$P2_12_12_1$	$P2_12_12_1$	$P2_12_12_1$	$P2_12_12_1$
Unit Cell (a=b,c) (Å)	52.20, 82.19, 93.59	52.10, 82.36, 93.70	52.21, 82.26, 93.68	52.12, 82.43, 93.82	52.22, 82.32, 93.76
Unit Cell (α=β,γ) (°)	90.00	90.00	90.00	90.00	90.00
Beamline	DLS I24	DLS I24	DLS I24	DLS I24	DLS I24
Wavelength (Å)	0.9192	0.9192	0.9192	0.9192	0.9192
Resolution (Å) [b]	39.87-2.15 (2.22-2.15)	39.85-2.15 (2.22-2.15)	39.89-2.15 (2.22-2.15)	39.87-2.15 (2.22-2.15)	39.90-2.15 (2.22-2.15)
Unique Reflections [b]	22606 (1933)	22636 (1926)	22642 (1926)	22705 (1950)	22683 (1930)
Multiplicity [b]	6.3 (6.3)	6.3 (6.3)	6.3 (6.3)	6.3 (6.3)	6.3 (6.3)
$R_{p.i.m}$ (%) [b]	3.5 (18.6)	3.5 (19.6)	3.9 (26.1)	4.1 (29.3)	4.6 (38.4)
R_{meas} (%) [b]	8.8 (47.2)	8.9 (49.7)	10.0 (65.7)	10.4 (74.0)	11.6 (96.9)
CC1/2 [b,c]	0.998 (0.922)	0.998 (0.912)	0.998 (0.813)	0.998 (0.848)	0.998 (0.759)
$I / \sigma(I)$ [b]	12.5 (4.0)	12.2 (3.6)	11.1 (3.0)	10.1 (2.6)	9.6 (2.1)
Completeness (%) [b]	100.0 (100.0)	100.0 (100.0)	100.0 (100.0)	100.0 (100.0)	100.0 (100.0)
Wilson B (Å^2)	26.8	30.3	30.0	33.5	32.4
**Refinement**					
R_{work} / R_{free}	16.22 / 20.56	16.34 / 20.85	16.45 / 21.53	16.54 / 21.22	16.65 / 21.40
No. Atoms					
Protein	2983	2983	2983	2983	2983
Ligand/ion	92	92	92	92	92
Water	319	319	319	319	319
Ramachandran (#,%)					
Allowed	378 (98.69%)	377 (98.43%)	380 (99.22%)	379 (98.96%)	380 (99.22%)
Generally allowed	5 (1.31%)	6 (1.57%)	3 (0.78%)	4 (1.04%)	3 (0.78%)
Disallowed	0 (0.00%)	0 (0.00%)	0 (0.00%)	0 (0.00%)	0 (0.00%)
B-factors					
Protein	34.78	37.75	37.96	40.88	41.38
Ligand/ion	52.70	56.03	57.17	59.44	56.82
Water	48.16	52.10	52.72	55.73	56.82
R.M.S. deviations					
Bond lengths (Å)	0.01	0.01	0.01	0.01	0.01
Bond angles (°)	0.99	0.99	0.98	0.99	1.00

[a] Diffraction Weighted Dose as described by Zeldin *et al.*

[b] Values in parentheses are for the highest resolution shell.

[c] Half-dataset correlation coefficient, see: Karplus, P. A.; Diederichs, K. Linking crystallographic model and data quality. *Science* **2012**, *336*, 1030–1033.

Sweep	Sweep 11	Sweep 12	Sweep 13	Sweep 14	Sweep 15
PDB Code	7GYT	7GYU	7GYV	7GYW	7GYX
Dose (MGy)[a]	15.73	17.16	18.59	20.02	21.45
Space group	$P2_12_12_1$	$P2_12_12_1$	$P2_12_12_1$	$P2_12_12_1$	$P2_12_12_1$
Unit Cell (a=b,c) (Å)	52.13, 82.51, 93.94	52.26, 82.39, 93.87	52.14, 82.58, 94.06	52.26, 82.45, 93.96	52.13, 82.63, 94.13
Unit Cell (α=β,γ) (°)	90.00	90.00	90.00	90.00	90.00
Beamline	DLS I24	DLS I24	DLS I24	DLS I24	DLS I24
Wavelength (Å)	0.9192	0.9192	0.9192	0.9192	0.9192
Resolution (Å) [b]	39.90-2.15 (2.22-2.15)	39.94-2.15 (2.22-2.15)	39.92-2.15 (2.22-2.15)	39.95-2.15 (2.22-2.15)	39.93-2.15 (2.22-2.15)
Unique Reflections [b]	22752 (1934)	22754 (1931)	22797 (1940)	22792 (1936)	22826 (1946)
Multiplicity [b]	6.3 (6.3)	6.3 (6.3)	6.3 (6.3)	6.3 (6.3)	6.3 (6.3)
$R_{p.i.m}$ (%) [b]	4.9 (44.7)	5.2 (55.5)	5.9 (71.2)	5.9 (78.3)	7.2 (115.5)
R_{meas} (%) [b]	12.4 (113.4)	13.3 (140.1)	15.0 (179.4)	14.8 (197.2)	18.3 (290.7)
CC1/2 [b,c]	0.997 (0.713)	0.997 (0.579)	0.997 (0.523)	0.997 (0.471)	0.996 (0.267)
$I / \sigma(I)$ [b]	8.4 (1.9)	8.2 (1.5)	7.3 (1.3)	7.5 (1.1)	6.3 (0.9)
Completeness (%) [b]	100.0 (100.0)	100.0 (100.0)	100.0 (100.0)	100.0 (100.0)	100.0 (100.0)
Wilson B ($Å^2$)	35.8	34.7	35.8	39.1	38.9
**Refinement**					
R_{work} / R_{free}	16.73 / 22.44	17.02 / 22.35	17.13 / 22.20	17.39 / 22.88	17.44 / 23.21
No. Atoms					
Protein	2983	2983	2983	2983	2983
Ligand/ion	92	92	92	92	92
Water	319	319	319	319	319
Ramachandran (#,%)					
Allowed	379 (98.96%)	381 (99.48%)	378 (98.69%)	380 (99.22%)	379 (98.96%)
Generally allowed	4 (1.04%)	2 (0.52%)	5 (1.31%)	3 (0.78%)	4 (1.04%)
Disallowed	0 (0.00%)	0 (0.00%)	0 (0.00%)	0 (0.00%)	0 (0.00%)
B-factors					
Protein	43.58	45.22	47.30	49.05	50.35
Ligand/ion	54.34	51.03	58.99	56.92	58.03
Water	59.17	60.71	63.28	63.96	66.55
R.M.S. deviations					
Bond lengths (Å)	0.01	0.01	0.01	0.01	0.01
Bond angles (°)	1.01	1.01	1.01	1.02	1.02

[a] Diffraction Weighted Dose as described by Zeldin *et al.*

[b] Values in parentheses are for the highest resolution shell.

[c] Half-dataset correlation coefficient, see: Karplus, P. A.; Diederichs, K. Linking crystallographic model and data quality. *Science* **2012**, *336*, 1030–1033.
